# Thalamocortical circuits drive remifentanil-induced postoperative hyperalgesia

**DOI:** 10.1172/JCI158742

**Published:** 2022-12-15

**Authors:** Yan Jin, Yu Mao, Danyang Chen, Yingju Tai, Rui Hu, Chen-Ling Yang, Jing Zhou, Lijian Chen, Xuesheng Liu, Erwei Gu, Chunhui Jia, Zhi Zhang, Wenjuan Tao

**Affiliations:** 1 Stroke Center and Department of Neurology and; 2 Department of Anesthesiology and Pain Medicine, The First Affiliated Hospital of University of Science and Technology of China (USTC), Division of Life Sciences and Medicine, USTC, Hefei, China.; 3 Department of Anesthesiology, The First Affiliated Hospital of Anhui Medical University, Hefei, China.; 4 Department of Anesthesiology, The Third Affiliated Hospital of Anhui Medical University, Hefei, China.; 5 Department of Physiology, School of Basic Medical Sciences, Anhui Medical University, Hefei, China.; 6 Department of head, neck, and breast Surgery, Western district of the First Affiliated Hospital of USTC, Division of Life Sciences and Medicine, USTC, Hefei, China.

**Keywords:** Neuroscience, Anesthesiology, Pain

## Abstract

Remifentanil-induced hyperalgesia (RIH) is a severe but common postoperative clinical problem with elusive underlying neural mechanisms. Here, we discovered that glutamatergic neurons in the thalamic ventral posterolateral nucleus (VPL^Glu^) exhibited significantly elevated burst firing accompanied by upregulation of Ca_v_3.1 T-type calcium channel expression and function in RIH model mice. In addition, we identified a glutamatergic neuronal thalamocortical circuit in the VPL projecting to hindlimb primary somatosensory cortex glutamatergic neurons (S1HL^Glu^) that mediated RIH. In vivo calcium imaging and multi-tetrode recordings revealed heightened S1HL^Glu^ neuronal activity during RIH. Moreover, preoperative suppression of Ca_v_3.1-dependent burst firing in VPL^Glu^ neurons or chemogenetic inhibition of VPL^Glu^ neuronal terminals in the S1HL abolished the increased S1HL^Glu^ neuronal excitability while alleviating RIH. Our findings suggest that remifentanil induces postoperative hyperalgesia by upregulating T-type calcium channel-dependent burst firing in VPL^Glu^ neurons to activate S1HL^Glu^ neurons, thus revealing an ion channel–mediated neural circuit basis for RIH that can guide analgesic development.

## Graphical abstract

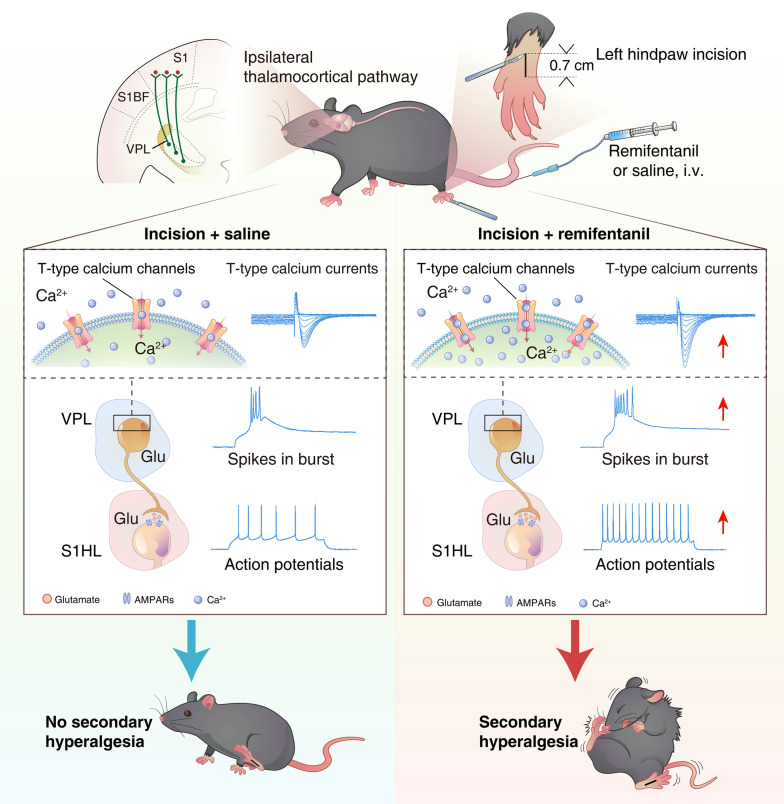

## Introduction

Remifentanil is an ultra-fast-acting opioid analgesic that has been widely adopted in clinical anesthesia. Because it can rapidly elicit onset and offset of analgesia, remifentanil is central to the termination of infusion during surgery, thus facilitating postanesthesia recovery (1). However, as a potent agonist of μ-opioid receptors (MORs) with a rapid hit-and-go temporal profile, its use has long been associated with the development of remifentanil-induced hyperalgesia (RIH), a paradoxical phenomenon in which a patient treated with opioids for intraoperative pain control may display enhanced postoperative sensitivity to painful stimuli (2–5). Both clinical and preclinical animal model studies have demonstrated that remifentanil treatment can generate and strengthen sensitization to postoperative pain, which is clinically manifested as enhanced pain intensity, increased opioid consumption, and aggravation of the adverse side effects of opioids (4–6). In addition to these issues, acute pain that is not effectively controlled may become chronic pain (7, 8). Since the strategies for prevention and treatment of RIH are urgently needed in the clinic, research attention is increasingly focused on determining its underlying mechanisms.

It is well known that the thalamocortical circuit within the central nervous system is required for discriminating the location, intensity, and quality of noxious stimuli (9) and plays a key role in the sensing of pain (3, 10, 11). The ventral posterolateral nucleus (VPL), a higher-order thalamocortical structure, is the somatosensory gateway that distributes nociceptive information from the spinothalamic tract to the appropriate circuits in the cerebral cortex (9). In addition, previous studies in various animal models for pain sensing, including inflammatory and neuropathic pain, have reported alterations in VPL neuronal activity (12–14). Functional magnetic resonance imaging (fMRI) studies have demonstrated that the cerebral blood flow in the VPL of human volunteers was enhanced after remifentanil suspension (15, 16). Together, these studies indicate that the VPL is likely crucial for development of RIH.

The VPL primarily contains glutamatergic neurons that exhibit prominent tonic and burst firing patterns (17, 18). Burst firing is a unique mode of neuronal firing activity that occurs in the thalamus and plays an essential role in pain signal transmission (19–21). Neuronal burst firing is mediated by T-type calcium 3.1 (Ca_v_3.1) channels, a highly expressed subtype of low-voltage-activated T-type calcium channel in thalamocortical projection neurons (13, 21, 22). In animal models, Ca_v_3.1 knockout can attenuate spontaneous and mechanical pain in mice with spinal neuropathic pain (23) or trigeminal neuropathic pain (24), collectively suggesting that dysfunction of Ca_v_3.1 channels may result in aberrant thalamocortical dynamics associated with the neuropathy of chronic pain (22). In light of these findings, next, it is necessary to determine how the spike patterns of VPL neurons may be altered during RIH as well as how Ca_v_3.1 channels contribute to activity of the thalamocortical circuits for RIH.

In the present study, we used viral tracing, in vivo and in vitro electrophysiological recordings, 2-photon calcium imaging, fiber photometry, as well as optogenetic and chemogenetic treatments to demonstrate that intraoperative infusion of remifentanil (~1.3 μg/kg/min) enhances the function of Ca_v_3.1 channels, leading to an increase in the burst activity of glutamatergic neurons in the VPL (VPL^Glu^) of a RIH mouse model induced by plantar incision. This increased burst firing in VPL^Glu^ neurons results in the hyperactivity of glutamatergic neurons in the hindlimb primary somatosensory cortex (S1HL^Glu^). These findings provide strong evidence that RIH in mice was induced by overactivity of a thalamocortical pathway from VPL^Glu^ neurons to S1HL^Glu^ neurons that was dependent on enhanced burst firing in VPL^Glu^ neurons. Our study further revealed that elevated T-type channel activity stimulates the VPL^Glu^→S1HL^Glu^ pathway, leading to the development of RIH and providing a potential ion channel-mediated mechanism for postoperative pain associated with remifentanil treatment.

## Results

### Remifentanil induced postoperative hyperalgesia in mice.

**Figure 1:**
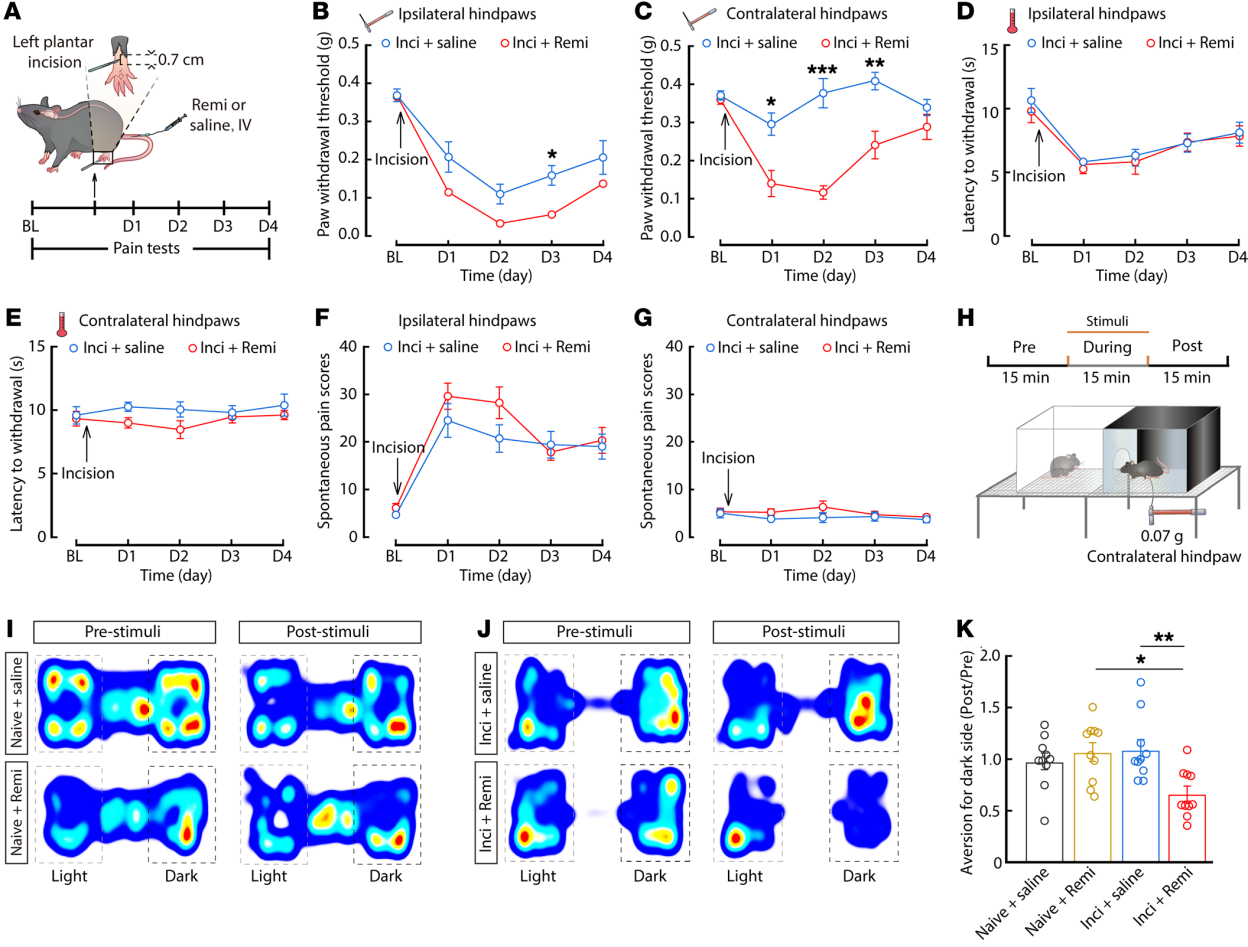
Remifentanil induces postoperative hyperalageisa in incisional mice. (**A**) Schematic of the experimental procedure for surgery and behavioral tests of mice. (**B** and **C**) Time course of changes in the response threshold to mechanical force assessed using von Frey tests in ipsilateral (**B**, *F*_(1,21)_ = 16.14, *P* = 0.0006) and contralateral (**C**, *F*_(1,21)_ = 52.68, *P* < 0.0001) hindpaws of mice with plantar incision infused with remi (inci + remi) or saline (inci + saline) (inci + saline, *n* = 11 mice; inci + remi, *n* = 12 mice). (**D** and **E**) Time course of changes in the response to thermal pain assessed using Hargreaves tests in ipsilateral (**D**, *F*_(1,17)_ = 0.9832, *P* = 0.3353) and contralateral (**E**, *F*_(1,17)_ = 5.323, *P* = 0.0339) hindpaws in inci + remi and inci + saline mice (inci + saline, *n* = 10; inci + remi, *n* = 9 mice). (**F** and **G**) Time course of spontaneous pain scores of ipsilateral (**F***, F*_(1,18)_ = 3.308, *P* = 0.0856) and contralateral (**G***, F*_(1,18)_ = 6.135, *P* = 0.0234) hindpaws in inci + remi and inci + saline mice (*n* = 10 mice per group). (**H**) Schematic for RT-PEAP tests. (**I** and **J**) Heatmaps showing the locations of naive and incisional mice treated with saline or remifentanil in RT-PEAP tests. (**K**) Summary of data from von Frey filament stimulus-induced place aversion (*n* = 10 mice per group; *F*_(3,36)_ = 5.113, *P* = 0.0047). Data are presented as mean ± SEM. *P < 0.05, **P < 0.01, ***P < 0.001. 2-way repeated measures ANOVA with post hoc Bonferroni’s test in (**B**–**G**); 1-way ANOVA with post hoc Bonferroni’s test in (**K**).

We first established a mouse model for RIH by infusion of remifentanil (40 μg/kg, 1.3 μg/kg/min) through the tail vein for 30 minutes in mice receiving left (ipsilateral) plantar incision (25) (Figure 1A). Compared with incisional mice treated with saline (inci + saline), the administration of remifentanil to incisional mice (inci + remi) resulted in a nonsignificant decrease in the mechanical threshold of the ipsilateral hindpaws during the first 2 days after surgery in von Frey tests (Figure 1B), potentially due to an inability to distinguish the degree of pain under the hypersensitivity induced by the incision trauma. Interestingly, von Frey tests showed a significant postoperative decrease in the mechanical threshold of the right (contralateral) hindpaws of inci + remi mice compared with that in inci + saline mice (Figure 1C). Concurrently, we found no obvious difference in mechanical threshold of either ipsilateral or contralateral hindpaws when comparing mice without plantar incision infused with saline (naive + saline) and mice without plantar incision treated with remifentanil (naive + remi) (Supplemental Figure 1, A–C; supplemental material available online with this article; https://doi.org/10.1172/JCI158742DS1). Note that we detected no differences among the indicated mouse groups in thermal pain thresholds of either the ipsilateral or contralateral hindpaws upon Hargreaves tests (Figure 1, D and E and Supplemental Figure 1, D and E).

Further, video observation of spontaneous paw lifting, licking, flinching, and guarding behaviors (26–28) showed no spontaneous pain behaviors in either naive + saline or naive + remi mice (Supplemental Figure 1, F and G). By contrast, both inci + saline mice and inci + remi mice exhibited markedly higher postoperative spontaneous pain scores in ipsilateral hindpaws after surgery (Figure 1F), but no significant differences in ipsilateral and contralateral hindpaws were observed between the 2 groups (Figure 1, F and G). Therefore, it appears likely that incisional mice focused more on the ipsilateral hindpaws — which had severe pain from surgery trauma — than they did on the contralateral hindpaws.

In addition to examining sensory pain, a real time–place escape/avoidance (RT-PEAP) test was used to assess emotional pain behaviors (28, 29) (Figure 1H). We found that when subthreshold mechanical noxious stimuli (0.07 g) were applied to the contralateral hindpaws, inci + remi mice exhibited stronger avoidance behaviors compared with inci + saline mice (Figure 1, I–K). These results suggest that intraoperative remifentanil administration may also cause emotional pain in response to noxious stimuli in RIH model mice.

A complete Freund’s adjuvant–induced (CFA-induced) inflammatory pain mouse model was then used to validate the effects of remifentanil on pain sensitization (Supplemental Figure 2, A–E). Compared with that in CFA mice administered with saline, remifentanil administration did not lead to any further decrease in mechanical or thermal pain thresholds of the ipsilateral hindpaws (Supplemental Figure 2, F, G, and I), while the mechanical — not thermal — pain thresholds of the contralateral hindpaws significantly decreased (Supplemental Figure 2, H and J). These results suggest that intraoperative remifentanil administration also induced postoperative secondary mechanical hyperalgesia in mice with CFA-induced inflammatory pain.

These collective results show that remifentanil combined with surgical incision could induce long-term secondary mechanical pain sensitization, while remifentanil treatment alone did not. Since the mechanical sensitivity induced by ipsilateral hindpaw incision was apparently too low to distinguish in mice, we focused on RIH in the contralateral hindpaws in subsequent experiments.

### RIH was accompanied by enhanced VPL^Glu^ neuronal activity.

The VPL has been proposed to function as a relay center in ascending pain pathways (9). To confirm the involvement of VPL neuronal activity in RIH development, we examined c-Fos expression in the brain on postoperative day 1. Immunofluorescence staining showed no detectable differences in c-Fos expression in bilateral VPL neurons between the naive + saline and naive + remi mice (Supplemental Figure 3, A–C). By contrast, the induction of RIH was accompanied by markedly elevated c-Fos expression in the ipsilateral VPL in inci + remi mice compared with that of inci + saline mice (Supplemental Figure 3, D and E), with approximately 90% of the c-Fos signal colocalized with glutamate immunofluorescence, and approximately 65% of glutamatergic neurons colocalized with c-Fos expression (Supplemental Figure 3, F and G). It is well known that nociceptive information evoked at the periphery is sent to the spinal cord on the ipsilateral side, and transmission to the brain continues in a contralateral crossover manner. Specifically, the pain signals originating in the ipsilateral hindpaws with incision are processed by the contralateral thalamus, and vice versa. Therefore, our results indicate that the ipsilateral VPL^Glu^ neurons, responsible for the perception of pain in the contralateral hindpaws, are activated in RIH.

**Figure 2:**
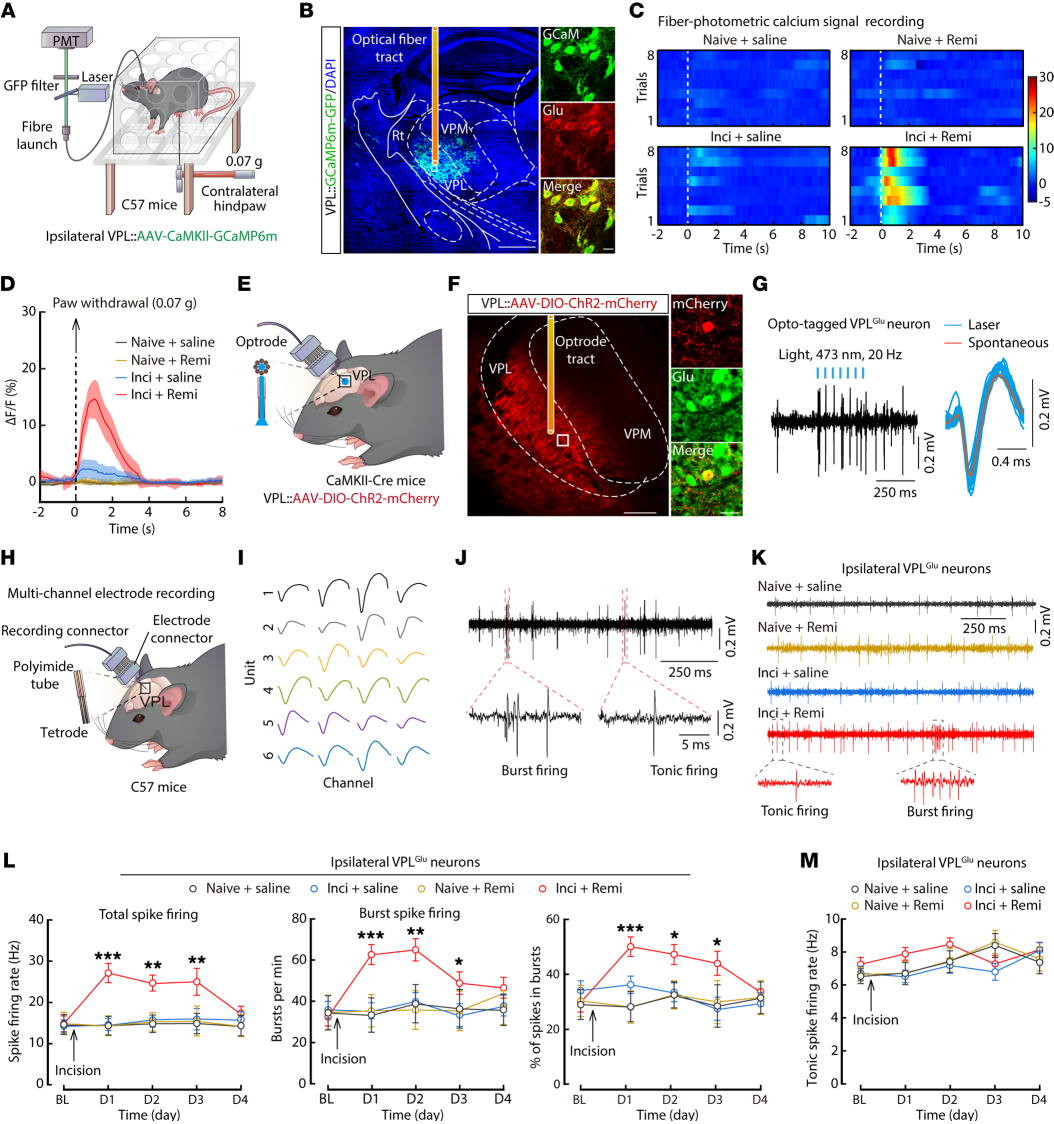
Enhanced ipsilateral VPL^Glu^ neuronal activity in the mouse model of RIH. (**A**) Schematic diagram of the fiber photometry setup. (**B**) Representative images validating the virus injection site (left) and GCaMP6m^+^ neurons costained with glutamate immunofluorescence (right) within the ipsilateral VPL. Scale bars: 1 mm (left) and 20 μm (right). (**C** and **D**) Heatmaps (**C**) and the mean data (**D**) showing VPL-Glu^GCaMP6m^ signals in mice. Color scale at the right in (**C**) indicates ΔF/F (%). (**E**) Schematic diagram of optogenetic tagging and electrophysiological recording. Enlargement showing optrodes. (**F**) Representative images of virus injection site of the VPL (left) and Cherry^+^ neurons colocalized with glutamate immunofluorescence (right). Scale bars: 200 μm (left) and 20 μm (right). (**G**) Example recording of spontaneous and light-evoked spikes from a VPL^Glu^ neuron (left) and overlay of averaged spontaneous (red) and light-evoked (blue) spike waveforms from the example unit (right). (**H**) Schematic of the multi-channel recording. Enlargement showing the multichannel tetrode. (**I**) Average spike waveform of widespiking putative VPL^Glu^ neurons recorded through a single tetrode. (**J**) Representative traces recorded from a VPL^Glu^ neuron showing the spontaneous burst and tonic firing. (**K**) Example traces of the spike firing recorded from ipsilateral VPL^Glu^ neurons. Tonic and burst firing are highlighted by dotted frames. (**L** and **M**) Quantitative data of total spike firing rate (left, *F*_(3,445.018)_ = 5.306, *P* = 0.002), burst number/min (middle, *F*_(3,459.614)_ = 6.161, *P* < 0.0001), percentage of spikes in bursts (right, *F*_(3,418.152)_ = 15.572, *P* = 0.005), and tonic spike firing rate (**M**, *F*_(3,527.508)_ = 1.086, *P* = 0.354) of ipsilateral VPL^Glu^ neurons (*n* = 37–91 neurons from 8 mice per group). Data are presented as mean ± SEM. *P < 0.05, **P < 0.01, ***P < 0.001. Linear mixed models with post hoc Bonferroni’s test in (**L** and **M**).

To directly test whether ipsilateral VPL^Glu^ neurons were sensitized to subthreshold mechanical stimuli under RIH, we infused the ipsilateral VPL of C57 mice with virally expressed fluorescent Ca^2+^ indicator GCaMP6m (AAV-CaMKII-GCaMP6m), then conducted calcium signal measurements in VPL^Glu^ neurons using optical fiber photometer recording (Figure 2, A and B). The results showed that calcium signals rapidly increased following stimulation with 0.07 g von Frey filament on the contralateral hindpaws of the inci + remi mice on postoperative day 1 (Figure 2, C and D and Supplemental Video 1), which indicated that VPL^Glu^ neurons participated in RIH-associated heightened sensitivity.

To explore the role of VPL^Glu^ neurons in the processing of pain, we used in vivo multi-channel electrode recordings in freely moving mice. We first identified the characteristics of spike waveforms evoked by laser stimulation (473 nm, 20 Hz) of VPL^Glu^ neurons using optogenetics in *CaMKII-Cre* mice (Figure 2, E–G and Supplemental Figure 4) and measured the 2 spiking patterns (tonic or burst firing) of VPL^Glu^ neurons under in vivo multi-channel electrode recordings (Figure 2, H–J). Under the 0.6 g von Frey filament stimulation of mouse hindpaws (Supplemental Figure 5, A and B), the total firing rate, burst firing rate, and the percentage of spikes in bursts recorded from contralateral VPL^Glu^ neurons in normal mice were both increased, while the tonic firing rate was not changed (Supplemental Figure 5, C–G); this indicated that VPL^Glu^ neurons were involved in nociceptive information processing and that the firing pattern of VPL^Glu^ neurons may be altered during pain sensing. Interestingly, we found that the spontaneous total firing rate, burst firing rate, and the percentage of spikes in bursts were all increased in the ipsilateral VPL^Glu^ neurons of inci + remi mice compared with those of inci + saline mice (Figure 2, K and L and Supplemental Figure 6, A–D). Notably, the tonic firing rate was not significantly different between inci + saline and inci + remi treated mice (Figure 2M, and Supplemental Figure 6E), suggesting that the increase in total firing was mainly due to elevated burst firing. Taken together, these data suggest that VPL^Glu^ neuronal activity was increased in mice with RIH, potentially due to enhanced burst firing.

### Ipsilateral VPL^Glu^ neurons of RIH mice exhibited increased burst firing.

**Figure 3:**
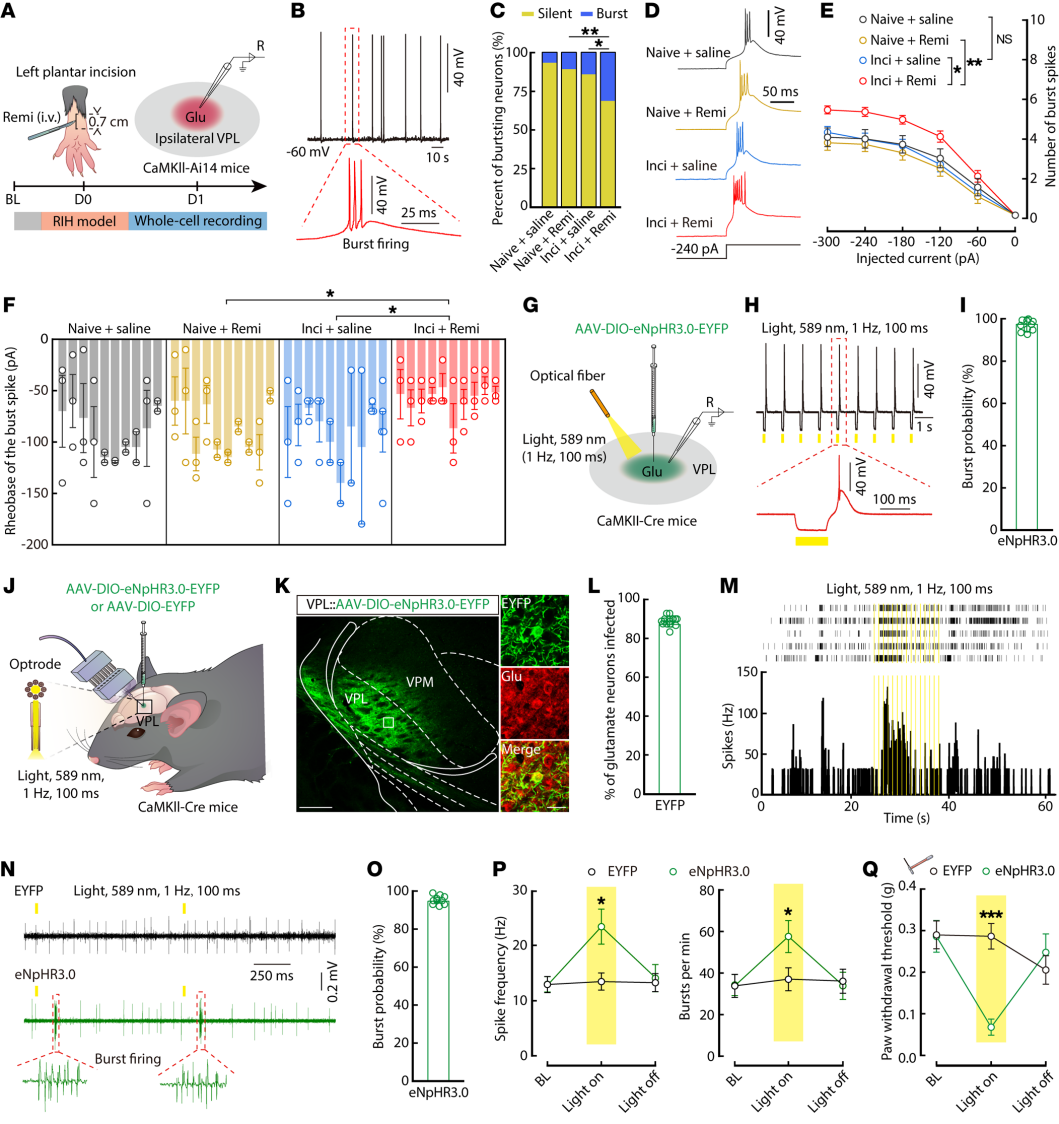
Hyperactivity of burst firing in ipsilateral VPL^Glu^ neurons causes postoperative RIH. (**A**) Schematic diagram of whole-cell recordings. (**B**) Spontaneous burst firing from an ipsilateral VPL^Glu^ neuron. (**C**) The percent of spontaneous burst firing in ipsilateral VPL^Glu^ neurons (*n* = 60–65 neurons from 6 mice per group; *P* = 0.0011, κ^2^ test). (**D**–**F**) Representative traces (**D**) and quantitative data of the number (**E**, *F*_(3,529.867)_ = 7.332, *P* < 0.0001) and rheobase (**F**, *F*_(3,104)_ = 4.722, *P* = 0.005) from 10 mice per group). (**G**) Virus injection and recording configuration in brain slices. (**H** and **I**) Representative traces (**H**) and the percentage (**I**) of burst firing induced by yellow light (n = 10 neurons from 5 mice). (**J**) Schematic of multi-channel recordings. (**K**) Viral expression within the VPL. Scale bars, 200 μm (left) and 20 μm (right). (**L**) The percentage of eNpHR3.0-EYFP-labeled neurons coexpressing with glutamate immunofluorescence (n = 16 slices from 8 mice). (**M**) Raster plot (top) and the histogram (bottom) showing the firing rate of VPL^Glu^ neurons. (**N**) Multi-channel recordings of spike firings from VPL^Glu^ neurons. (**O**) The percentage of bursts induced by yellow light (*n* = 10 neurons from 5 mice). (**P**) Quantitative data of spike frequency (left, *F*_(1,280.105)_ = 14.96, *P* = 0.048) and burst number/min (right, *F*_(1,286.984)_ = 5.312, *P* = 0.022) of VPL^Glu^ neurons (*n* = 50 neurons from 8 mice per group). (**Q**) Summary of pain thresholds in mice (*n* = 8 mice per group; *F*_(2,32)_ = 9.602, *P* = 0.0005).Data are presented as mean ± SEM. *P < 0.05, **P < 0.01, ***P < 0.001. Linear mixed models with post hoc Bonferroni’s test in **E** and **P**; nested 1-way ANOVA test in **F**; 2-way repeated measures ANOVA with post hoc Bonferroni’s test in **Q**.

To further investigate the relationship between altered burst firing in ipsilateral VPL^Glu^ neurons and RIH, we performed whole-cell recordings of glutamatergic neurons in VPL coronal slices of mice (Figure 3A). We found that the spontaneous burst firing (*I*_hold_ = 0 pA) was relatively rare in the naive group (6.66% in naive + saline mice, *n* = 4/60 neurons; 10.25% in naive + remi mice, *n* = 7/65 neurons) and could only be recorded in the VPL^Glu^ neurons with a resting membrane potential (RMP) near –60 mV (Figure 3B). By contrast, 27.78% (*n* = 19/61 neurons) of the recorded ipsilateral VPL^Glu^ neurons from inci + remi mice displayed enhanced firing compared with only 14.2% (*n* = 9/64 neurons) in inci + saline mice (Figure 3C). Previous studies have shown that the intrinsic membrane properties of VPL^Glu^ neurons are directly related to their burst firing (3, 20, 30). We therefore delivered a series hyperpolarized currents (from 0 pA to –300 pA, –10 pA/step, 500 ms) to these neurons and found that the action potentials of the burst appeared at the termination of hyperpolarized pulses (Figure 3D). Furthermore, we observed an increase in the number and a decrease in the rheobase of the burst firing in ipsilateral VPL^Glu^ neurons from inci + remi mice compared with those from inci + saline or naive + remi mice (Figure 3, E and F). In addition, we found that the RMP exhibited a higher degree of depolarization and that the input resistance (R_in_) decreased to a greater extent in ipsilateral VPL^Glu^ neurons of inci + remi mice compared with those of the inci + saline or naive + remi mice (Supplemental Figure 6, F-H). These results suggested that alterations in the intrinsic membrane properties of VPL^Glu^ neurons may lead to enhanced burst firing in RIH mice.

In order to test whether enhanced burst firing is sufficient to induce pain sensitization in mice, we infected VPL^Glu^ neurons with a *Cre-*dependent variant of eNpHR3.0 virus (AAV-DIO-eNpHR3.0-EGFP) and used optogenetic tools to induce burst firing in the *CaMKII-Cre* mice (30, 31) (Figure 3G). Examination of brain slices revealed that burst firing could be successfully evoked at the termination of the yellow light pulse stimulations (589 nm, 1 Hz, pulse width 100 ms) in VPL^Glu^ neurons expressing eNpHR3.0 (*I*_hold_ = 0 pA) (Figure 3, H and I). These results were confirmed by in vivo optrode recordings in freely moving mice (Figure 3, J–L), which further showed that burst firing robustly increased with a success rate of over 90% during yellow light photostimulation (Figure 3, M-O). Statistical analysis indicated that spike frequency and burst number/min significantly increased during photostimulation (Figure 3P). In addition, behavioral tests showed that the typical pattern of yellow light photostimulation in the VPL^Glu^ neurons could acutely drive allodynia in mice (Figure 3Q). In light of these findings, we hypothesized that increased burst firing of VPL^Glu^ neurons serves as major etiological basis for RIH in mice.

### Upregulation of T-type calcium channels of VPL^Glu^ neurons in mice with RIH.

Previous studies have shown that low-threshold-activated T-type Ca_v_3.1 channels are the predominant subtype in thalamocortical projection neurons and play a key role in initiating burst firing (22, 24, 32, 33). To better understand their contribution to RIH, we performed immunofluorescence staining of Ca_v_3.1 channels and found that they were highly expressed in VPL^Glu^ neurons in mice (Supplemental Figure 7A).

**Figure 4:**
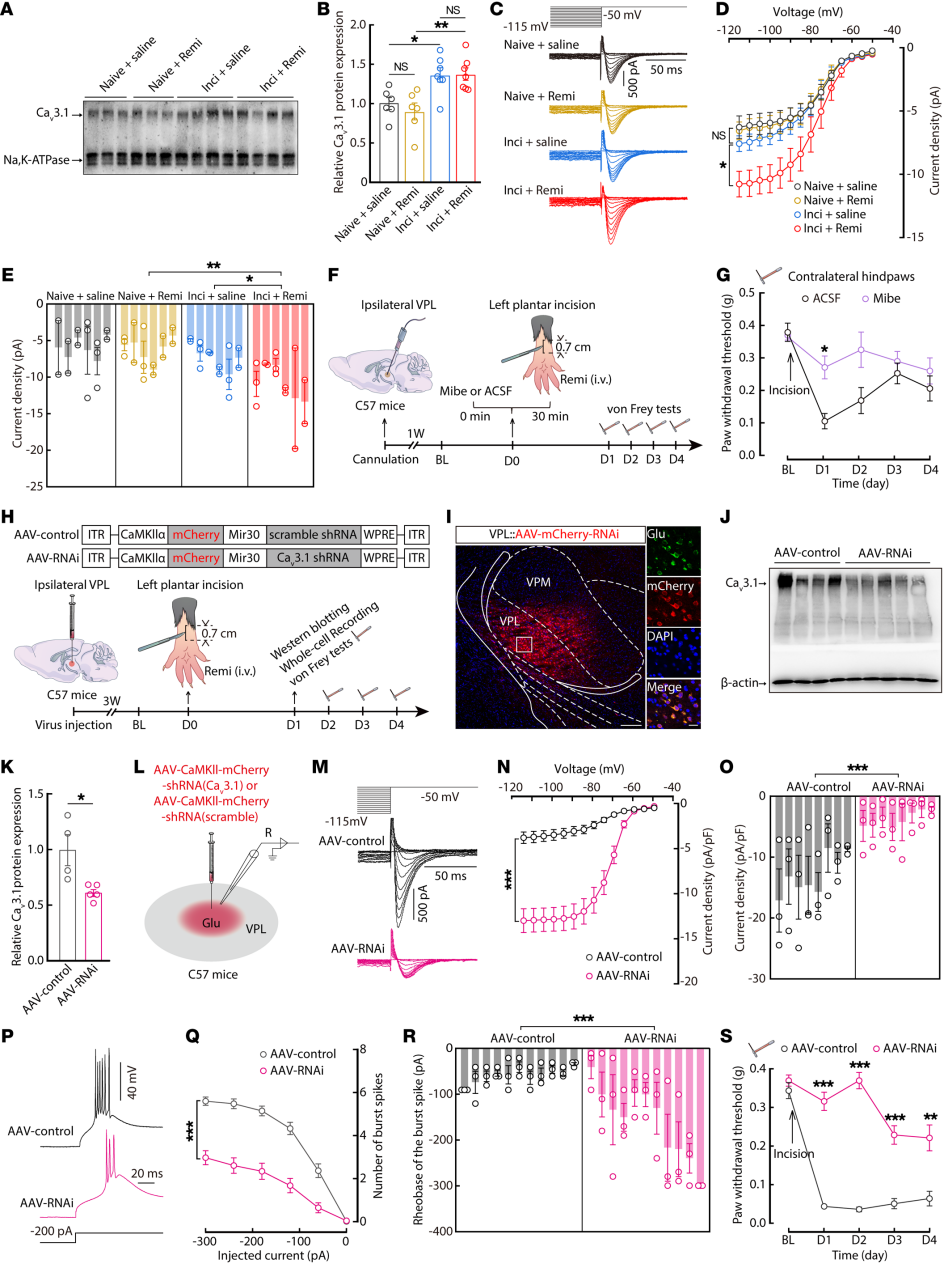
Downregulation of T-type calcium channels blocks burst firing and relieves postoperative RIH. (**A** and **B**) Ca_v_3.1 protein level in cell membrane fractions from ipsilateral VPL tissue (**B**, *n* = 6–7 mice per group; *F*_(3,22)_ = 7.633, *P* = 0.0011). (**C**) Representative traces of T-type calcium currents. (**D** and **E**) Current-voltage *(I-V*) curves (**D**, *F*_(3,569.088)_ = 46.526, *P* < 0.0001) and quantitative data (**E**, *F*_(3,52)_ = 6.694, *P* = 0.0007; at −115 mV) of current density (*n* = 14 neurons from 6 mice per group). (**F**) Schematic of the experimental procedure. (**G**) The effect of microinjection of Mibe on the postoperative hyperalgesia (*n* = 8 mice per group; *F*_(1,14)_ = 14.34, *P* = 0.002). (**H**) Schematic for virus injection. (**I**) Virus expression within the VPL. Scale bars: 200 μm (left) and 20 μm (right). (**J** and **K**) Ca_v_3.1 expression in ipsilateral VPL lysates (AAV-control, *n* = 4 mice; AAV-RNAi, *n* = 5 mice; *t*_(7)_ = 3.08, *P* = 0.0178). (**L**) Schematic of virus injection and recording configuration. (**M**) Representative traces of T-type calcium currents. (**N** and **O**) Current-voltage (*I-V*) curves (**N**, *F*_(1,622.864)_ = 267.89, *P* < 0.0001) and quantitative data (**O**, *t*_(46)_ = 6.265, *P* < 0.0001; at −115 mV) of current density (*n* = 24 neurons from 8 mice per group). (**P**–**R**) Representative traces (**P**) and quantitative data of the number (**Q**, *F*_(1,333)_ = 53.601, *P* < 0.0001) and rheobase (**R**, *t*_(20)_ = 4.215, *P* = 0.0004) of the burst spike (*n* = 33 neurons from 11 mice per group). (**S**) Quantitative data for mechanical thresholds (AAV-control, *n* = 10 mice; AAV-RNAi, *n* = 8 mice; *F*_(1,16)_ = 148.2, *P* < 0.0001). Data are presented as mean ± SEM. *P < 0.05, **P < 0.01, ***P < 0.001. 1-way ANOVA with post hoc Bonferroni’s test in (**B**); linear mixed models with post hoc Bonferroni’s test in (**D**, **N** and **Q**); nested 1-way ANOVA test in (**E**); 2-way repeated measures ANOVA with post hoc Bonferroni’s test in (**G** and **S**); unpaired 2-tailed Student’s *t* test in (**K**); and nested 2-tailed *t* test in (**O** and **R**).

We next investigated the molecular mechanisms underlying the increased burst firing in the RIH mouse model. After validating the specificity of the antibody for Ca_v_3.1 (#PA5-77311, Thermo Fisher Scientific) (Figure 4, H–K), Western blots showed that global Ca_v_3.1 protein levels from ipsilateral VPL lysates were upregulated in inci + remi and inci + saline mice, relative to those of the naive + remi and naive + saline mice, respectively (Supplemental Figure 7, D and E). Further, we also examined the Ca_v_3.1 protein levels in the cell membrane fractions extracted from ipsilateral VPL tissues of each group (Supplemental Figure 7, B and C). Compared with the naive mice, Ca_v_3.1 protein levels were significantly higher in the inci + saline and inci + remi mice, although no significant difference was detected between these 2 groups (Figure 4, A and B), suggesting that Ca_v_3.1 channel function may change during RIH.

We then used electrophysiological recordings in whole-cell voltage-clamp mode, as described previously (20, 21, 30, 34), to specifically isolate T-type calcium channel-mediated currents by holding membrane voltage at –60 mV with 500-ms-long voltage steps of –115 mV through –50 mV (Figure 4C and Supplemental Figure 8A). These currents could also be eliminated by bath application of the T-type calcium channel–specific blocker mibefradil (15 μM) (Supplemental Figure 8, A and B). Comparison between treatment groups showed that the current density for T-type calcium channels was higher in the ipsilateral VPL^Glu^ neurons of inci + remi mice, compared with that of inci + saline mice (Figure 4, C–E), indicating that T-type calcium channel function was enhanced in the presence of remifentanil.

Behavioral tests were performed to examine whether blocking T-type calcium channels had any effect on hyperalgesia in RIH mice. To this end, mibefradil was microinjected into the ipsilateral VPL 30 min before surgical incision in inci + remi mice, and the mechanical pain threshold was measured over the next 4 days (Figure 4F). We found that pretreatment with mibefradil could effectively prevent hypersensitivity of contralateral hindpaws and suppress the burst firing in VPL^Glu^ neurons compared with RIH mice given artificial cerebrospinal fluid (ACSF) control pretreatments (Figure 4G, and Supplemental Figure 8, C–F). Similar behavioral effects were obtained by ipsilateral VPL postadministration of mibefradil in RIH mice (Supplemental Figure 8, G and H). Moreover, in naive *CaMKII-Cre* mice with VPL^Glu^ neurons expressing eNpHR3.0 (Supplemental Figure 8I), we found that mibefradil pretreatment for 30 minutes reversed yellow light-induced (589 nm, 1 Hz, pulse width 100 ms) mechanical allodynia behavior (Supplemental Figure 8J), but not in mice infected with control virus (Supplemental Figure 8K). These data suggest that the hyperactive burst firing observed in RIH mice was mainly caused by dysfunction of T-type calcium channels.

We next designed an RNAi viral vector (AAV-CaMKII-mCherry-shRNA-(Ca_v_3.1)) to knock down Ca_v_3.1 channels in VPL^Glu^ neurons (Figure 4, H and I). For RIH mice, at 3 weeks after VPL injection of Ca_v_3.1 RNAi virus (AAV-RNAi), Western blots showed that Ca_v_3.1 protein levels in the VPL were reduced to approximately 61.5% of that in the AAV-CaMKII-mCherry-shRNA–infected (scramble, AAV-control-infected) group (Figure 4, J and K). Compared with the AAV-control group, T-type calcium channel-mediated currents were decreased in AAV-RNAi-expressing ipsilateral VPL^Glu^ neurons (Figure 4, L–O), accompanied by decreased spike number and increased rheobase of burst firing (Figure 4, P–R). In addition, knockdown of Ca_v_3.1 channels in ipsilateral VPL^Glu^ neurons by AAV-RNAi reduced hypersensitivity in the contralateral hindpaws of RIH mice (Figure 4S). In naive mice infected with AAV-RNAi (Supplemental Figure 9, A and B), the yellow light-induced burst firing in VPL^Glu^ neurons (Supplemental Figure 9, C–E) and mechanical allodynia behavior (Supplemental Figure 9F) both disappeared. These findings indicated that burst firing in VPL^Glu^ neurons, as regulated by Ca_v_3.1, contributes to RIH development.

We also tested whether sufentanil (sufen) — another μ-opioid receptor agonist widely used in the clinic (35, 36) — affected pain sensation in both incisional and CFA mice (Supplemental Figure 10, A and F). Interestingly, no postoperative hyperalgesia was observed in either incisional or CFA mice treated with sufentanil (0.5 μg/kg, i.v., 0.017 μg/kg/min) (Supplemental Figure 10, B–J). In addition, immunofluorescence staining showed no difference between inci + saline and inci + sufen mice in c-Fos expression (Supplemental Figure 11, A and B) nor in the percentage of glutamatergic neurons that colocalized with c-Fos^+^ neurons (Supplemental Figure 11, C and D) in the ipsilateral VPL. Furthermore, in vivo multi-channel electrode recordings showed no differences in total firing rate or burst firing rate of ipsilateral VPL^Glu^ neurons between inci + sufen and inci + saline mice (Supplemental Figure 11, E and F). These results were consistent with the results of optical fiber photometer recording of calcium activities in the ipsilateral VPL^Glu^ neurons of both groups (Supplemental Figure 11, G and H).

Examination of the contralateral VPL showed that both inci + saline and inci + sufen groups exhibited high expression of c-Fos^+^ neurons colabeled with glutamate antibody (Supplemental Figure 11, I–L) and high firing rates of spontaneous tonic and burst firings in VPL^Glu^ neurons, with no significant differences detected between the 2 groups (Supplemental Figure 11, M and N). These results indicate that sufentanil did not cause postoperative hyperalgesia and did not appear to affect the activity of VPL^Glu^ neurons. Taken together, our results suggest that differences between remifentanil and sufentanil treatment can provide insight into the mechanism by which remifentanil induces hyperalgesia.

### Burst firing in VPL^Glu^ neurons regulated activity of the VPL^Glu^→S1HL^Glu^ circuit.

**Figure 5:**
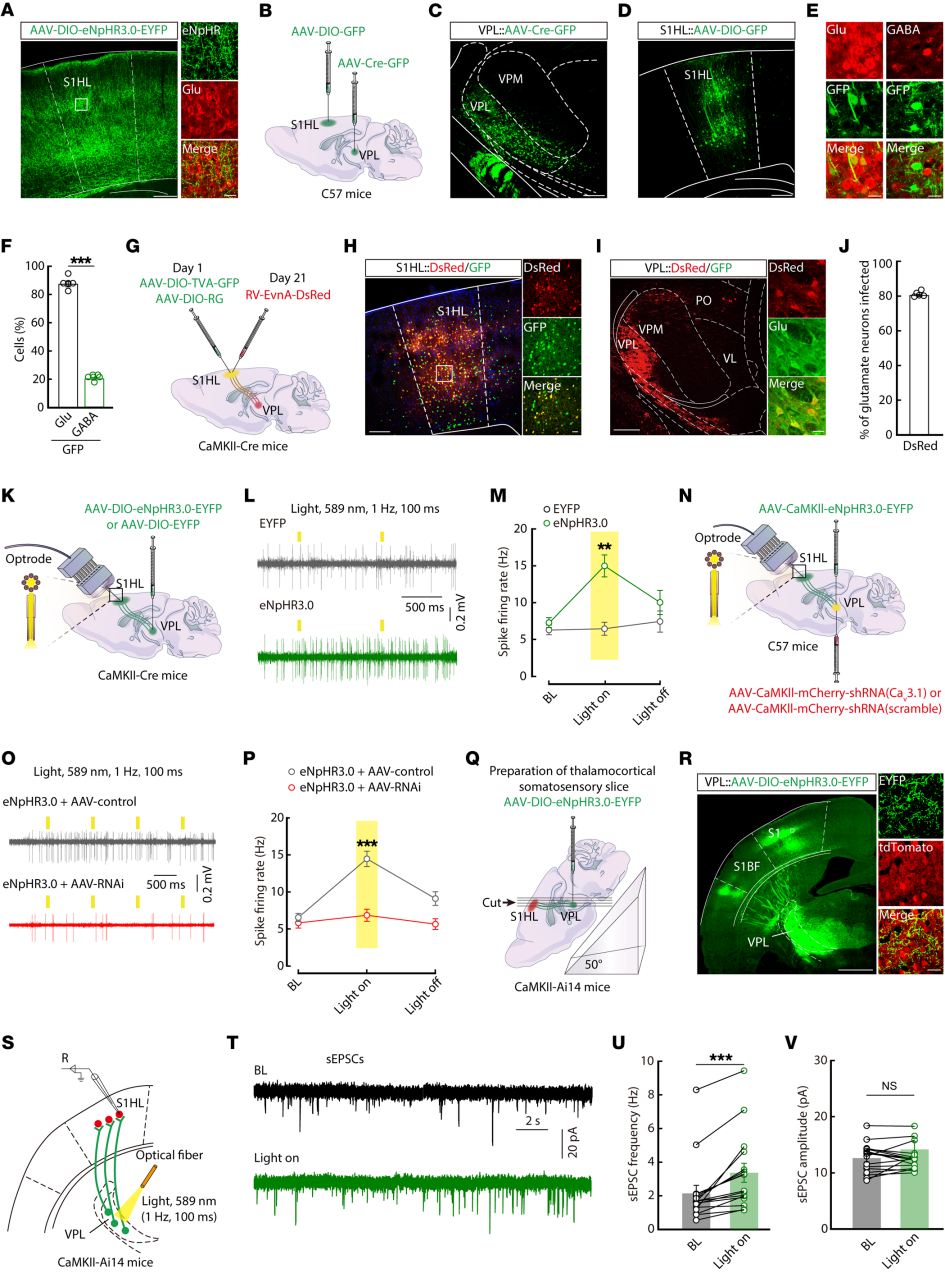
Dissection of the VPL^Glu^→S1HL^Glu^ circuit. (**A**) Representative images of EYFP^+^ fibers in the S1HL. Scale bars: 200 μm (left) and 20 μm (right). (**B**) Schematic of strategy for virus injection. (**C** and **D**) Viral expression within the VPL (**C**) and S1HL (**D**). Scale bars: 200 μm. (**E** and **F**) Colocalization of GFP^+^ and glutamate^+^ signals within the S1HL (**F**, *n* = 5 slices from 5 mice per group; *t*_(8)_ = 32.36, *P* < 0.0001) showing. Scale bars: 20 μm in (**E**). (**G**) Schematic of the virus tracing strategy. (**H**) The injected site (left) and viral expression (right) within the S1HL. Scale bars: 200 μm (left) and 20 μm (right). (**I** and **J**) Colocalization of DsRed-labeled neurons and glutamate^+^ signals within the VPL (**J**, *n* = 5 slices from 5 mice). Scale bars: 200 μm (left) and 20 μm (right) in (**I**). (**K**) Schematic of the optrode implantation in the S1HL and virus infusion. (**L** and **M**) Representative traces (**L**) and summarized data (**M**, *n* = 13 neurons from 6 mice per group; *F*_(1,74)_ = 22.81, *P* < 0.0001) of the firing rate of the S1HL^Glu^ neurons. (**N**) Schematic of of the experimental procedure. (**O** and **P**) Example traces (**O**) and quantitative data (**P**) of spike firing in S1HL^Glu^ neurons (*n* = 25–32 neurons from 8 mice per group; *F*_(1,190.74)_ = 26.171, *P* < 0.0001). (**Q**) Schematic for the preparation of thalamocortical somatosensory slices. (**R**) EYFP^+^ fibers in thalamocortical somatosensory slices. Scale bars: 1 mm (left) and 20 μm (right). (**S**) Schematic for the light stimulation within the VPL and recording configuration. (**T**–**V**) Representative traces and quantitative data of the frequency (**U**, *t*_(15)_ = 4.775, *P* = 0.0002) and amplitude (**V**, *t*_(15)_ = 0.2537, *P* = 0.8212) of sEPSCs recorded from S1HL^Glu^ neurons. Data are presented as mean ± SEM. **P < 0.01, ***P < 0.001. Unpaired 2-tailed Student’s *t* test in **F**; linear mixed models with post hoc Bonferroni’s test in **M** and **P**; paired 2-tailed *t* test in **U** and **V**.

The most distinctive feature of the thalamus is its interconnection with the cerebral cortex (8), which serves as a higher-order relay for integrating and differentiating diverse pain signals (3, 37). In order to identify the pain-associated thalamocortical circuits, we first dissected the functional connectivity of this distinct pathway from the VPL to the cortex. For this purpose, we first infused an AAV-DIO-eNpHR3.0-EYFP reporter virus into the VPL of *CaMKII-Cre* mice, as shown in Figure 3K. Three weeks later, we observed abundant EYFP^+^ fibers in the S1HL (Figure 5A), which is well known to participate in processing hindlimb pain (38). To confirm this VPL→S1HL projection, we injected anterograde monosynaptic AAV-Cre-GFP virus into the VPL, and AAV-DIO-GFP virus into the S1HL of C57 mice (Figure 5, B and C). 3 weeks later, GFP^+^ neurons were observed in the S1HL (Figure 5D) and prominently colocalized with immunofluorescence signals from the glutamate antibody, but not from the GABA antibody (Figure 5, E and F).

To characterize the VPL→S1HL organization, we used a cell-type-specific retrograde trans-monosynaptic tracing system (Figure 5G). Cre-dependent helper viruses (AAV-EF1α-DIO-TVA-GFP and AAV-EF1α-DIO-RVG) were injected into the S1HL of *CaMKII-Cre* mice, and after 3 weeks, a rabies virus–based (RV-based) reporter (EnvA-pseudotyped RV-ΔG-DsRed) was injected into the same site (Figure 5H). We identified intensely DsRed^+^ neurons in the zona incerta, the contralateral S1, the secondary somatosensory cortex, and the posterior thalamic nucleus (Supplemental Figure 12, A and B). Importantly, we also found intensely DsRed^+^ neurons in the VPL, suggesting that VPL neurons innervate S1HL^Glu^ neurons (Figure 5I and Supplemental Figure 12C). In addition, the DsRed^+^ neurons in the VPL primarily colocalized with antiglutamate signals (Figure 5J) and antibody targeting Ca_v_3.1 channels (Supplemental Figure 12D). These findings thus revealed a VPL^Glu^→S1HL^Glu^ circuit.

To determine whether the burst firing of VPL^Glu^ neurons is sufficient to alter the activity of the VPL^Glu^→S1HL^Glu^ circuit, we examined the neuronal activity of S1HL^Glu^ neurons by c-Fos staining (Supplemental Figure 13A) and in vivo optrode recordings (Figure 5K) following yellow light photostimulation (589 nm, 1 Hz, pulse width 100 ms) of VPL^Glu^ neurons in naive mice. Notably, c-Fos expression was increased in S1HL^Glu^ neurons after photostimulation of eNpHR3.0-expressing VPL^Glu^ neurons (Supplemental Figure 13, B and C), in which 88.8% of glutamatergic neurons colocalized with c-Fos signals (Supplemental Figure 13, D and E). In addition, after the photostimulation of eNpHR3.0-expressing VPL^Glu^ neuronal fibers in the S1HL by implanted optrodes, in vivo recordings showed a significant increase in the firing rates of S1HL^Glu^ neurons (Figure 5, L and M), which were abolished by knockdown of Ca_v_3.1 in VPL^Glu^ neurons (Figure 5, N–P).

To examine the synaptic mechanism by which burst firing of VPL^Glu^ neurons contributes to the regulation of VPL^Glu^→S1HL^Glu^ circuit activity, we combined in vitro electrophysiological recordings with optogenetic methods. Using whole-cell recordings in thalamocortical slices (Supplemental Figure 14, A–C), we observed that brief light stimulation of ChR2-containing VPL^Glu^ neurons with the optical fiber positioned above the VPL consistently elicited excitatory postsynaptic currents (EPSCs) in S1HL^Glu^ neurons (Supplemental Figure 14, D and E). These EPSCs were blocked by bath application of brain slices with tetrodotoxin (TTX, 1 μM), which could be rescued by the addition of the potassium channel blocker 4-aminopyridine (4-AP, 4 mM) to the TTX treatment. This rescue effect could then be eliminated by adding AMPA receptor antagonist 6,7-Dinitroquinoxaline-2,3(1H,4H)-dione (20 μM) to the TTX/4-AP cotreatment (Supplemental Figure 14, E and F). These results demonstrated that S1HL^Glu^ neurons received functional monosynaptic excitatory glutamatergic projections from VPL^Glu^ neurons. In addition, we found that yellow light stimulation of eNpHR3.0^+^ VPL^Glu^ neurons in thalamocortical slices (Figure 5, Q–S) resulted in an increase in the frequency, but not the amplitude, of spontaneous EPSCs (sEPSCs) in the S1HL^Glu^ neurons (Figure 5, T–V). Taken together, our findings suggest that the burst firing of VPL^Glu^ neurons can indeed alter the functional connectivity of the VPL^Glu^→S1HL^Glu^ circuit. We therefore proposed that this thalamocortical functional alteration could be mediated via a presynaptic regulatory mechanism that controls the efficiency of excitatory synaptic transmissions in S1HL^Glu^ neurons.

### Increased excitability of ipsilateral S1HL^Glu^ neurons in RIH mice.

**Figure 6:**
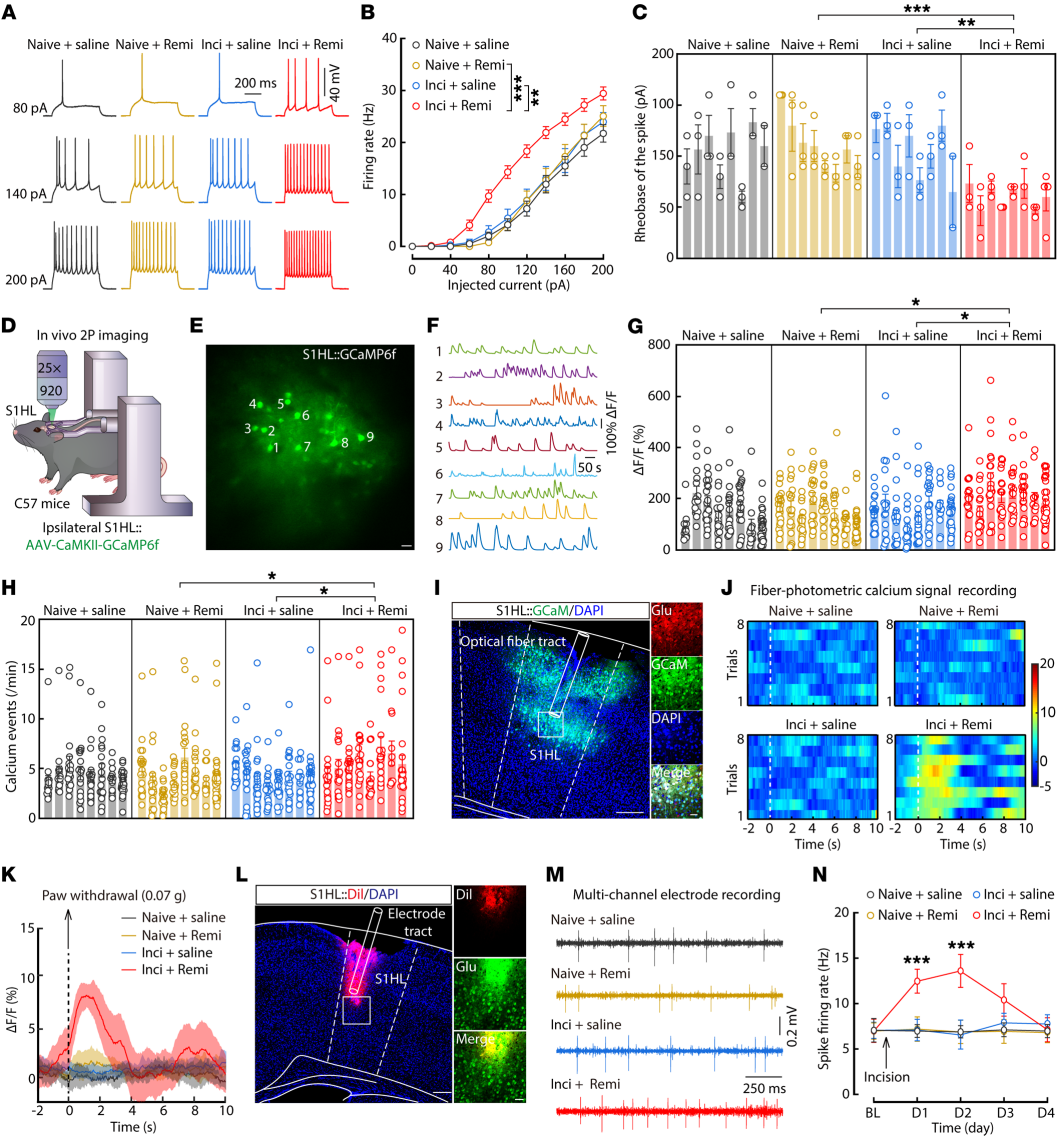
Increased ipsilateral S1HL^Glu^ neuronal excitability in mice with remifentanil induced allodynia. (**A**–**C**) Representative traces (**A**) and quantitative data of the firing rate (**B**, *F*_(3,956.954)_ = 18.748, *P* < 0.0001) and rheobase (**C**, *F*_(3,28)_ = 8.543, *P* = 0.0003) of action potentials recorded in ipsilateral S1HL^Glu^ neurons (*n* = 23-25 neurons from 8 mice per group). (**D**) Schematic paradigm of in vivo 2-photon (2P) calcium imaging in head-restrained C57 mice. (**E** and **F**) Representative images of 2P GCaMP6f^+^ imaging fields (**E**) and numbers matching spontaneous ΔF/F time series traces (**F**) of ipsilateral S1HL^Glu^ neurons. Scale bar: 20 μm. (**G** and **H**) Average of spontaneous calcium responses (**G**, *F*_(3,28)_ = 4.4, *P* = 0.0117) and calcium event rates (**H**, *F*_(3,28)_ = 4.505, *P* = 0.0106) in GCaMP6^+^ ipsilateral S1HL^Glu^ neurons (*n* = 154–159 neurons from 8 mice per group). (**I**) Representative images of GCaMP6m expression within the VPL (left) and colocalization of GCaMP6m^+^ neurons and glutamate immunofluorescence (right). Scale bars: 200 μm (left) and 50 μm (right). (**J** and **K**) Heatmaps (**J**) and the representative average ΔF/F traces (**K**) of S1HL Glu^GCaMP6m^ signals. (**L**) Representative images of the tetrode placement site in the S1HL. Scale bars: 200 μm (left) and 40 μm (right). (**M** and **N**) Example traces (**M**) and quantitative data (**N**, *n* = 22–48 neurons from 8 mice per group; *F*_(3,575.662)_ = 9.436, *P* < 0.0001) of spike firing of ipsilateral S1HL^Glu^ neurons. Dil, 1,1′-dioctadecyl-3,3,3′,3′-tetramethylindocarbocyanine perchlorate. Data are presented as mean ± SEM. *P < 0.05, **P < 0.01, ***P < 0.001. Linear mixed models with post hoc Bonferroni’s test in (**B** and **N**); nested 1-way ANOVA with post hoc Bonferroni’s test in (**C**, **G** and **H**).

In light of our above findings, we hypothesized that if S1HL^Glu^ neurons were innervated by VPL^Glu^ inputs in an excitatory state, then an increase in inputs under RIH conditions could induce excitatory effects. We detected no difference in c-Fos expression in ipsilateral S1HL^Glu^ neurons between naive + saline and naive + remi mice by immunofluorescence staining (Supplemental Figure 15, A–C). However, there was a notable increase in c-Fos expression in ipsilateral S1HL^Glu^ neurons of inci + remi mice compared with those of inci + saline *CaMKII-Ai14* mice on postoperative day 1 (Supplemental Figure 15, D–G). In addition, whole-cell recordings showed an increase in firing rate (Supplemental Figure 16, A and B and Figure 6, A and B) and a decrease in the rheobase (Figure 6C) of action potentials in S1HL^Glu^ neurons of inci + remi mice compared with those of inci + saline mice. Moreover, depolarization of RMPs was also observed (Supplemental Figure 16C) in ipsilateral S1HL^Glu^ neurons of inci + remi mice, while the R_in_ was unaffected (Supplemental Figure 16D).

In order to visualize calcium signals in conscious mice, AAV-CaMKII-GCaMP6f virus was injected into the ipsilateral S1HL of C57 mice (Figure 6D). Subsequent in vivo 2-photon calcium imaging showed that the fluorescence intensity and event of spontaneous calcium signals significantly increased in GCaMP6f^+^ S1HL^Glu^ neurons in inci + remi mice compared with that of inci + saline mice (Figure 6, E–H and Supplemental Figure 16, E and F). In addition, photometric calcium signal recordings showed enhanced calcium signals in the ipsilateral S1HL^Glu^ neurons upon 0.07 g von Frey stimuli applied to contralateral hindpaws in freely moving RIH mice (Figure 6, I–K and Supplemental Video 2). In vivo multi-tetrode recordings also showed that spontaneous firing increased in ipsilateral S1HL^Glu^ neurons (Figure 6, L–N and Supplemental Figure 17). These cumulative results showed that ipsilateral S1HL^Glu^ neurons were hyperactivated in RIH mice.

Examination of the contralateral S1HL in mice revealed that c-Fos expression and neuronal excitability were both increased in S1HL^Glu^ neurons of the inci + remi group compared with that of inci + saline *CaMKII-Ai14* mice on postoperative day 1 (Supplemental Figure 18, A–H). The elevated neuronal activity described above was also confirmed by in vivo recording of photometric calcium signal, 2-photon calcium imaging, and multi-tetrode recordings in mice (Supplemental Figure 19). In particular, calcium signals rapidly increased following stimulation with 0.07 g von Frey filament on the ipsilateral hindpaws in contralateral S1HL^Glu^ neurons of inci + remi and inci + saline mice, but not in those of naive + saline or naive + remi mice (Supplemental Figure 19, A–C). Interestingly, the in vivo 2-photon calcium imaging (Supplemental Figure 19, D–F) and multi-tetrode recordings (Supplemental Figure 19, G–I) showed that contralateral S1HL^Glu^ neuronal activity was significantly increased in the inci + remi mice compared with that in the inci + saline or naive + remi mice, indicating that surgery was a prerequisite for hyperalgesia, and that remifentanil mediates its development postoperatively.

In addition, we also evaluated the effects of sufentanil on the activity of S1HL^Glu^ neurons in mice. For ipsilateral S1HL^Glu^ neurons, c-Fos immunofluorescence staining showed no difference in neuronal activity between inci + sufen and inci + saline *CaMKII-Ai14* mice on postoperative day 1 (Supplemental Figure 20, A–D), which was in agreement with in vivo multi-tetrode (Supplemental Figure 20, E and F) and photometric calcium signal recordings (Supplemental Figure 20, G and H). For the contralateral S1HL^Glu^ neurons, although the c-Fos expression (Supplemental Figure 20, I–L) and firing rate of spontaneous spikes (Supplemental Figure 20, M and N) were both elevated, no obvious difference in these measurements was observed between the inci + sufen and inci + saline groups. These results collectively indicated that sufentanil itself does not alter the bilateral S1HL^Glu^ neuronal activities in mice given surgical plantar incisions.

### Essential role of the VPL^Glu^→S1HL^Glu^ pathway in RIH.

**Figure 7:**
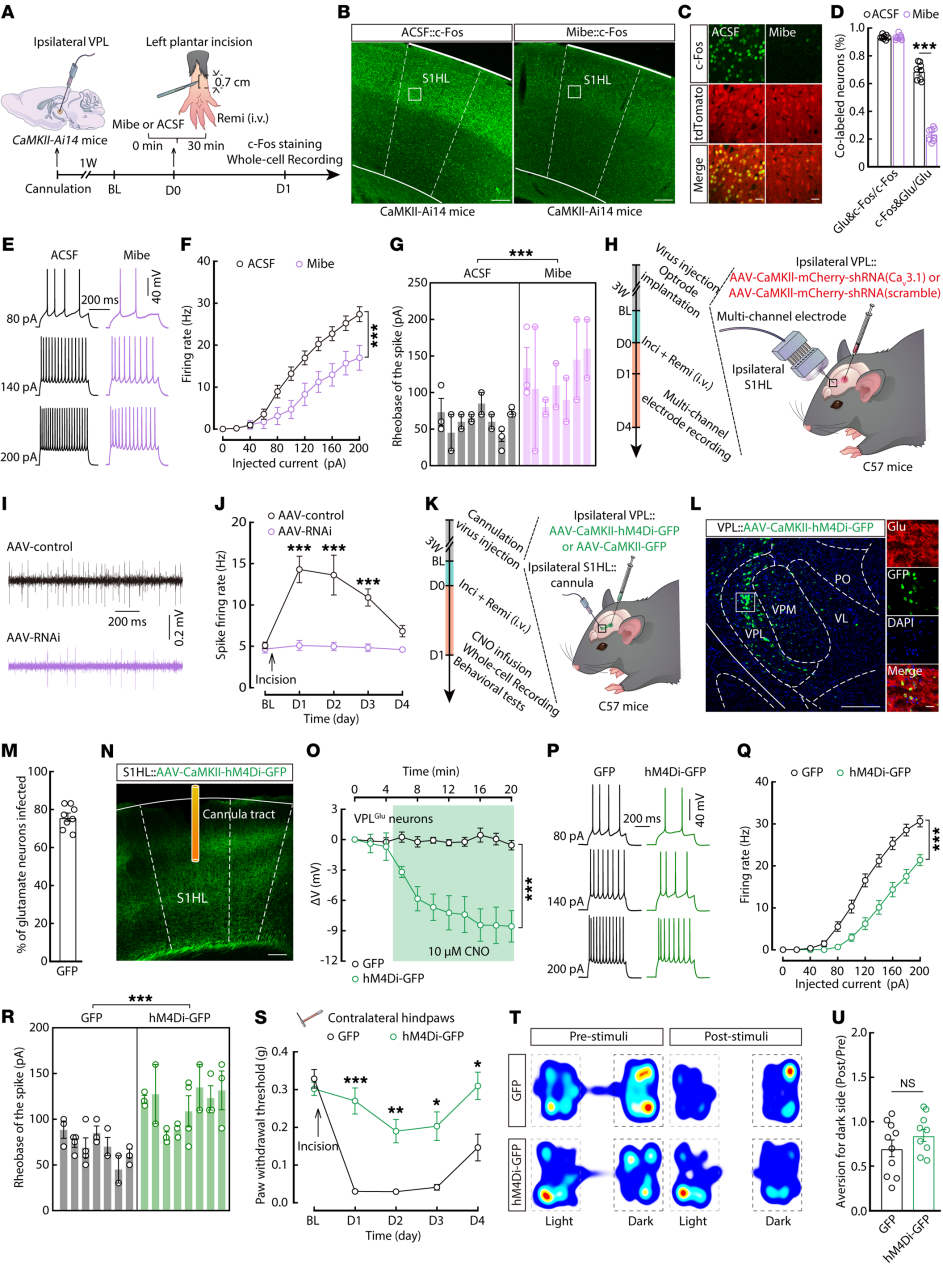
The VPL^Glu^ → **S1HL^Glu^ circuit controls allodynia in RIH mice.** (**A**) Schematic of the experimental procedure. (**B**) c-Fos expression in ipsilateral S1HL. Scale bars: 200 μm. (**C**) Colocalization of c-Fos^+^ neurons with tdTomato^+^. Scale bars: 20 μm. (**D**) The percentage of c-Fos^+^ and glutamate expression (left, *t*_(14)_ = 0.4611, *P* = 0.6518; right, *t*_(14)_ = 17.61, *P* < 0.0001) in the ipsilateral S1HL (*n* = 8 slices from 5 mice per group). (**E**–**G**) Representative traces (**E**) and quantitative data of the firing rate (**F**, *F*_(1,348.99)_ = 67.193, *P* < 0.0001) and rheobase (**G**, *t*_(33)_ = 4.207, *P* = 0.0002) of action potentials from ipsilateral S1HL^Glu^ neurons (*n* = 20 neurons from 8 ACSF mice; *n* = 15 neurons from 7 Mibe mice). (**H**) Virus injection and optrode implantation in RIH mice. (**I** and **J**) Example traces (**I**) and quantitative data (**J**) for spike firing (*n* = 25–49 neurons from 8 mice per group; *F*_(1,270.918)_ = 18.013, *P* = 0.0002). (**K**) Schematic of microinjection. (**L**) Viral expression within the VPL. Scale bars: 200 μm (left) and 20 μm (right). (**M**) The percentage of glutamate^+^ neurons expressing GFP signals (*n* = 8 slices from 5 mice). (**N**) GFP^+^ fibers in the S1HL. Scale bars: 200 μm. (**O**) Effects of CNO on VPL^Glu^ neurons (*n* = 5 neurons from 5 mice per group; *F*_(1,188)_ = 118.596, *P* < 0.0001). (**P**–**R**) Representative traces (**P**) and quantitative data of the firing rate (**Q**, *F*_(1,460.808)_ = 39.677, *P* < 0.0001) and rheobase (**R**, *t*_(13)_ = 4.954, *P* = 0.0003) of action potentials from ipsilateral S1HL^Glu^ neurons (*n* = 23 neurons from 7 GFP mice or 8 GFP-hM4Di mice).(**S**) Mechanical pain thresholds in RIH mice injected with CNO in the S1HL (*n* = 9 mice per group; *F*_(1,16)_ = 64.69, *P* < 0.0001). (**T** and **U**) Heatmaps (**T**) and summary data (**U**, GFP, *n* = 10 mice; hM4Di-GFP, *n* = 9 mice; *t*_(17)_ = 1.242, *P* = 0.231) for RT-PEAP tests of RIH mice. Data are presented as mean ± SEM. *P < 0.05, **P < 0.01, ***P < 0.001. Unpaired 2-tailed Student’s *t* test in **D** and **U**, linear mixed models with post hoc Bonferroni’s test in **F**, **J**, **O** and **Q**, nested2-tailed *t* test in **G** and **R**; 2-way repeated measures ANOVA with post hoc Bonferroni’s test in **S**.

In light of the increased excitatory inputs in the VPL^Glu^→S1HL^Glu^ circuit during RIH, we next examined S1HL^Glu^ neuronal activity after preoperative local inhibition of T-type calcium channels in the ipsilateral VPL by microinfusion of mibefradil. Immunohistochemistry staining and whole-cell recordings in brain slices showed that c-Fos expression and the neuronal excitability were both significantly decreased in Tdtomato^+^ ipsilateral S1HL^Glu^ neurons of *CaMKII-Ai14* RIH mice (see Supplemental Methods) pretreated with mibefradil compared with RIH mice pretreated with ACSF (Figure 7, A–G). It bears mention that posttreatment of mibefradil or knockdown of Ca_v_3.1 channels in the VPL of RIH mice also resulted in decreased activity of the ipsilateral S1HL^Glu^ neurons (Supplemental Figure 21 and Figure 7, H–J).

Consistent with the effects of mibefradil (Mibe), preoperative microinfusion of the VPL with GABA_A_ receptor agonist muscimol resulted in amelioration of RIH (Supplemental Figure 22, A and B), while the S1HL^Glu^ neuronal firing rate decreased and rheobase increased in brain slices of these mice compared to that of ACSF-treated *CaMKII-Ai14* RIH mice on postoperative day 1 (Supplemental Figure 22, C–F). These results indicate that the enhanced excitability of S1HL^Glu^ neurons in RIH mice was dependent on aberrant hyper-excitatory inputs from VPL^Glu^ neurons. Furthermore, pain sensitization was relieved by the selective preoperative inhibition of S1HL^Glu^ neurons with chemogenetic inhibitory hM4Di virus (AAV-CaMKII-hM4Di-mCherry or AAV-CaMKII-GFP) and intraperitoneal injection of its ligand clozapine-*N*-oxide (CNO) in RIH mice (Supplemental Figure 23, A–D). Electrophysiological recordings showed that preoperative treatment with CNO in the RIH mice led to a significantly decreased neuronal excitability in hM4Di^+^ S1HL^Glu^ neurons compared with those in GFP^+^ S1HL^Glu^ neurons on postoperative day 1 (Supplemental Figure 23, E–I). Cumulatively, these findings demonstrated that the S1HL^Glu^ neurons are functionally responsible for the development of RIH.

To investigate whether the hyperactivity of S1HL^Glu^ neurons is driven by a remifentanil-induced increase in VPL^Glu^ neuron activity in RIH mice, we applied chemogenetics to inhibit VPL^Glu^ terminal activity in the S1HL following ipsilateral VPL injection of chemogenetic inhibitory hM4Di virus and ipsilateral S1HL injection of CNO (Figure 7, K–O). We found that S1HL^Glu^ neuronal activity was reduced and pain sensitization was abolished in the contralateral hindpaws at 30 minutes after CNO injection in *CaMKII-Ai14* RIH mice (Figure 7, P–S), while aversion to the noxious stimuli did not change (Figure 7, T and U). These results suggested that the VPL^Glu^→S1HL^Glu^ circuit is responsible for sensory pain but not the emotional pain component in the current animal model.

In order to explore whether the direct administration of remifentanil to brain slices of RIH mice could recapitulate the same effects on the VPL^Glu^→S1HL^Glu^ pathway as an in vivo exposure, we examined neuronal excitability of VPL^Glu^ and S1HL^Glu^ neurons in thalamocortical brain slices containing the VPL and S1HL using whole-cell recordings with perfusion of remifentanil (8 μM) (Supplemental Figures 24 and 25). We found that the evoked burst firings of ipsilateral VPL^Glu^ neurons significantly diminished after remifentanil perfusion in slices from both naive and incisional mice (Supplemental Figure 24). Interestingly, upon washout of remifentanil, incisional mice showed a marked increase in the number of spikes and a significant decrease in the rheobase of burst firing in ipsilateral VPL^Glu^ neurons compared with the baseline (Supplemental Figure 24, G–L). This phenomenon was not observed in naive mice (Supplemental Figure 24, A–F). In addition, the evoked firing in S1HL^Glu^ neurons was not affected by administration (or washout) of remifentanil in slices from naive or incisional mice (Supplemental Figure 25). It is possible that, although the efferent fibers projecting from the VPL to the S1HL were largely preserved during preparation of the thalamocortical slices, the neural connections may have been destroyed. In addition, as a potent agonist of MORs, differences in the effects of remifentanil between VPL^Glu^ and S1HL^Glu^ neurons may be due to asymmetric distribution of MORs throughout these 2 brain regions; this differential distribution would align well with a previous report that showed *Oprm1* mRNA, which encodes MORs, is widespread in the thalamus, but less so in the cortex (39).

### Functional role of the VPL^Glu^→S1HL^Glu^ pathway in chronic pain.

To investigate the relevance of the VPL^Glu^→S1HL^Glu^ pathway to chronic pain, we employed a persistent spared nerve injury–induced (SNI-induced) neuropathic pain model (Supplemental Figure 26A). At 21 days after induction of the SNI model (SNI 21D), the mice displayed significantly lower mechanical and thermal thresholds compared with those of sham mice (Supplemental Figure 26, B and C). In vivo multi-channel electrode recordings showed that spontaneous spike firing and burst firing rates of contralateral VPL^Glu^ and S1HL^Glu^ neurons were both significantly increased in SNI 21D mice compared with those in sham mice (Supplemental Figure 26, D–I). In addition, chemogenetic inhibition of VPL^Glu^ terminal activity in the S1HL led to a significant increase in the mechanical and thermal pain thresholds of SNI 21D mice (Supplemental Figure 26, J–L). Thus, the activity of the VPL^Glu^→S1HL^Glu^ thalamocortical circuit was enhanced in mice undergoing both acute and chronic pain. This finding was unsurprising,since the VPL has been previously described as a relay station for pain-signal transmission, and the somatosensory thalamocortical structure is indispensable for pain modulation (40–42).

## Discussion

**Figure 8:**
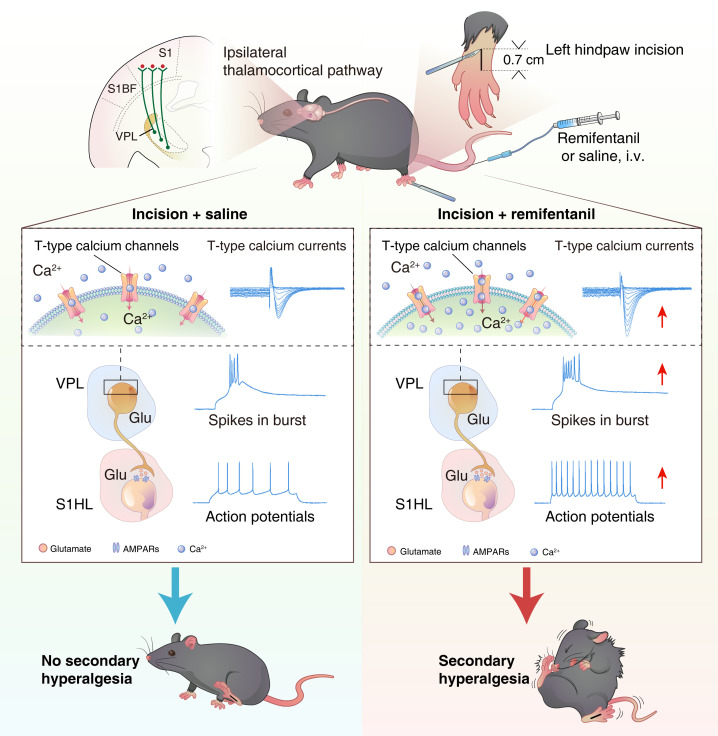
Remifentanil-induced functional upregulation of T-type calcium channels enhances thalamocortical VPL^Glu^→S1HL^Glu^ circuit activity to promote secondary pain in mice. Plantar incision to the left hindpaw with intra-operative remifentanil infusion leads to enhanced burst firing via increased T-type calcium channel activity in ipsilateral VPL^Glu^ neurons. The elevated ipsilateral VPL^Glu^ neuronal burst firing upregulates the sEPSCs in ipsilateral S1HL^Glu^ neurons. The subsequent strong excitation of ipsilateral S1HL^Glu^ neurons was associated with central sensitization and postoperative secondary pain in right hindpaws of mice.

This study identified a mechanism wherein hyperalgesia induced by intraoperative infusion of remifentanil is dependent on upregulation of burst firing of VPL^Glu^ neurons mediated by dysfunction of Ca_v_3.1 channels. This aberrant upregulation of burst firing regulates an excitatory thalamocortical VPL^Glu^→S1HL^Glu^ pathway (Figure 8).

In the current work, we found that remifentanil infusion alone did not affect the pain threshold in naive mice, while incision operation with remifentanil infusion induced postoperative hyperalgesia. Strikingly, remifentanil also produced postoperative secondary mechanical hyperalgesia, but not thermal hyperalgesia, in the contralateral hindpaws of incisional and CFA mice. Previous animal studies have reported that mechanical hyperalgesia is faster and more pronounced than thermal hyperalgesia after opioid exposure (43–46). It is also worth noting that differential pain sensitization between mechanical and thermal stimulation has been reported in healthy human volunteers (47) and minor eye surgery patients (48) after remifentanil administration. It therefore seems plausible that these 2 types of pain processing may have different neural mechanisms in the current RIH mouse model. Based on our findings, we speculated that remifentanil exposure and surgical trauma are necessary factors for triggering the central sensitization of pain in mice. Of note, the apparent lack of hyperalgesia from sufentanil administration is possibly because the dose of sufentanil used in the present study is much lower than that of remifentanil.

Previous mechanistic studies of RIH have mainly focused on the spinal cord (49–52) and dysregulation of the descending pain-modulatory system (16, 53). The functions of supra-spinal structures, especially the thalamocortical circuit, in modulation of RIH are not yet known. Given that nociceptive inputs are transmitted directly to the thalamus through the spinothalamic tract (9), the hyperactive firing response of VPL^Glu^ neurons in RIH mice is plausible. Interestingly, our results in this study indicate that elevated burst firing in ipsilateral VPL^Glu^ neurons likely represent the greatest contributing factor to the observed increase in total firing frequency in RIH mice. These findings are in agreement with previous studies, which reported that changes in burst firing in the thalamus play an essential role in pain signal transmission (13, 21).

As an underlying current of burst firing, both the expression and activity of Ca_v_3.1 channels are dysregulated in pain processing (22, 54). For example, Ca_v_3.1^–/–^ mice display decreased mechanical hypersensitivity and reduced low-frequency rhythms in the primary somatosensory cortex and thalamic ventral posteromedial nucleus in neuropathic pain models (23, 24). These earlier reports are consistent with our findings that show genetic knockdown of Ca_v_3.1 in ipsilateral VPL^Glu^ neurons of RIH mice can reverse the model-induced increase in T-type calcium currents and alleviate hyperalgesia. By contrast, another study showed that the behavioral response to visceral pain is enhanced in Ca_v_3.1^–/–^ mice, which may be caused by loss of burst firing in thalamocortical neurons (55). However, based on findings of impaired opioid-dependent and stress-induced analgesia in mice with periaqueductal gray-specific Ca_v_3.1-knockdown, it is reasonable to conclude that Ca_v_3.1 channels in GABAergic neurons of the periaqueductal gray function in the stress-induced descending analgesia system (56). Thus, the enhanced visceral pain response observed in Ca_v_3.1^–/–^ mice does not, itself, conclusively support a role of Ca_v_3.1 channels in thalamic pain-sensory gating (21). These collective lines of evidence also indicate that Ca_v_3.1 channels may play distinct roles in different types of pain (e.g., visceral pain, neuropathic pain, and incisional pain). In addition, the function of Ca_v_3.1 in pain is also likely related to the distribution of these channels throughout the central nervous system (e.g., somatosensory cortex, thalamus, PAG, and spinal cord) and different types of neurons (e.g., glutamatergic and GABAergic neurons).

Notably, even though remifentanil produced a significant increase in T-type calcium current density in ipsilateral VPL^Glu^ neurons in RIH mice, Ca_v_3.1 expression did not increase in the VPL beyond that produced by the incision. It is therefore reasonable to speculate that surgical trauma upregulates the expression of Ca_v_3.1 protein, and remifentanil exposure further enhances the function of Ca_v_3.1 channels. A number of studies have demonstrated that ion channel activity is subject to multiple layers of posttranscriptional regulation (such as glycosylation, formation of disulphide bonds, phosphorylation, ubiquitination, and enzymatic cleavage (5, 57, 58)). For example, asparagine (Asn, N)-linked glycosylation (N-glycosylation) has been shown to affect the structural folding, membrane targeting, stability, and voltage-dependent properties of many ion channels, including acid-sensing ion channel-1a, 2-pore-domain potassium channels, hyperpolarization-activated cyclic nucleotide gated channels, Piezo1 channels, and T-type calcium channels (59–65). Thus, remifentanil probably enhances Ca_v_3.1 channel activity through posttranslational modifications or other modulatory events. Despite these cumulative findings, the exact mechanisms by which remifentanil exposure and surgical trauma coordinately regulate the expression and trafficking activity of Ca_v_3.1 channels in VPL^Glu^ neurons remains a persistent question for current and ongoing studies.

In the processing of different pain signals, cortices perform essential functions in distinguishing the physical location and type of pain stimulus by acting as a point of convergence and relay in the ascending pain pathway (3, 8, 37). Studies in different animal models have reported that neuronal and synaptic activity of S1HL^Glu^ neurons is enhanced under pain sensing (38, 66), while human fMRI studies have shown that remifentanil administration can activate the primary somatosensory cortex (15, 16). In the current study, we show that the S1HL^Glu^ neurons receive excitatory monosynaptic projections from VPL^Glu^ neurons and that S1HL^Glu^ neuronal excitability is increased in RIH mice. This effect and RIH can be abolished by downregulation of Ca_v_3.1 channels in VPL^Glu^ neurons. Moreover, preoperative chemogenetic inhibition of S1HL^Glu^ neurons can also significantly relieve postoperative pain sensitization. Together, these findings lead us to propose that, although the S1HL is a critical region responsible for RIH, burst firing in VPL^Glu^ neurons plays an essential role in modulating cortical activity.

In summary, our study demonstrates that hyperactive burst firing in VPL^Glu^ neurons caused by dysregulation of Ca_v_3.1 channels underlies the participation of the VPL^Glu^→S1HL^Glu^ pathway in the development of RIH. Our findings provide a novel molecular and circuitry mechanism of RIH, which indicates that T-type channel blockers could potentially serve as an effective approach for prevention of postoperative hyperalgesia.

## Methods

The detailed description of the materials and methods used is provided in Supplemental Methods.

### Study approval.

All experimental procedures on mice were approved by the Animal Care Committee of the University of Science and Technology of China (USTCACUC190101).

## Author contributions

ZZ and WT conceptualized the study. YJ, YM, DC, YT, and RH developed the methodology used and performed experiments. YJ, YM, DC, and YT were responsible for developing the software. CY, JZ, LC, XL, and EG performed experiments. YJ, YM, DC, YT, RH, and CJ analyzed results. YJ, YM, DC, and WT wrote the original draft of the manuscript. YJ, YM, ZZ, and WT edited the manuscript. YJ, YM, ZZ, and WT provided funding for the project. LC and XL provided resources. YJ, ZZ, and WT supervised the project. All authors read and discussed the manuscript. YJ was listed first in the order of co–first authors because YJ was responsible for all experimental designs; performed research; collected, analyzed, and interpreted data; performed statistical analysis; and drafted and revised the manuscript.

## Supplementary Material

Supplemental data

Supplemental video 1

Supplemental video 2

## Version 1 12/15/2022

Electronic publication
