# Supplementary material for: Thalamocortical circuits drive remifentanil-induced postoperative hyperalgesia
Source: J Clin Invest. 2022 Dec 15;132(24):e158742. doi: 10.1172/JCI158742 (PMC9754001; doi:10.1172/JCI158742)
Supplement: Supplemental data [file jci-132-158742-s153.pdf]

Thalamocortical circuits drive remifentanil-induced postoperative hyperalgesia

Authors: Yan Jin[1,2]†, Yu Mao[1,3]†, Danyang Chen[2], Yingju Tai[2], Rui Hu[4], Chen-Ling Yang[5], Jing Zhou[6], Lijian Chen[3], Xuesheng Liu[3], Erwei Gu[3], Chunhui Jia[2], Zhi Zhang[2]*, and Wenjuan Tao[1,5]*

Affiliations:

[1]Stroke Center and Department of Neurology, The First Affiliated Hospital of USTC, Hefei National Laboratory for Physical Sciences at the Microscale, Division of Life Sciences and Medicine, University of Science and Technology of China, Hefei 230036, PR China

[2]Department of Anesthesiology and Pain Medicine, The First Affiliated Hospital of USTC, Division of Life Sciences and Medicine, University of Science and Technology of China, Hefei 230036, China

[3]Department of Anesthesiology, The First Affiliated Hospital of Anhui Medical University, Hefei 230022, PR China

[4]Department of Anesthesiology, The Third Affiliated Hospital of Anhui Medical University, Hefei 230000, PR China

[5]Department of Physiology, School of Basic Medical Sciences, Anhui Medical University, Hefei 230032, PR China

[6]Department of Head-neck and Breast Surgery, Western district of the First Affiliated Hospital of USTC, Division of Life Sciences and Medicine, University of Science and Technology of China, Hefei 233004, PR China

†These authors contributed equally to this work.

***Correspondence to:**

Zhi Zhang, Professor

Division of Life Sciences and Medicine

University of Science and Technology of China

Tel.: (+86) 551-63602715

E-mail: zhizhang@ustc.edu.cn

Wenjuan Tao, Professor
Department of Physiology
School of Basic Medical Sciences
Anhui Medical University
Tel.: (+86) 551-63600153
E-mail: wjtao01@ahmu.edu.cn

Declaration of interests

The authors declare no competing financial interests.

Supplemental Methods

Animals

C57BL/6J mice (Charles River Laboratory, Stock #:000064) were obtained from Charles River; the *ROSA26*^{Ai14} Cre-dependent tdTomato reporter (*Ai14*, B6.Cg-Gt(ROSA)26Sortm14(CAG-tdTamoto)Hze/J; The Jackson Laboratory, Stock #:007914) and *CaMKII-ires-Cre* (67) (B6.Cg-Tg(Camk2a-cre)T29-1Stl/J; The Jackson Laboratory, Stock #:005359) were purchased from the Jackson Laboratory. Mice were housed under a 12-h light/dark cycle with water and food *ad libitum*. The mouse colony was controlled between 23°C and 25°C ambient temperature at 50% humidity. Transgenic mice had a mixed genetic background, and male mice were included in all experiments. The mice were group-housed five per cage unless a tetrode array was implanted. Due to missed targets, including virus injection and the placement of the cannula, optic fiber, or tetrode, behavioral data and in vivo recordings of some mice were excluded from further analyses.

Mouse pain models

Plantar incisional pain model

The plantar incision was adapted from a previous study in mice (68). Mice were anesthetized with 1% to 3% isoflurane delivered via a nose cone in a sterile operating room. A 7-mm longitudinal incision was made with a number 11 blade through the skin and fascia of the left plantar, starting 2 mm from the proximal edge of the heel and extending toward the toes. The underlying muscles were then elevated with curved forceps and incised longitudinally, leaving muscle insertion and origin intact. After gently pressing the wound to stop the bleeding, the skin was closed with a single 6-0 nylon suture (Prolene, Ethicon Inc.), and the wound was covered with erythromycin antibiotic ointment. After the operation, the mice were placed in a recovery cage with a heating plate. Naïve animals (non-operated mice) only received the anesthesia with isoflurane without left plantar incision.

Neuropathic pain model

Mice were given SNI (spared nerve injury) surgeries under 2%–3% isoflurane anesthesia. The skin of the left thigh was sterilized and incised 3–5 mm longitudinally to expose the

subcutaneous muscles. After blunt dissection of muscle with a glass separation needle, the sciatic nerve bundle, which is composed of the sural, tibial and common peroneal nerves, was exposed. The tibial and common peroneal nerves were separated and ligated using nonabsorbent 4-0 chromic gut, then transected distally to preserve the intact sural nerve. The sciatic nerve was returned to its original position, and the skin was sutured and sterilized with iodophor. A similar procedure was performed in sham mice without any nerve damage.

Inflammatory pain model

A volume of 10 μL complete Freund's adjuvant (CFA, catalog number: F5881, Sigma) was intradermally injected into the plantar surface of the left hindpaw of each mouse under brief isoflurane anesthesia to induce inflammatory pain. Control mice received the same quantity of saline (0.9% NaCl).

Drugs

Remifentanil and sufentanil were purchased from Yichang Renfu Pharmaceutical Co., Ltd., and isoflurane was obtained from RWD Life Science Co., Ltd. Remifentanil (40 μg/kg) and sufentanil (0.5 μg/kg) were dissolved in saline (0.9% NaCl) and infused via tail vein at a rate of 0.6 ml/h in 30 mins using a Harvard Apparatus pump (Biosis S.L., Biologic Systems) based on previous studies in rodents (69, 70). Control mice received the same volume of saline under identical conditions. The relevant information of durgs is reported in Supplemental Table 2.

Behavioral tests

Von Frey tests. Mechanical hyperalgesia was quantified by von Frey filament stimuli to the ventral surface of the hind paw. Individual mice were placed in a polymethyl methacrylate box (5 × 5 × 8 cm) on a wire grid floor. Mice were allowed to habituate to the testing environment for 1 h to achieve immobility before testing. A von Frey filament was inserted onto the midplantar hind paw, and the pressure was gradually increased. A nociceptive-like response was considered when paw withdrawal or licking was clearly observed. The mechanical pain threshold was calculated from the average of five trials. Both hind paws were tested.

Hargreaves tests. The Hargreaves test was used to assess the thermal nociceptive threshold. After habituation in clear plastic chambers on a glass floor for at least 30 min, a radiant heat

beam (IITC, CA, USA) was focused on the plantar surface of the hindpaws until the mouse withdrew its paw, the latency of the paw withdrawal was recorded. A cut-off time of 20 s was used to avoid potential tissue damage. The mean hindpaw withdrawal latency was obtained by averaging three separate applications.

Spontaneous pain behavioral tests. Spontaneous pain behavior was assessed 1 day before and 1, 2, 3 and 4 days after incision. Individual mice were placed in a transparent chamber on a wire grid floor and video-recorded for 30 min. The number of lifting/flinching/shaking events and bouts of licking were manually counted in each recording. Movements associated with grooming, locomotion, exploratory behavior and body repositioning were excluded. The bouts of spontaneous licking and flinching/shaking/lifting of the hindpaw were recorded by a scorer blinded to the cluster firing phenotype. One bout of lifting/flinching/shaking was counted as 1 point, one bout of licking was counted as 2 points, and the total points for the 30 min recording was considered the spontaneous pain score (71).

Real-time place escape avoidance tests (RT-PEAP). Real-time place escape avoidance tests (RT-PEAP) were conducted on the first day post surgery in light-dark boxes placed on a wire mesh grid without a bottom floor (72). The light-dark boxes consisted of a light chamber and a dark chamber of the same size (20 cm × 15 cm × 30 cm); the two chambers were separated by a wall with an open door (5 cm × 5 cm) to allow mice to freely explore the entire apparatus. The mice were allowed to freely explore the light and dark chambers for 15 min (Pre). Subthreshold von Frey stimuli (0.07 g) were applied to the contralateral hindpaws once every 2 s for 15 min once the mice entered the dark chamber (During). The mice were then allowed to freely explore the entire apparatus for 15 min (Post). The travel trajectories were video-recorded and the time spent in each chamber was analyzed using Etho Vision XT software. The aversion ratio for the dark chamber was calculated by dividing the time spent in the Post period by that in the Pre period.

Stereotaxic viral injections

All viral procedures followed the Laboratory Biosafety Guidelines approved by the University

of Science and Technology of China. Mice were anesthetized with pentobarbital (20 mg per kg, i.p.) and stabilized in a stereotaxic apparatus (RWD Life Science Co., Ltd.). After adjusting the level of the skull surface, the holes were drilled through the skull by a dental drill. A pulled glass microelectrode was backfilled with virus and connected to a 10-microliter syringe. The injection volume of different viruses varied from 100 to 300 nl depending on the viral titer and expression potential, and the infusion rate was 30 nl/min. After injection, the microelectrode remained at the injection site for 5 min to avoid the leakage of the virus. The coordinates of an injection site included three dimensions: anterior/posterior (AP) from the bregma, medial/lateral (ML) from the midline, and dorsal/ventral (DV) from the pial surface of the brain.

For optogenetic manipulation of glutamatergic neurons in the VPL, a recombinant adeno-associated virus (AAV) of Ef1α-DIO-ChR2-mCherry-WPRE-pA (AAV-DIO-ChR2-mCherry, AAV2/9, 3.63×10^{12} vg/ml) was ipsilaterally injected into the VPL (A/P, −1.9 mm; M/L, −1.7 mm; D/V, −3.5 mm) or S1HL (A/P, −1.7 mm; M/L, −1.0 mm; D/V, −0.5 mm) of *CaMKII-Cre* mice. Three weeks later, optogenetic stimulation was performed. For eNpHR3.0-induced bursting, AAV-Ef1α-DIO-eNpHR3.0-EYFP-WPRE-pA (AAV-DIO-eNpHR3.0-EYFP, AAV2/9, 5.63×10^{12} vg/ml) was unilaterally injected into the VPL of *CaMKII-Cre* mice. Three weeks later, optogenetic stimulation was performed. For chemogenetic inhibition of $S1HL^{Glu}$ neurons, AAV-CaMKII-hM4D(Gi)-EGFP-WPRE-pA (AAV-CaMKII-hM4Di-EGFP, AAV2/9, 5.63×10^{12} vg/ml) was ipsilaterally injected into the S1HL of C57 mice. Three weeks later, CNO (5 mg/kg, catalog number: D9542, Sigma) was intraperitoneally injected 30 min prior to the operation. For chemogenetic inhibition of VPL^{Glu} neurons projecting into the S1HL, AAV-CaMKII-hM4Di-EGFP was ipsilaterally injected into the VPL of C57 mice, and the cannula was implanted into the ipsilateral S1HL. Three weeks later, a volume of 300 nl CNO (1 nM) was intracranially injected 30 min before behavioral tests. AAV-Ef1α-DIO-mCherry-WPRE-pA (AAV-DIO-mCherry, AAV2/9, 5.14×10^{12} vg/ml), AAV-Ef1α-DIO-EYFP-WPRE-pA (AAV-DIO-EYFP, AAV2/9, 3.42×10^{12} vg/ml) and AAV-CaMKII-EGFP-WPRE-pA (AAV-CaMKII-GFP, AAV2/9, 5.14×10^{12} vg/ml) were used as control viruses.

For anterograde monosynaptic tracing, AAV-*hSyn*-EGFP-P2A-Cre-WPRE-pA (AAV-Cre-GFP, AAV2/1, 1×10^{13} vg/ml) was injected into the VPL of C57 to drive Cre-dependent transgene expression in the postsynaptic neurons. Simultaneously, AAV-Ef1α-DIO-EGFP-WPRE-pA (AAV-DIO-GFP, AAV2/9, 5.08×10^{12} vg/ml) was injected into the ipsilateral S1HL. After injection, mice were housed for 3 weeks before euthanasia.

For retrograde monosynaptic tracing, a 200 nl volume of helper viruses containing AAV-Ef1α-DIO-ΔRVG-WPRE-pA (AAV-DIO-RVG, AAV2/9, 4.59×10^{12} vg/ml) and AAV-Ef1α-DIO-H2B-EGFP-T2A-TVA-WPRE-pA (AAV-DIO-TVA-GFP, AAV2/9, 5.56×10^{12} vg/ml; 1:2) was injected into the S1HL of *CaMKII-Cre* mice. Three weeks later, 300 nl RV-EnvA-ΔG–dsRed (2×10^{8} IFU/ml) was injected into the same site of the S1HL. The helper viruses facilitated the spread of monosynaptic retrograde RV. Starter cells (yellow) co-expressing AAV-DIO-TVA-GFP, AAV-DIO-RVG (green), and rabies RV-EnvA-ΔG-DsRed (red). After injection, mice were housed in a biosafety level 2 facility for 7 days before euthanasia.

For the specific genetic knockdown experiments, AAV-CaMKII-mCherry-mir30-$Ca_v3.1$-shRNA (AAV-RNAi, AAV2/9, 6.28×10^{12} vg/ml) was used to knock down the expression of $Ca_v3.1$ in the VPL, and AAV-CaMKII-mCherry-mir30-scramble-shRNA (AAV-control, AAV2/9, 5.22×10^{12} vg/ml) was used as the control. Three weeks later, behavioural experiments were performed. For optogenetic manipulation of glutamatergic neurons with genetic knockdown of $Ca_v3.1$ in the VPL, 300 nl of mixed virus solution (1:1) AAV-CaMKIIa-eNpHR3.0-EYFP-WPRE-hGH-pA (AAV-CaMKIIa-eNpHR3.0-EYFP, AAV2/9, 5.13×10^{12} vg/ml) and AAV-RNAi were unilaterally delivered into the VPL. The same dose of mixed virus solution AAV-CaMKIIa-eNpHR3.0-EYFP-WPRE-hGH-pA (AAV-CaMKIIa-eNpHR3.0-EYFP, AAV2/9, 5.13×10^{12} vg/ml) and AAV-control were used as the control. Three weeks later, optogenetic stimulation was performed.

All viruses mentioned above were purchased from BrainVTA. The relevant information of viruses is reported in Supplemental Table 2. Mice were anesthetized with intraperitoneal injection of pentobarbital (20 mg/kg) and transcranially perfused with ice-cold saline followed by 4% paraformaldehyde (PFA). Images of virus expression were acquired using a Zeiss LSM

880 confocal microscope. Mice with missed targets were excluded from data analysis.

Cannula infusion experiment

After the surface of the skull was leveled in the stereotaxic apparatus, a cannula (diameter of 0.25 mm, length of 4.5 mm, RWD) was implanted into the VPL and secured to the surface of the skull with dental cement. Seven days after implantation, a volume of 300 nl mibefradil (catalog number: M5441, Sigma) dissolved in artificial cerebrospinal fluid (ACSF) was injected into the VPL at a rate of 100 nl/min through the cannula 30 min before performing plantar incision. To explore the effects of postoperative mibefradil administration on pain sensitization, mibefradil or ACSF were injected into the ipsilateral VPL at 30 min prior to behavioral tests every day after incision. The mice in the control group were intracranially injected with the same volume of ACSF. Mice were euthanized after all behavioral tests, and fluorescent image acquisition with DAPI (4′,6-diamidino-2-phenylindole) staining was performed with the Zeiss LSM 880 confocal microscope. Data from mice with incorrect injection sites were excluded from analysis.

Immunohistochemistry and imaging

Mice were deeply anesthetized and perfused with ice-cold saline followed by 4% PFA through the left ventricle. The brains were extracted and soaked in the 4% PFA at 4°C overnight and then immersed in 20% and 30% sucrose solution for dehydration until they sank. Brains were cut into coronal slices with thickness of 40 μm using a cryostat microtome system (Leica CM1860) at −20°C. Brain slices were soaked in antifreeze and stored at −20°C. For staining, brain slices were first washed 3 times with phosphate-buffered saline (PBS) for 10 min and then blocked with 10% donkey serum in PBS with 5% Triton X-100 at room temperature for 1 hr. Brain slices were next incubated with primary antibodies in PBS with donkey serum and 3% Triton X-100 at 4°C overnight. The primary antibodies included rabbit anti-glutamate (1:500, catalog number: G6642, Sigma), rabbit anti-c-Fos (1:500, catalog number: 226003, SYSY), rabbit anti-GABA (1:500, catalog number: A2052, Sigma), mouse anti-glutamate (1:100, catalog number: G9282, Sigma), rabbit anti-Ca_v 3.1 (1:100, catalog number: PA577311, Thermo). Finally, slices were washed 3 times with PBS for 10 min and incubated with

secondary antibodies in PBS with 3% Triton X-100 for 1.5 hr at room temperature. The secondary antibodies included donkey anti-rabbit IgG Alexa 488 (1:500, catalog number: A21206, Invitrogen), donkey anti-mouse IgG Alexa 594 (1:500, catalog number: A21203, Invitrogen), donkey anti-rabbit IgG Alexa 594 (1:500, catalog number: A21207, Invitrogen) donkey anti-rabbit IgG Alexa 647(1:500, catalog number: A31573, Invitrogen). Slice images were visualized and acquired with the Zeiss LSM 880, and further analyses, such as cell counts and colocalization, were performed using ImageJ software (Fiji edition, National Institutes of Health) by an assistant blinded to the condition. The relevant information of antibodies is reported in Supplemental Table 2.

In vitro electrophysiological recordings of brain slice

Brain slice preparation. Mice were deeply anesthetized and then perfused through the left ventricle with ice-cold oxygenated N-methyl-D-glucamine (NMDG)-based artificial cerebrospinal fluid (NMDG ACSF), which contained (in mM) 20 HEPES buffer, 93 NMDG, 2.5 KCl, 25 glucose, 1.2 NaH_2PO_4, 0.5 $CaCl_2$, 30 $NaHCO_3$, 5 Na-ascorbate, 10 $MgSO_4$, 3 Na-pyruvate, 3 glutathione, and 2 thiourea (pH 7.3–7.4, osmolarity of 300–305 mOsm). The brain was then extracted and sectioned into coronal slices (300 μm) or thalamocortical slices (400 μm) using a vibrating microtome system (VT1200s, Leica). For mice in which eNpHR3.0 or ChR2 virus was injected into the VPL to verify the function of the thalamocortical connection through optogenetic regulation, the ventral side of the brain with the rostral part pointing down the slope was glued on an agar with a horizontal angle of 50° to preserve the maximum integrity of projection fibers from VPL^{Glu} to the S1HL (73). All brain slices were initially incubated in oxygenated NMDG ACSF at 33°C for 10 min and then recovered in oxygenated N-2-hydroxyethylpiperazine-N'-2-ethanesulfonic acid (HEPES)-buffered ACSF at 28°C for at least 1 hr, which contained (in mM) 2.5 KCl, 92 NaCl, 1.2 NaH_2PO_4, 2 $CaCl_2$, 30 $NaHCO_3$, 3 Na-pyruvate, 5 Na-ascorbate, 20 HEPES, 2 $MgSO_4$, 25 glucose, 2 thiourea, and 3 GSH (pH: 7.3–7.4, osmolarity: 300–310 mOsm). Brain slices were subsequently transferred into a slice chamber (Warner Instruments) for electrophysiological recording and continuously perfused with oxygenated standard ACSF at 32°C, which contained (in mM) 3 KCl, 129 NaCl, 20

$NaHCO_3$, 2.4 $CaCl_2$, 1.3 $MgSO_4$, 1.2 KH_2PO_4, 3 HEPES, and 10 glucose (pH: 7.3–7.4, osmolarity: 300–310 mOsm).

Whole-cell patch-clamp recording. Cells in the VPL or S1HL were visualized with a water immersion objective (×40) in an infrared-differential interference contrast microscope (BX51Wl, Olympus). A MultiClamp 700B amplifier and pCLAMP10.7 software were applied to collect electrophysiological signals. After a stable Gigaseal was formed, the capacitance and series resistance were automatically compensated. Whole-cell patch-clamp recordings were performed between 2 and 5 min after break-in. The current-evoked firing and burst firing were recorded under current clamp (I = 0 pA) using pipettes (5-8 MΩ) filling with potassium-gluconate-based internal solution, containing (in mM) 130 K-gluconate, 5 KCl, 2 $MgCl_2$, 10 HEPES, 0.6 EGTA, 0.3 Na-GTP and 2 Mg-ATP (pH 7.2, osmolality of 285–290 mOsm). For recording T-type calcium currents of VPL^{Glu} neurons, the patching pipettes were filled with Cs-methanesulfonate-based internal solution containing (in mM) 130 Cs-methanesulfonate, 0.15 $CaCl_2$, 2 $MgCl_2$, 2 EGTA, 10 HEPES, 2 Na_2-ATP, 0.25 Na_3-GTP and 10 QX-314 (pH: 7.2, osmolarity: 282 mOsm). The recordings were made at least 5 min after establishing a whole cell configuration with a stable resting membrane potential. Unless stated otherwise, the drug was applied with perfused ACSF.

Burst and T-type calcium current recording. VPL^{Glu} neurons have the property of spontaneous burst activity represented by generating clusters of spikes. Spontaneous and current-evoked burst firing were recorded under current-clamp mode (I_{hold} = 0 pA), and the current-evoked burst firing was obtained with a series hyperpolarized currents (from −10 pA to −300 pA, −10 pA/step, 500 ms) delivered to neurons. To directly separate T-type calcium channel-mediated currents, the membrane voltage of VPL^{Glu} neurons was held at −60 mV with 500 ms-long voltage steps of −115 mV through −50 mV (−5 mV/step) and the tetrodotoxin (TTX, 1 μM), 4-aminopyridine (4-AP, 4 mM), CsCl (2 mM) and Tetraethylammonium Chloride (TEACl, 10 mM) were added into the ACSF. Data of current-evoked firing was only collected from neurons with a resting membrane potential lower than −50 mV. The current–voltage (I-V) curve, which changed in the membrane potential as a function of intracellular injected currents (−10 to −60

pA, −10 pA/step) was plotted, and its slope was derived from the linear range of the curve. The slope of the *I-V* curve was defined as the input resistance of the cell membrane. The rheobase for current-evoked firing (tonic or burst) was defined as the minimum strength of current injection required to elicit at least one or two spikes.

Light-evoked response. Light-evoked burst firings were recorded in eNpHR3.0^{+} VPL^{Glu} neurons while pulsed yellow light (589 nm, 1 Hz, 100 ms) was delivered through an optical fiber positioned 0.2 mm above the VPL brain slices. Spontaneous excitatory postsynaptic currents (sEPSCs) were recorded in S1HL^{Glu} neurons in the presence of picrotoxin (PTX, 50 μM) with a holding potential of −70 mV while photostimulating eNpHR3.0^{+} VPL^{Glu} soma in thalamocortical somatosensory slices. To verify the synaptic functionality of VPL^{Glu}→S1HL^{Glu} neurons, light-evoked EPSCs were recorded in S1HL^{Glu} neurons with a holding potential of −70 mV while photostimulating (473 nm, 20 ms, 2 Hz) ChR2^{+} VPL^{Glu} soma in thalamocortical somatosensory slices. The TTX (1 μM), 4-AP (4 mM), and AMPA receptor antagonist receptor antagonist 6,7-dinitroquinoxaline-2,3(1H,4H)-dione (DNQX, 20 μM) were used to verify monosynaptic excitatory glutamatergic projections. Drugs used for electrophysiology were dissolved in ACSF to aliquot at 1000 × final concentration and stored at −20°C before use.

Western blot

Ipsilateral VPL tissues were quickly obtained from 300 μm-thick slices cutting on the vibratome. Membrane protein was extracted using a membrane and cytoplasmic extraction kit (Sangon Biotech, Shanghai, China, catalog number: C510005) following manufacturer's instructions. To extract total protein, the tissues were homogenized in ice-cold RIPA buffer, which contained 50 mM Tris-HCl (pH 7.6), 1% Triton X-100, 150 mM NaCl, 0.1% SDS, a protease inhibitor cocktail, and 0.5% sodium deoxycholate. The protein concentration was determined using a bicinchoninic acid (BCA) kit (Thermo, catalog number:23225). The lysates were separated by sodium dodecyl sulfate polyacrylamide gel electrophoresis (SDS-PAGE), with the isolated protein transferred onto a polyvinylidene fluoride (PVDF) membrane set to a constant voltage of 80 V. Following 1h of blocking after electrophoresis, the membrane was incubated with

diluted primary antibodies overnight at 4°C. The primary antibodies included antibodies for $Ca_v3.1$ (1:700, catalog number: PA577311, Thermo), β-actin (1:1000, catalog number: abs137975, Absin), Na, K-ATPase (1:700, catalog number: 3010s, CST). Subsequently, either goat anti-rabbit IgG (1:50,000, catalog number: 31466, Invitrogen) or goat anti-mouse IgG (1:5000, catalog number: 115-035-003, Jackson) were used as secondary antibodies for 1.5 h incubation at room temperature. The relevant information of antibodies is reported in Supplemental Table 2. Protein bands were visualized by chemiluminescence and quantified using ImageJ software.

In vivo calcium signal recording

After adjusting the level of skull surface in the stereotaxic apparatus, 200 nl AAV-CaMKII-GCaMP6m-WPRE-pA (AAV-CaMKII-GCaMP6m, AAV2/9, 5.46×10^{12} vg/ml) was delivered into the ipsilateral VPL or S1HL of C57 mice at a rate of 50 nl/min. The optical fiber (the core of 200 μm, Newdoon) was implanted in the site of the viral injection, and cemented on the skull with screws and dental cement. After surgery, mice recovered on a heating pad and then were housed in a stable environment for 2 weeks before the experiment. A multi-channel fiber photometry device (Inper-C1-3C, Inper) delivered exciting LEDs (410 nm and 470 nm) to excite GCaMP6m fluorophore and collect the emission through the patch cable (0.37 NA, 200 μm, Inper) and the implanted optical fiber. The region of interest around the fiber was drawn out to maintain the average intensity, and the behavioral videos were synchronized with the neuronal calcium signals using TTL pulses during recording. A time stamp was assigned to a single recorded time point in order to be consistent with a specific time and event during recording. Calcium signals were digitized using a digital signal acquisition board and demultiplexed using a software lock-in amplifier; then, signals were low-pass filtered to 30 Hz and saved to a disk at a rate of 381 samples/sec. Photometry data were next analyzed with the Inper Data Process. The values of fluorescence change (ΔF/F), calculated as $(F_{signal} - F_{baseline})/F_{baseline} \times 100$, are presented as heatmaps or average plots with the SEM. $F_{baseline}$ is the mean of fluorescence signal for 5 seconds prior to the von Frey stimulus, and F_{signal} is the

fluorescence signal for the entire session (74).

In vivo optogenetic stimulation

For mice expressing eNpHR-EYFP or EYFP in the VPL, optical fiber cannulae (the core of 200 μm, Newdoon) were implanted 0.2 mm above the targeting site. Optical fiber cannulae were secured to the skulls of mice with screws and dental cement. Implanted fibers were connected to the laser generator with optical fiber patch cords. The delivery of yellow light (589 nm, 10 mW, 100 ms, 1 Hz) was controlled with a Master-8 pulse stimulator (A.M.P.I.) during each testing session.

In vivo pharmacological approach

A catheter with a diameter of 250 μm (RWD) was implanted into the VPL or S1HL and secured to the skull of the mouse with screws and dental cement. Three hundred nanoliters of Mibefradil (15 nM, catalog number: M5441, Sigma) or MUS (0.3 nM, catalog number: 2763-96-4, Sigma) were administrated at a rate of 100 nl/min into the VPL 30 min before operation. The mice in the control group were intracranially injected with the same volume of ACSF.

In vivo two-photon calcium imaging

Cranial window surgery Mice were deeply anesthetized and fixed on the stereotaxic apparatus. The antibiotic enrofloxacin (125 mg/kg, i.p., MedChemExpress), the antiphlogistic dexamethasone (25 mg/kg, i.p., MedChemExpress), and carprofen (6 mg/kg, s.c., Sigma-Aldrich) were injected before surgery. After adjusting the level of the skull surface, the skull of the S1HL brain area was grinded with a dental drill to make a 3 mm-diameter round shape and gently lifted with tweezers to avoid blood vessels. Meroceles (Fukangsen) were used to stop bleeding, and the dura was kept intact and moisturized with saline. Three hundred nanoliters AAV-CaMKIIα-GCaMP6f-WPRE-pA (AAV-CaMKIIα-GCaMP6f, AAV2/9, 5.45×10^{12} vg/ml) was injected in to the S1HL at a rate of 30 nl/min; 1.2% agarose was dropped to cover the dura surface, and a round coverslip (3 mm, Bellco Glass Inc.) was then inserted to fit into the craniotomy and cemented to the skull using glue (Vetbond tissue adhesive, 3M). A custom-made stainless steel headplate was cemented well around the craniotomy area with glue and dental cement. Mice recovered from anesthesia on the heating plate and were housed 3 per cage

in a stable environment. Mice were injected with carprofen (6 mg/kg, s.c.) 5 days post operation (75, 76).

Two-photon imaging Three weeks after cranial window surgery, mice were adapted to the headplate holder and the imaging environment for 15 mins each day 3 days before imaging. An upright two-photon microscope (FVMPE-RS, Olympus) in frame-scan mode was applied for awake calcium imaging. Real-time images were acquired using FV30S-SW (Olympus) and a macro water objective lens (×25/0.8 NA) equipped with an infrared laser with excitation wavelength of 920 nm. For each slice, 300 frames were obtained at a frequency of 1.5 Hz for 256×256 pixels in the x–y plane. The typical average power (20–30 mW) was applied for image GCaMP6m expressing neurons in S1HL.

Data processing and analysis Time series were imported into ImageJ to correct movement artifacts using TurboReg (77), and sequential images were exported as time-lapse videos. Individual neurons were distinguished from the calcium images using custom MATLAB scripts that implemented component extraction in terms of describing the spatial footprint (shape and location) and the activity trace of the objective. Manual inspection of individual neurons provided quality control. Fluorescence signal time series with ΔF/F trace were automatically analyzed as the fluorescence change based on baseline fluorescence of components. Calcium signal traces (ΔF/F > 0.5) in the soma were identified as significant calcium events using the MATLAB-based open-source tool CaImAn (https://github.com/flatironinstitute/CaImAn-MATLAB), which has previously been used for similar analyses (78). The frequency of GCaMP6f signals in neuronal soma was calculated using the CaImAn "findpeaks" function.

In vivo multi-channel electrode recording

Implantation of tetrodes/optrodes Mice were anesthetized and secured on the stereotaxic apparatus. A custom-made microdrive array attaching 4-8 tetrodes was implanted into the VPL or S1HL. A tetrode was composed of four twisted fine nichrome wires (13 μm, California Fine Wire). In order to perform optogenetic tagging of VPL^{Glu} or $S1HL^{Glu}$ neurons in *CaMKII-Cre* mice, the tetrodes were replaced with optrodes consisting of one optic fiber (the core of 200 μm, Newdoon) surrounded by several tetrodes, with the tip protruding 200 ~ 300 μm beyond

the fiber. The tetrode/optrode was fixed on the brain skull with four screws and dental cement. Mice recovered from anesthesia on a warming plate and thereafter were single-housed.

Electrophysiological recordings Mice were allowed to recover for at least 3 days after surgery and then adapted to having the cables and headstages plugged into the 32-channel connector (Omnetics Connector) for several days prior to recordings. To explore the VPL or S1HL activity in mice, recording was performed on freely moving mice in a chamber. Spikes were digitized at 40 kHz, bandpass filtered at 300 to 5,000 Hz, and stored in a computer with NeuroStudio software for further analysis. Data were analyzed using NeuroExplorer 4 (Plexon). Clusters of spikes in the VPL beginning with a maximal inter-spike interval of 20 ms and ending with a maximal inter-spike interval of 100 ms were identified to be bursts. The minimum number of spikes in a burst was set at 2, and the minimum intra-burst interval was set at 100 ms. Spike firing rate, bursts events per min, and the percentage of spike firing within bursts were analyzed.

Spike sorting Data were exported to Offline Sorter 4 (Plexon) for spike sorting. Units with a signal-to-noise ratio smaller than 2 were excluded from analysis. Principal component analysis and threshold crossing were applied to automatically identify waveforms into individual units. Units with inter-spike intervals longer than the refractory periods (1 ms) were determined to be isolated and included in further analysis. Well-isolated units (L ratio < 0.2, isolation distance > 15) were classified into narrow-spiking interneurons or wide-spiking putative pyramidal neurons using an unsupervised clustering algorithm in terms of a κ-means method (79). The algorithm separated each neuron in terms of three-dimensional parameters, including the mean firing rate, the half-valley width, and half-spike width (trough to peak duration) at baseline. Spikes with a slower firing rate, longer half-valley width and longer half-spike width were distinguished to be putative pyramidal neurons. Most of the pyramidal neurons in the somatosensory cortex are known to be glutamatergic neurons (80).

Optogenetic identification of $S1HL^{Glu}$ or VPL^{Glu} neurons For in vivo optogenetic tagging of $S1HL^{Glu}$ or VPL^{Glu} neurons, *CaMKII-Cre* mice were unilaterally injected with AAV-DIO-ChR2-mCherry aimed at S1HL or VPL (for details, see *Stereotaxic viral injections*). Three weeks later, optrodes were implanted at the same sites at which virus was injected (for details

see *Implantation of tetrodes/optrodes*). Blue-light pulses (470 nm, 2 ms, 20 Hz) were delivered following the end of each recording session. Single units exhibiting time-locked spikes with high reliability (> 90%), low jitter (< 2 ms) and short first-spike latency (< 3 ms) upon light stimulation were considered light responsive. Only when the similarity of waveforms from spontaneous and laser-evoked spikes were very high (correlation coefficient > 0.9), they were considered to be the same neurons.

Optogenetic stimulation on neuron firing. Optrodes were implanted into the VPL or S1HL in *CaMKII-Cre* mice in which AAV-DIO-eNpHR3.0-EYFP was unilaterally injected into the VPL. Neuronal firings were recorded in the presence of yellow-light stimulation (589 nm, 100 ms, 1 Hz) on the somas of VPL^{Glu} neurons in the VPL and on the fibers of VPL^{Glu} neurons in the S1HL.

Randomization and blinding

Mice were randomly assigned to experimental groups and subjected to in vivo and in vitro electrophysiological recordings, two-photon calcium imaging, photometry recordings, and behavioral tests. In each specific experiment, the testers were not blind to the group assignment of the sample because they needed to record the earmarks of the mice. However, the statistical analyst was blinded to the experimental groups.

Statistical analysis

Required sample sizes were calculated based on the results of our pre-experiments. Mice were randomly assigned to each treatment, and the analyses were performed by an assistant blinded to the treatment assignment. GraphPad Prism 8 (GraphPad Software, Inc., USA) and SPSS Statistics V26 software (IBM, NY) were used for statistical analysis and graphing. The relevant information of softwares used is reported in Supplemental Table 2. The D'Agostino & Pearson omnibus normality test and the Brown–Forsythe tests were respectively applied to assess normality and equal variances between groups. Paired or unpaired two-tailed Student's *t*-tests were applied for statistical comparisons between two groups. One-way or two-way analysis of variance (ANOVA) followed by post hoc Bonferroni's test were used for analysis with multiple groups. Repeated measures (RM) were incorporated when appropriate. To exclude the random

effect of the mouse in the electrophysiological experiments, linear mixed models with post hoc Bonferroni's test was used to fit the data. The residual maximum likelihood (REML) method was used to fit the models. Nested *t*-test or nested one-way ANOVA with post hoc Bonferroni's test were used for electropysiological and calcium imaging data. One-sample *t*-test and Chi-square test were used when appropriate. Data are presented as the mean ± SEM otherwise indicated in the figure legends, and $P < 0.05$ was considered to be significant. The sample sizes, specific statistical tests used, and other relevant information of statistical analysis are reported in Supplemental Table 1.

References

67. Dragatsis I, and Zeitlin S. CaMKIIalpha-Cre transgene expression and recombination patterns in the mouse brain. *Genesis.* 2000;26(2):133-5.
68. Pogatzki EM, and Raja SN. A mouse model of incisional pain. *Anesthesiology.* 2003;99(4):1023-7.
69. El Mouedden M, and Meert TF. Evaluation of pain-related behavior, bone destruction and effectiveness of fentanyl, sufentanil, and morphine in a murine model of cancer pain. *Pharmacol Biochem Behav.* 2005;82(1):109-19.
70. Celerier E, Laulin JP, Corcuff JB, Le Moal M, and Simonnet G. Progressive enhancement of delayed hyperalgesia induced by repeated heroin administration: a sensitization process. *J Neurosci.* 2001;21(11):4074-80.
71. Jourdan D, Ardid D, Bardin L, Bardin M, Neuzeret D, Lanphouthacoul L, et al. A new automated method of pain scoring in the formalin test in rats. *Pain.* 1997;71(3):265-70.
72. Fuchs PN, and McNabb CT. The place escape/avoidance paradigm: a novel method to assess nociceptive processing. *J Integr Neurosci.* 2012;11(1):61-72.
73. Varela C, Llano DA, and Theyel BB. In: Fellin T, and Halassa M eds. *Neuronal Network Analysis: Concepts and Experimental Approaches*. Totowa, NJ: Humana Press; 2012:103-25.
74. Jia H, Rochefort NL, Chen X, and Konnerth A. In vivo two-photon imaging of sensory-evoked dendritic calcium signals in cortical neurons. *Nat Protoc.* 2011;6(1):28-35.
75. Garaschuk. O, and Konnerth. A. In vivo two-Photon calcium imaging using multicell bolus loading. *Cold Spring Harb Protoc.* 2010;doi:10.1101/pdb.prot5482.
76. Ohki. K, and Reid. RC. In vivo two-photon calcium imaging in the visual system. *Cold Spring Harb Protoc.* 2014;doi:10.1101/pdb.prot081455.
77. Schindelin J, Arganda-Carreras I, Frise E, Kaynig V, Longair M, Pietzsch T, et al. Fiji: an open-source platform for biological-image analysis. *Nat Methods.* 2012;9(7):676-82.
78. Giovannucci A, Friedrich J, Gunn P, Kalfon J, Brown BL, Koay SA, et al. CaImAn an open source tool for scalable calcium imaging data analysis. *Elife.* 2019;8.
79. Xu H, Liu L, Tian Y, Wang J, Li J, Zheng J, et al. A Disinhibitory Microcircuit Mediates Conditioned Social Fear in the Prefrontal Cortex. *Neuron.* 2019;102(3):668-82.e5.
80. Lodato S, and Arlotta P. Generating neuronal diversity in the mammalian cerebral cortex. *Annu*

Rev Cell Dev Biol. 2015;31:699-720.

Supplemental Figure titles and legends

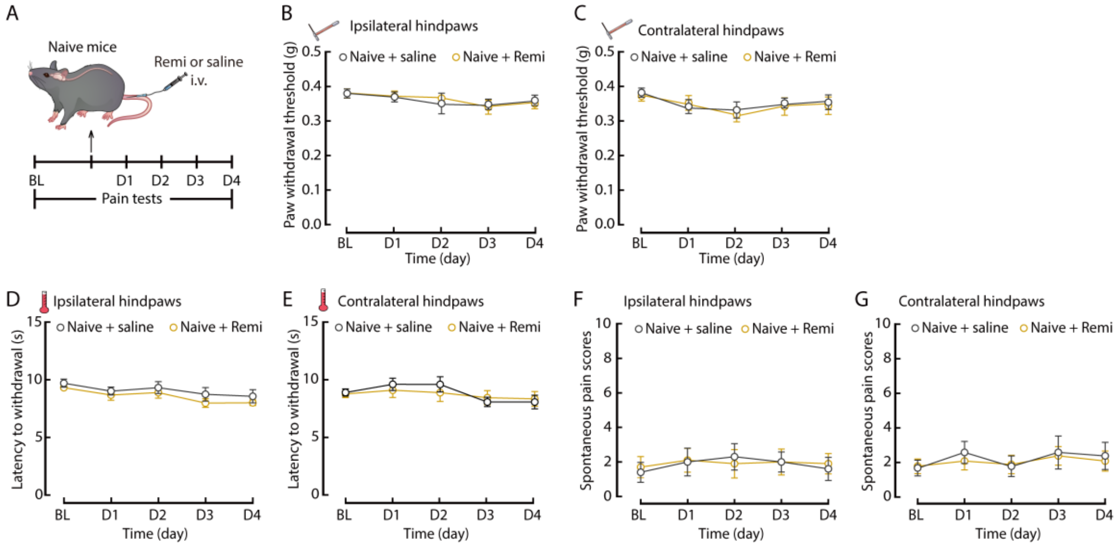

Supplemental Figure 1 | Sensory pain tests in naïve mice.

(A) Schematic of the experimental procedure for behavioral tests.

(B and C) Time course of pain threshold by von Frey tests in ipsilateral (**B**, $F_{(1,14)} = 0.2597$, $P = 0.6182$) and contralateral hindpaws (**C**, $F_{(1,14)} = 0.0017$, $P = 0.9676$) of naïve mice treated with Remi or saline ($n = 8$ mice per group).

(D and E) Time course for thermal nociceptive thresholds of the ipsilateral (**D**, $F_{(1,18)} = 1.793$, $P = 0.1972$) and contralateral hindpaws (**E**, $F_{(1,18)} = 0.0673$, $P = 0.7982$) in naïve mice treated with saline or Remi ($n = 10$ mice per group).

(F and G) Time course for spontaneous pain scores of the ipsilateral (**F**, $F_{(1,18)} = 0.0165$, $P = 0.899$) and contralateral hindpaws (**G**, $F_{(1,18)} = 0.1455$, $P = 0.7074$) in naïve mice treated with saline or Remi ($n = 10$ mice per group).

Data: mean ± SEM. Two-way RM ANOVA with post hoc Bonferroni's test in (**B-G**).

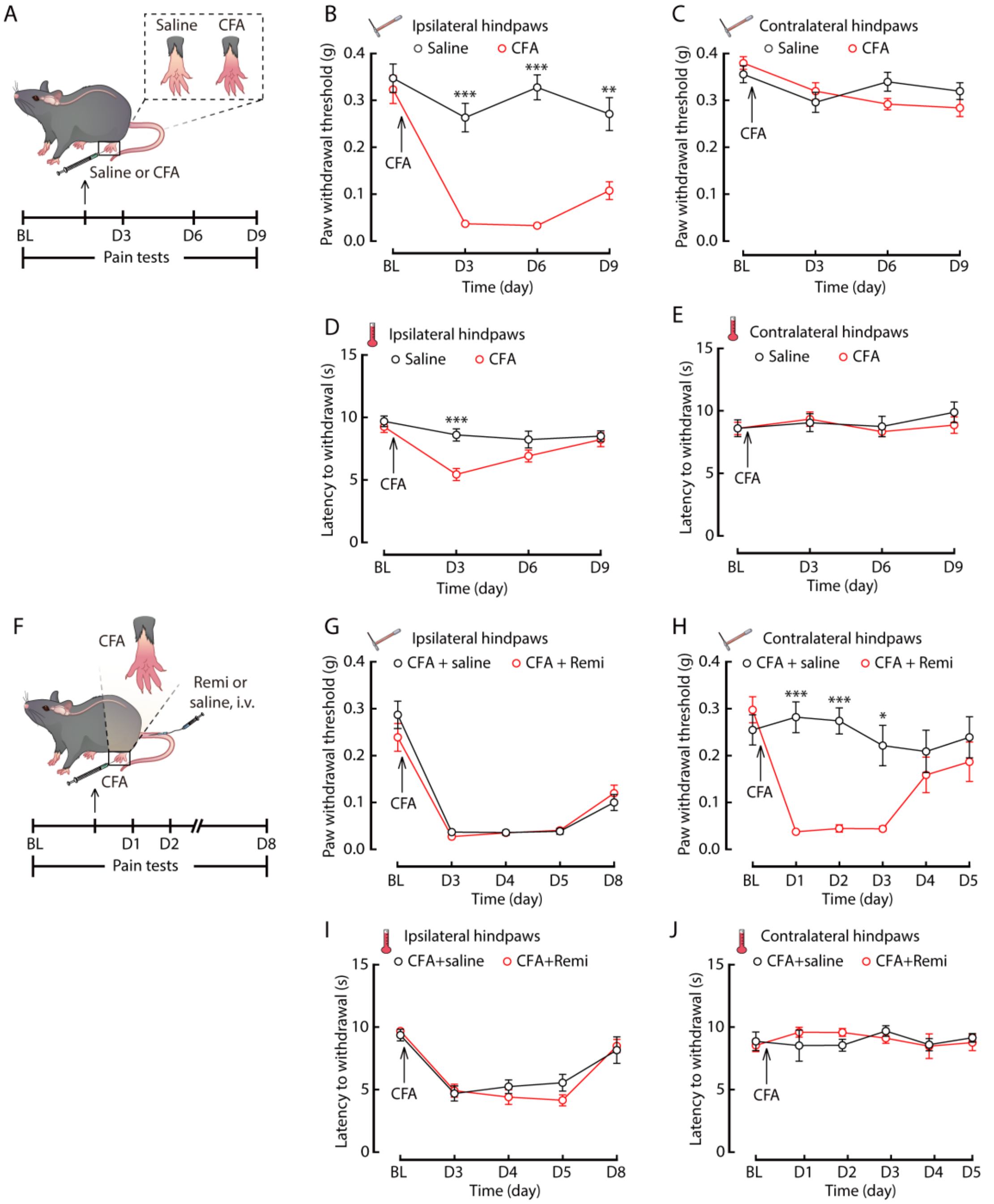

Supplemental Figure 2 | Remifentanil induces hyperalgesia in CFA mice.

(A) Schematic of the experimental procedure and behavioral tests.

(B and C) Time course assessment of mechanical pain threshold in ipsilateral hindpaws (**B**, $F_{(1,18)}$ = 134.3, P < 0.0001) and contralateral hindpaws (**C**, $F_{(1,18)}$ = 0.7554, P = 0.3962) of saline and CFA mice.

(D and E) Time course for thermal pain threshold assessment in ipsilateral hindpaws (**D**, $F_{(1,18)}$ = 18.14, P = 0.0005) and contralateral hindpaws (**E**, $F_{(1,18)}$ = 0.3494, P = 0.5618) of saline and CFA mice.

(F) Schematic of the experimental procedure and behavioral tests.

(G and H) Time course of mechanical pain threshold assessment in ipsilateral hindpaws (**G**, $F_{(1,18)}$ = 0.44, P = 0.5155) and contralateral hindpaws (**H**, $F_{(1,18)}$ = 39.59, P < 0.0001) of CFA mice infused with Remi (CFA + Remi) or saline (CFA + Remi).

(I and J) Time course of thermal pain threshold assessment in ipsilateral hindpaws (**I**, $F_{(1,18)}$ = 0.4050, P = 0.5325) and contralateral hindpaws (**J**, $F_{(1,18)}$ = 0.0484, P = 0.8284) of CFA mice infused with Remi (CFA + Remi) or saline (CFA + Remi).

Data: mean ± SEM. n = 10 mice per time point per group, **P < 0.01, ***P < 0.001. Two-way RM ANOVA with post hoc Bonferroni's test in (**B-E**) and (**G-J**).

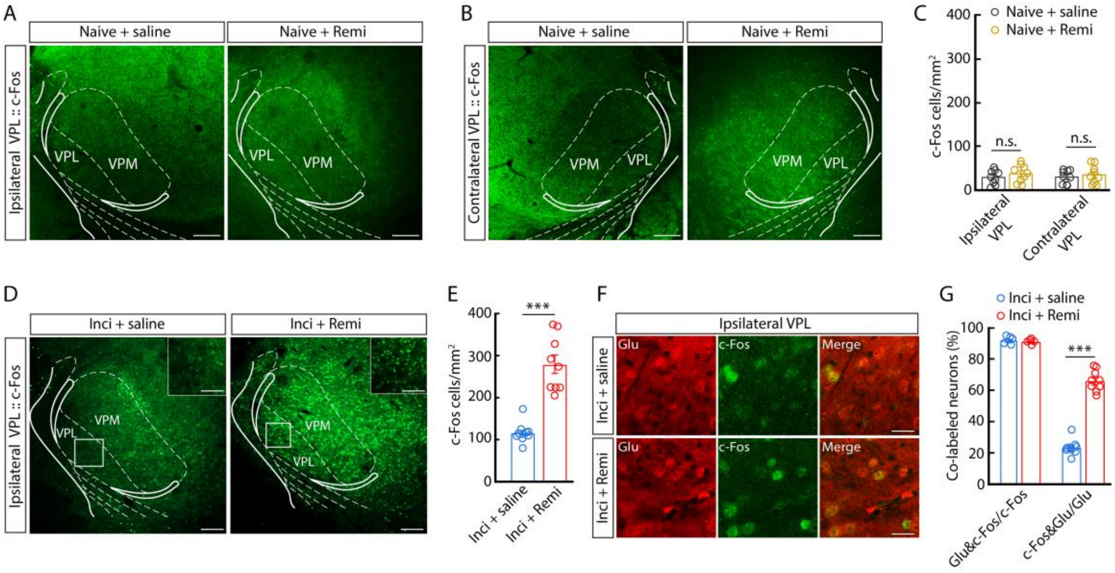

Supplemental Figure 3 | Expression of c-Fos in the VPL in naïve and RIH mice.

(A and B) Images showing the c-Fos immunofluorescence in ipsilateral (**A**) and contralateral (**B**) VPL from naïve mice treated with Remi or saline. Scale bars, 200 µm.

(C) Quantitative data showing the expression of c-Fos-positive neurons in ipsilateral (left, $t_{(16)} = 0.9502$, $P = 0.3561$) and contralateral (right, $t_{(16)} = 0.5589$, $P = 0.5839$) VPL in naïve mice treated with Remi or saline ($n = 9$ slices from 5 mice per group).

(D and E) Typical images (**D**) and summary data (**E**, $n = 9$ slices from 5 mice per group; $t_{(16)} = 6.98$, $P < 0.0001$) showing the expression of c-Fos in the ipsilateral VPL in mice with plantar incision infused with Remi or saline. Scale bars, 200 µm and 100 µm (enlargement).

(F) Images showing co-localization of c-Fos-positive neurons (green) with glutamate immunofluorescence (red). Scale bars, 20 µm.

(G) Summary data showing the percentage of c-Fos^{+} neurons expressing glutamate (left, $n = 5$ slices from 5 mice per group; $t_{(8)} = 0.7147$, $P = 0.4951$) and glutamate-positive neurons expressing c-Fos (right, $n = 9$ slices from 5 per group; $t_{(16)} = 15.22$, $P < 0.0001$) in the ipsilateral VPL of mice with plantar incision infused with Remi or saline.

Data: mean ± SEM. ***$P < 0.001$. Unpaired Student's t-test in (**C, F and G**).

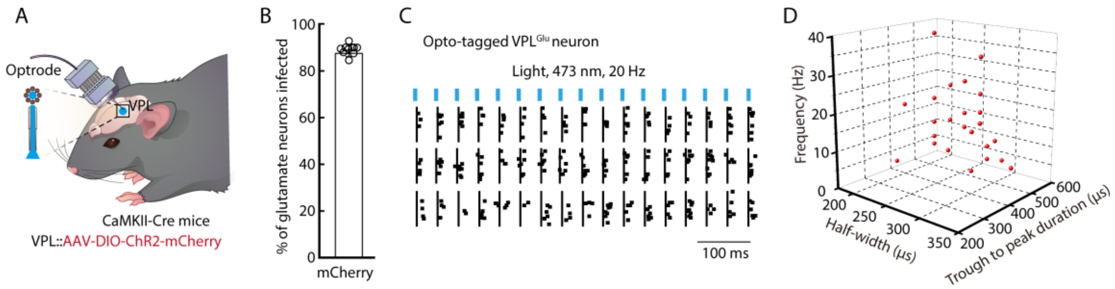

Supplemental Figure 4 | Optogenetic tagging of VPL^{Glu} neurons.

(A) Schematic diagram of optogenetic tagging and electrophysiological recording in the VPL of freely moving *CaMKII-Cre* mice. Enlargement showing optrodes.

(B) Quantitative data showing that Cherry^{+} neurons co-localized with glutamate immunofluorescence (n = 10 slices from 5 mice).

(C) Raster plot exhibiting spike responses to light stimuli at 20 Hz.

(D) Recorded light-sensitive neurons were classified as wide-spiking putative glutamatergic cells according to firing rate, half width and trough to peak duration of the spike.

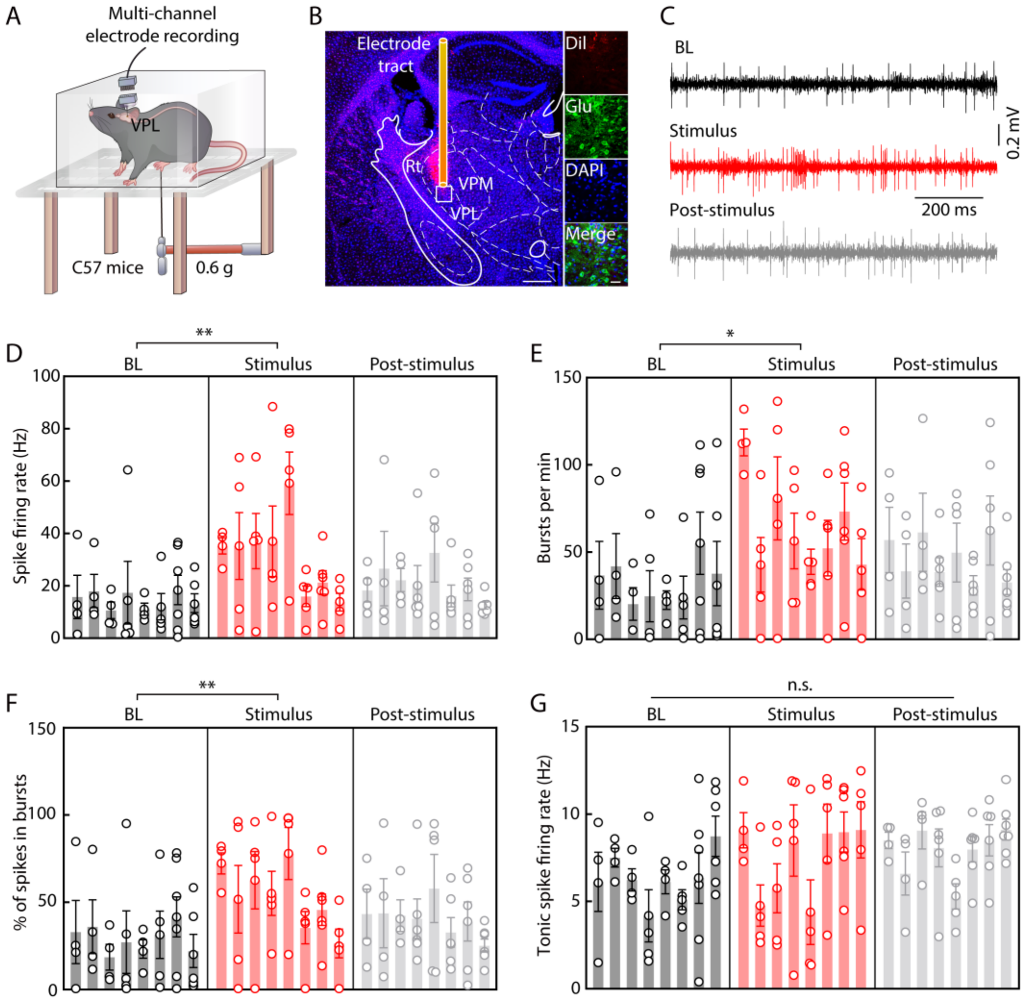

Supplemental Figure 5 | VPL^{Glu} neuronal response to noxious stimuli.

(A) Schematic illustration of multi-channel electrophysiological recording in the VPL of freely moving C57 mice.

(B) Representative images validating the site of the tetrode placement in the ipsilateral VPL (left), and depicting the overlap between DiI (red) and anti-glutamate-positive neurons (green, right). Scale bars, 1 mm (left) and 20 μm (right).

(C) Representative traces of spontaneous spikes recorded from ipsilateral VPL^{Glu} neurons of C57 mice before, during, and after the noxious stimuli (von Frey filament, 0.6 g) on the right hindpaws.

(D-G) Comparison of total spike firing rate (**D**, n = 8 mice; $F_{(2,21)}$ = 6.388, P = 0.0068), burst number/min (**E**, n = 8 mice; $F_{(2,21)}$ = 5.559, P = 0.0115), percentage of spikes in bursts (**F**, $F_{(2,21)}$ = 6.55, P = 0.0062) and tonic spike firing (**G**, $F_{(2,21)}$ = 1.988, P = 0.162) recorded from ipsilateral VPL^{Glu} neurons of C57 mice (n = 8 mice).

Data: mean ± SEM. *P < 0.05, **P < 0.01, n.s., not significant. Nested one-way ANOVA with post hoc with post hoc Bonferroni's test in (**D-G**).

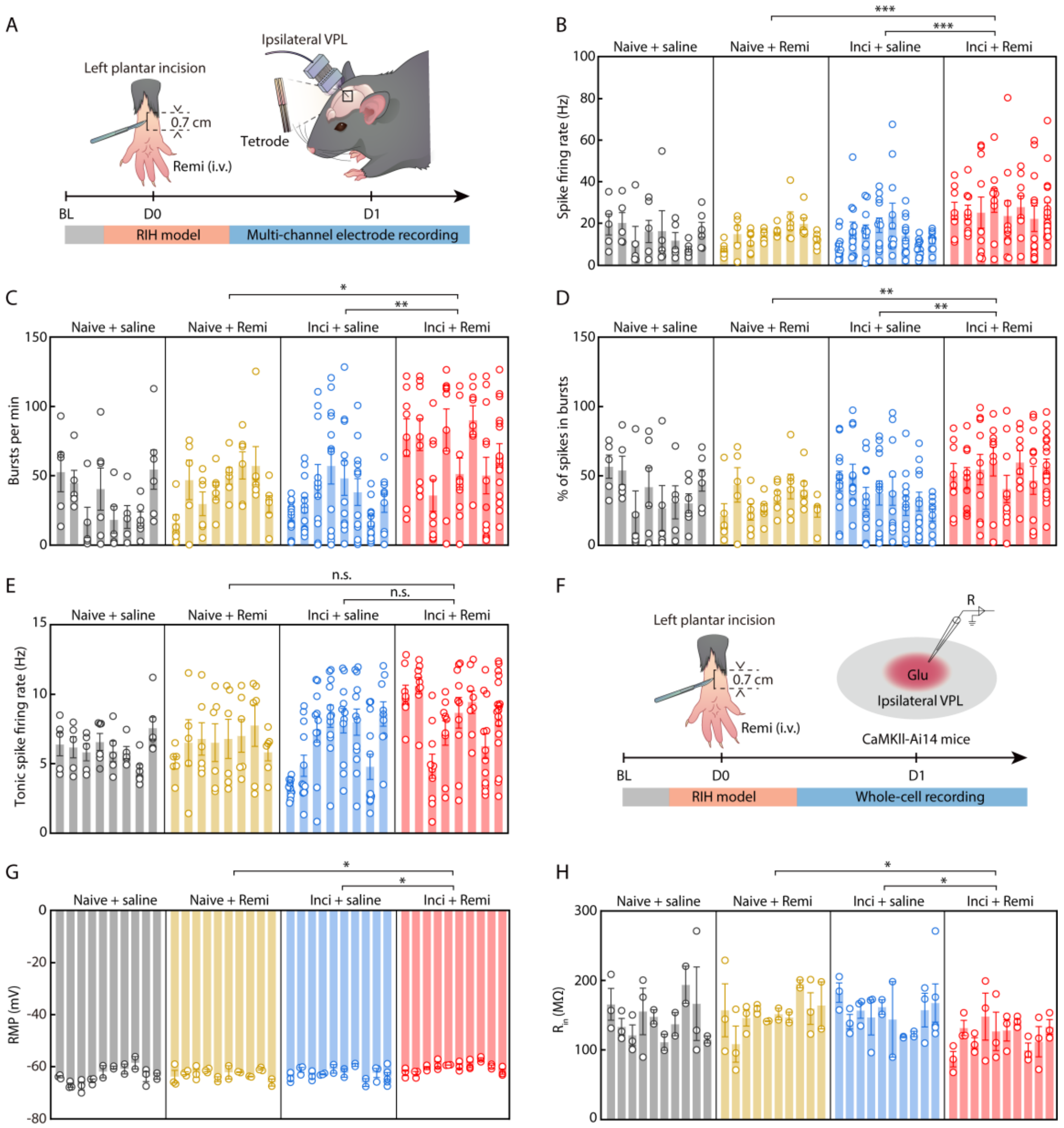

Supplemental Figure 6 | Enhanced neuronal excitability of ipsilateral VPL^{Glu} neurons in RIH mice on postoperative day 1.

(A) Schematic diagram of multi-channel electrophysiological recordings in ipsilateral VPL^{Glu} neurons of RIH mice on postoperative day 1.

(B-E) Comparison of total spike firing rate (**B**, $F_{(3,260)}$ = 12.72, P < 0.0001), burst number/min (**C**, $F_{(3,28)}$ = 7.063, P = 0.0011), percentage of spikes in bursts (**D**, $F_{(3,28)}$ = 7.775, P = 0.0006) and tonic spike firing (**E**, $F_{(3,28)}$ = 2.363, P = 0.0926) recorded in ipsilateral VPL^{Glu} neurons of C57 mice from each group on postoperative day 1 (n = 8 mice per group; nested one-way ANOVA test).

(F) Schematic diagram of whole-cell recordings.

(G and H) Quantification of the resting membrane potential (RMP) (**G**, $F_{(3,36)}$ = 5.223, P = 0.0043) and input resistance (R_{in}) (**H**, $F_{(3,104)}$ = 4.423, P = 0.0057) recorded in ipsilateral VPL^{Glu} neurons (n = 25-30 neurons from 10 mice per group).

Data: mean ± SEM. *$P < 0.05$, **$P < 0.01$, ***$P < 0.001$, n.s., not significant. Nested one-way ANOVA with post hoc Bonferroni's test in (**B-E**), **(G) and (H**).

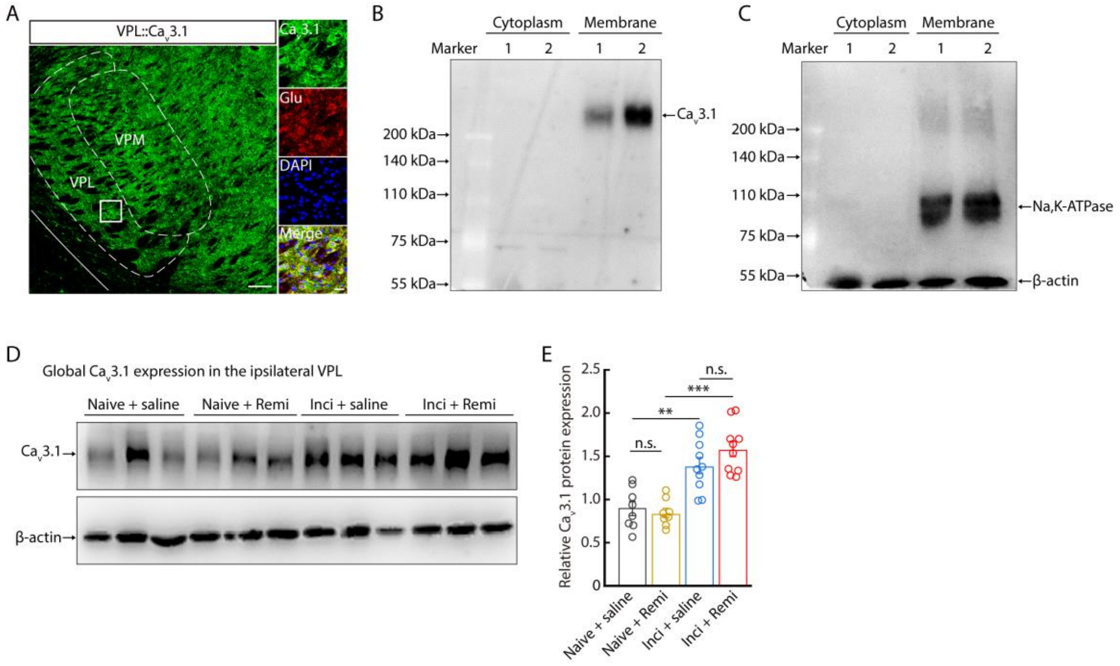

Supplemental Figure 7 | Increased global expression of $Ca_v3.1$ channels in ipsilateral VPL of RIH mice.

(A) Representative images showing $Ca_v3.1$ (green) expression in neurons with co-labeling of glutamate immunofluorescence (red) in the VPL. Scale bars, 200 μm (left) and 20 μm (right).

(B) The whole PVDF-membrane from Western blots of $Ca_v3.1$ expression in the cytoplasmic and cell membrane fractions prepared from VPL tissue.

(C) The whole PVDF-membrane from Western blots of Na,K-ATPase and β-actin in VPL tissue.

(D and E) Western blot analysis of global $Ca_v3.1$ expression in the ipsilateral VPL of mice from each groups (n = 8-10 mice per group; $F_{(3,32)} = 18.74$, $P < 0.0001$, one-way ANOVA with post hoc Bonferroni's test).

Data: mean ± SEM. $^{**}P < 0.01$, $^{***}P < 0.001$, n.s., not significant.

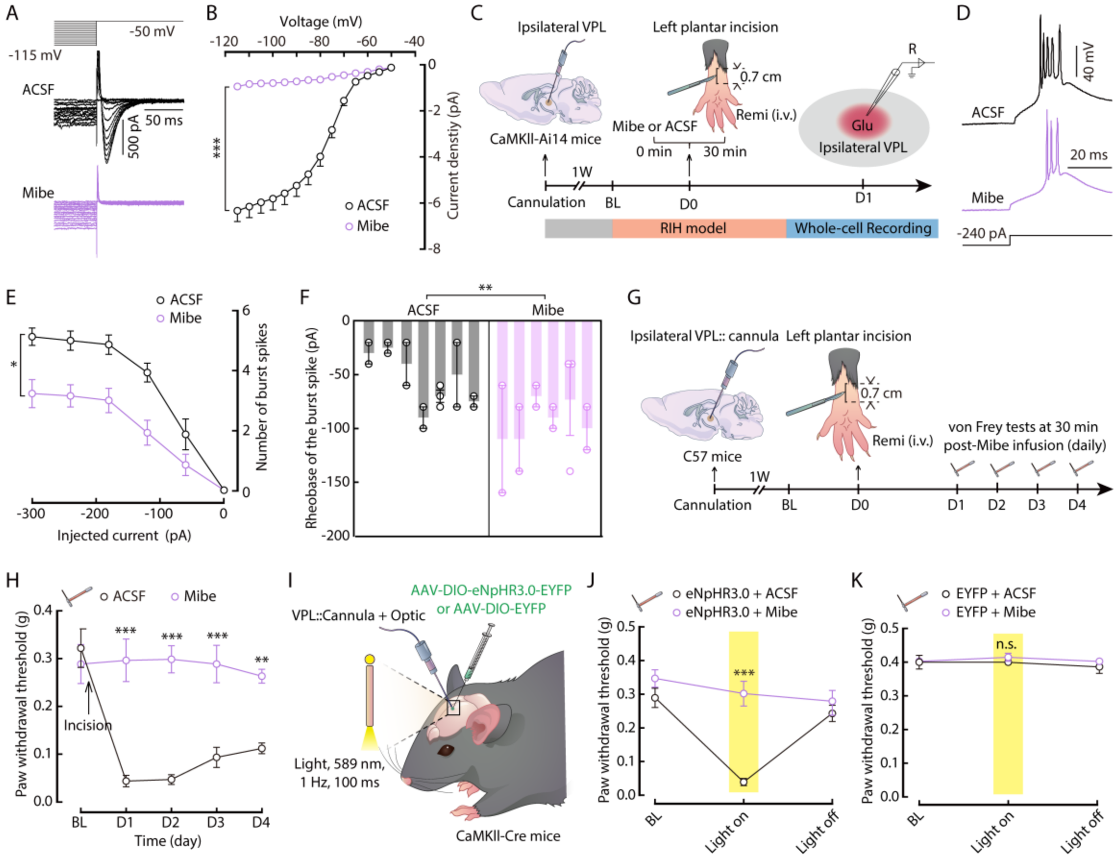

Supplemental Figure 8 | Antagonizing T-type calcium channels blocks burst firing and relieves RIH.

(A) Representative traces of T-type calcium currents in the presence of ACSF or Mibe from a VPL^{Glu} neuron.

(B) Current–voltage (*I*–*V*) curves of T-type calcium current density in the presence of ACSF or Mibe of VPL^{Glu} neurons (n = 13 neurons from 6 mice per group; $F_{(1,143.65)} = 33.348$, $P < 0.0001$).

(C) Schematic of whole-cell recordings in ipsilateral VPL^{Glu} neurons of RIH *CaMKII-Ai14* mice injected with Mibe or ACSF in the ipsilateral VPL.

(D and E) Representative traces (**D**) of quantitative data (**E**) of hyperpolarized current-induced burst firing recorded from ipsilateral VPL^{Glu} neurons in the presence of ACSF or Mibe (n = 15 neurons from 7 ACSF mice; n = 13 neurons from 6 Mibe mice; $F_{(1,138)} = 17.244$, $P = 0.015$;).

(F) Quantitative data of the rheobase of the burst spike recorded from ipsilateral VPL^{Glu} neurons in the presence of ACSF or Mibe (n = 15 neurons from 7 ACSF mice; n = 13 neurons from 6 Mibe mice; $t_{(26)} = 2.807$, $P = 0.0094$).

(G) Schematic of the experimental procedure for ipsilateral VPL injection with Mibe or ACSF in RIH model mice.

(H) Quantitative data showing significant relief of postoperative hyperalgesia by ipsilateral VPL infusion of Mibe in RIH mice (n = 9 mice per group; $F_{(1,16)} = 48.92$, $P < 0.0001$).

(I) Schematic of the cannula and optic fiber implantation.

(J) Quantitation of the effects of pre-administration of Mibe or ACSF on pain sensitization induced by yellow light stimulation of eNpHR3.0-EYFP expressing VPL^{Glu} neurons in the *CaMKII-Cre* mice (n = 8 mice per group; $F_{(1,14)} = 26.49$, $P < 0.0001$).

(K) Quantitation showing the effects of pre-administration of Mibe or ACSF on the pain sensitizations induced by yellow light stimulation of EYFP expressing VPL^{Glu} neurons in the *CaMKII-Cre* mice (n = 7 mice per group; $F_{(1,12)} = 3.439$, $P = 0.0884$).

Data: mean ± SEM. $^{**}P < 0.01$, $^{***}P < 0.001$, n.s., not significant. Linear mixed models with post hoc Bonferroni's test in (**B)** and **(E**); nested *t*-test in (**F**); two-way RM ANOVA with post hoc Bonferroni's test in (**H), (J)** and **(K**).

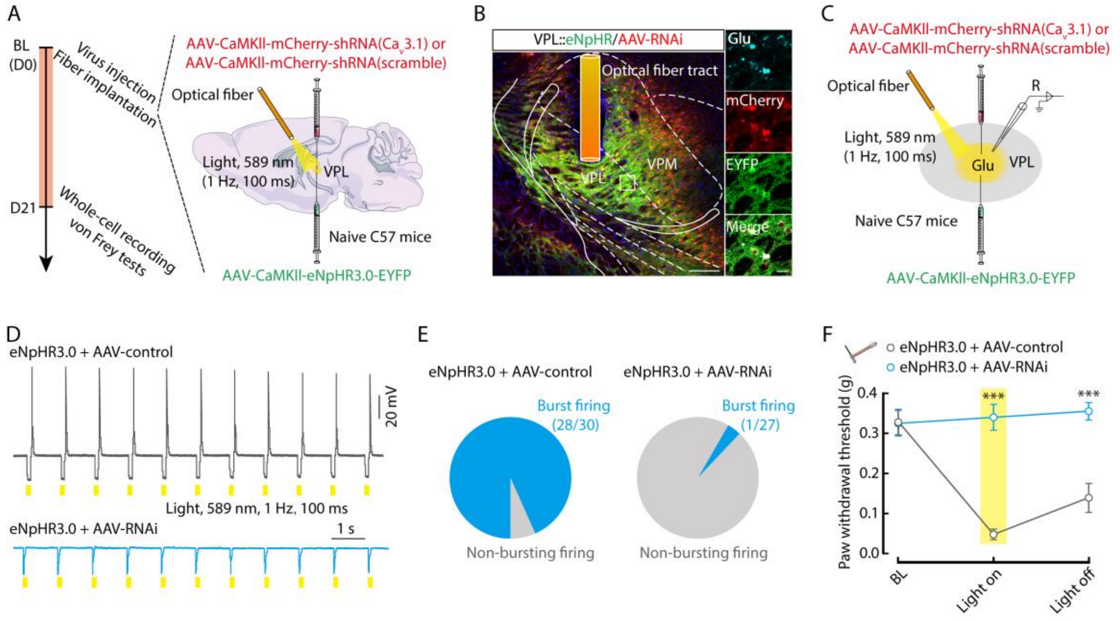

Supplemental Figure 9 | Knockdown of $Ca_v3.1$ in VPL^{Glu} neurons reverses yellow light-induced pain sensitization in naïve mice.

(A) Schematic of VPL injection with AAV-CaMKII-eNpHR3.0-EYFP and AAV-CaMKII-mCherry-shRNA ($Ca_v3.1$) (AAV-RNAi) or AAV-CaMKII-mCherry-shRNA(scramble) (AAV-control) in naïve C57 mice.

(B) Representative images of AAV-CaMKII-eNpHR3.0-EYFP and AAV-CaMKII-mCherry-shRNA ($Ca_v3.1$) virus expression in the VPL of C57 mice (left); eNpHR3.0-EYFP and mCherry-shRNA co-labeled neurons co-localized with glutamate (Glu) immunofluorescence signal (right). Scale bars, 200 μm (left) and 20 μm (right).

(C) Schematic diagram of VPL virus injection and recording configuration with yellow light stimulation in acute brain slices.

(D) Representative traces of burst firing induced by yellow light optostimulation in VPL^{Glu} neurons co-expressing eNpHR3.0 + AAV-control or eNpHR3.0 + AAV-RNAi.

(E) The proportion of burst firing neurons in the eNpHR3.0 + AAV-RNAi group (1/27) and the eNpHR3.0 + AAV-control group (28/30).

(F) Quantitative data from mechanical pain threshold determination in each group (eNpHR3.0 + AAV-control, n = 10 mice; eNpHR3.0 + AAV-RNAi, n = 8 mice; $F_{(1,16)} = 28.16$, $P < 0.0001$; Two-way RM ANOVA with post hoc Bonferroni's test).

Data: mean ± SEM. $^{***}P < 0.001$, n.s., not significant.

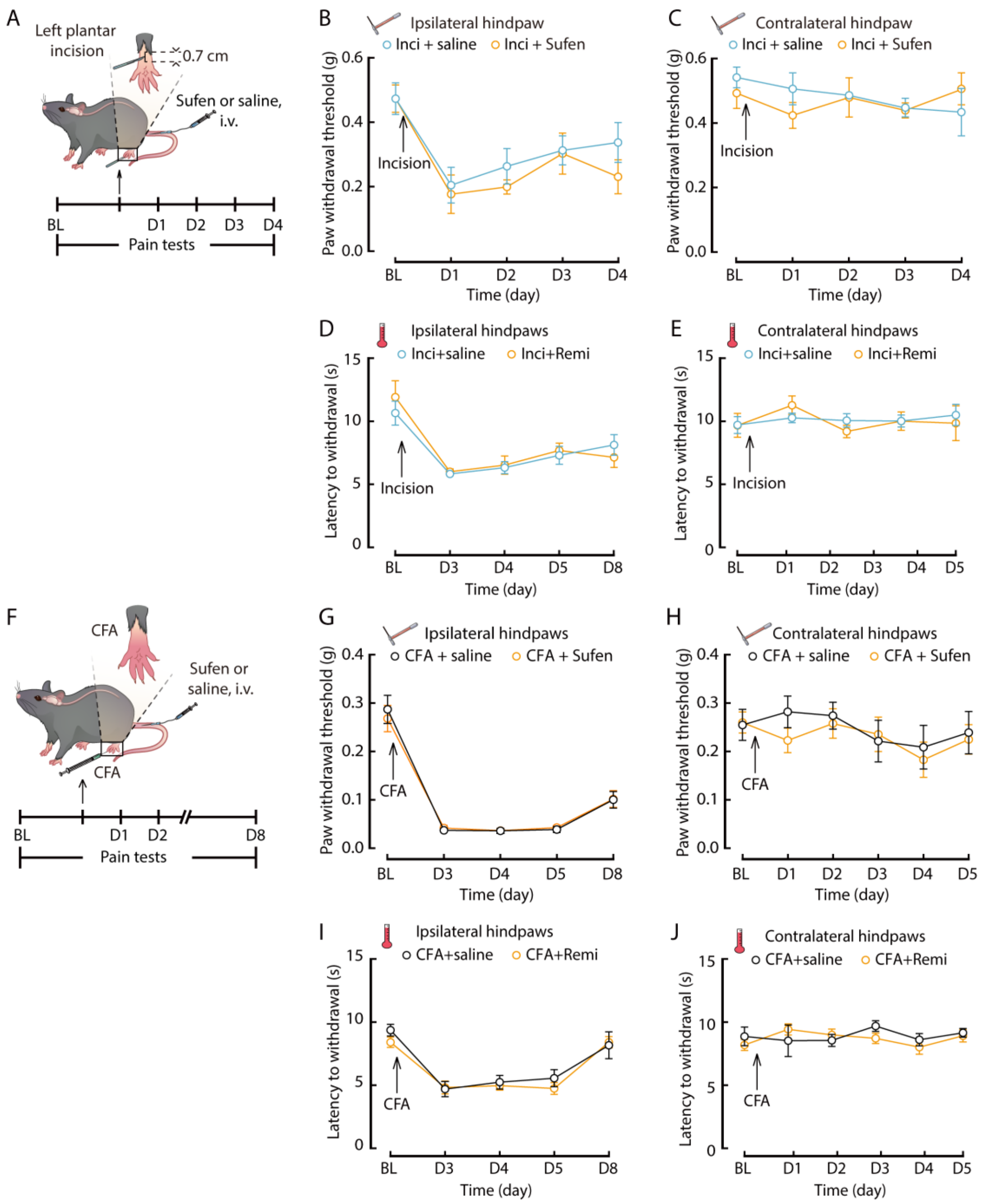

Supplemental Figure 10 | Intraoperative infusion of sufentanil does not induce post-operative hyperalgesia in incisional and CFA mice.

(A) Schematic of the experimental procedure for mice with plantar incision infused with sufentanil (Sufen) or saline via tail vein and behavioral tests.

(B and C) Time course assessment of mechanical pain thresholds in ipsilateral hindpaws (**B**, $F_{(1,10)}$ = 1.119, P = 0.3149) and contralateral hindpaws (**C**, $F_{(1,10)}$ = 0.2315, P = 0.6408) of incisional mice infused with sufentanil (Inci + Sufen) or saline (Inci + saline) (n = 6 mice per group).

(D and E) Time course assessment of thermal pain threshold in ipsilateral hindpaws (**D**, $F_{(1,18)}$ = 0.2962, P = 0.5929) and contralateral hindpaws (**E**, $F_{(1,18)}$ = 0.0604, P = 0.8085) of incisional mice infused with Sufen or saline (n = 10 mice per time point per group).

(F) Schematic of the experimental procedure for CFA mice with Sufen or saline infusion (i.v.) and behavioral tests.

(G and H) Time course of mechanical pain threshold assessment in ipsilateral hindpaws (**G**, $F_{(1,18)}$ = 0.0231, P = 0.8808) and contralateral hindpaws (**H**, $F_{(1,18)}$ = 0.6835, P = 0.4192) of CFA mice infused with Sufen (CFA + Sufen) or saline (CFA + saline) (n = 10 mice per time point per group).

(I and J) Time course of thermal pain threshold assessment in ipsilateral hindpaws (**I**, $F_{(1,18)}$ = 0.7234, P = 0.4062) and contralateral hindpaws (**J**, $F_{(1,18)}$ = 0.2171, P = 0.6469) of CFA mice infused with Sufen or saline (n = 10 mice per time point per group).

Data: mean ± SEM. Two-way RM ANOVA with post hoc Bonferroni's test in (**B-E**) and (**G-J**).

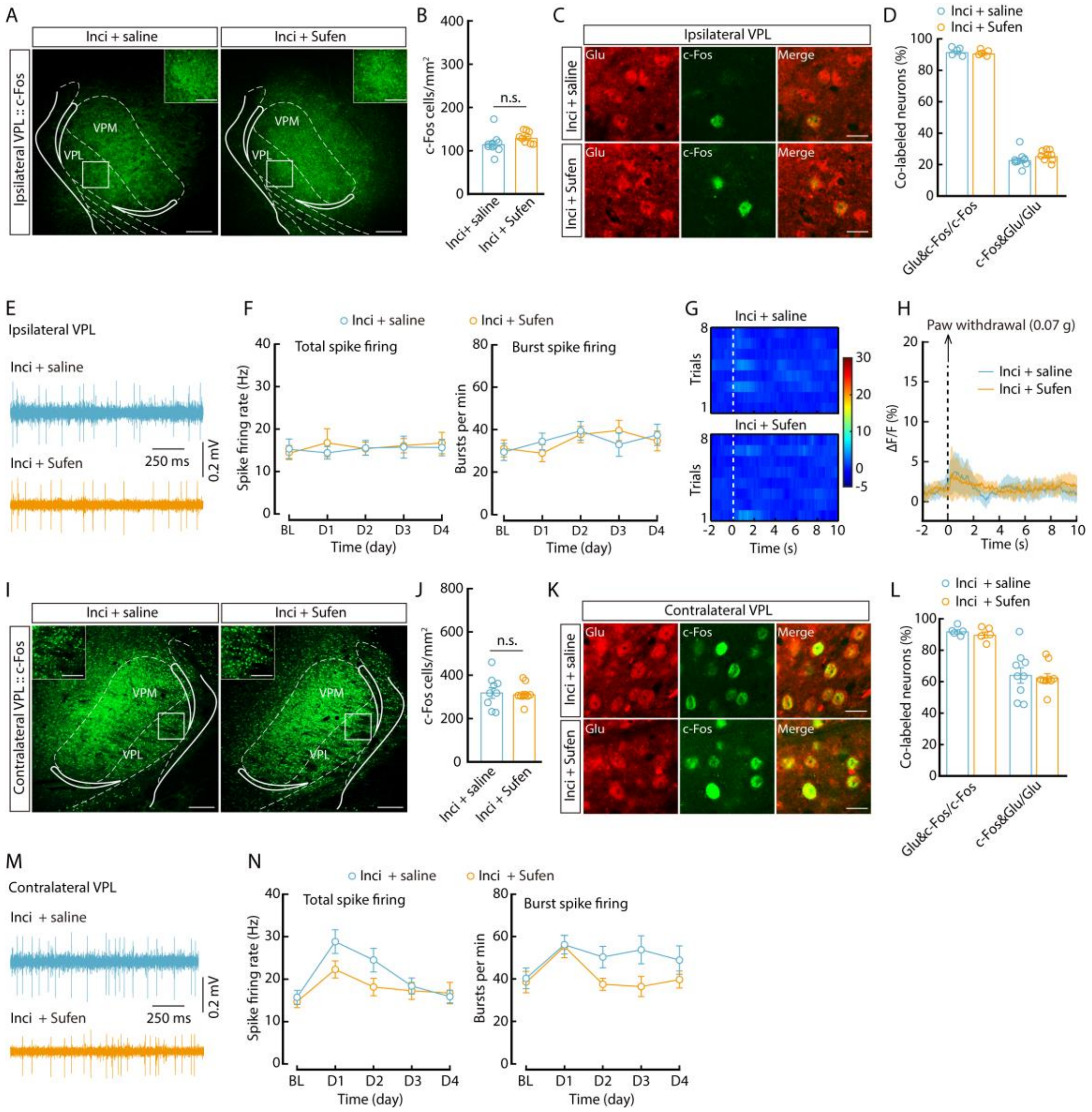

Supplemental Figure 11 | No change in bilateral VPL^{Glu} neuronal activity associated with sufentanil infusion in incisional mice.

(A and B) Typical images (**A**) and summary data (**B,** n = 9 slices from 5 mice per group; $t_{(16)}$ = 1.583, P = 0.1331) showing the expression of c-Fos in the ipsilateral VPL in mice with plantar incision infused with Sufen or saline. Scale bars, 200 μm and 100 μm (enlargement).

(C) Images showing co-localization of c-Fos-positive neurons with glutamate immunofluorescence. Scale bars, 20 μm.

(D) The percentage of c-Fos^{+} neurons expressing glutamate (left, n = 5 slices from 5 mice per group; $t_{(8)}$ = 0.6858, P = 0.5122) and glutamate-positive neurons expressing c-Fos (right, n = 9 slices from 5 mice per group; $t_{(16)}$ = 1.265, P = 0.2241) in the ipsilateral VPL of mice.

(E) Example traces of the spike firing recorded from ipsilateral VPL^{Glu} neurons in Inci + saline and Inci + Sufen mice on postoperative day 1.

(F) Quantitative data of total spike firing rate (left, $F_{(1,727)}$ = 0.026, P = 0.974), and burst number/min (right, $F_{(1,727)}$ = 0.034, P = 0.917) of ipsilateral VPL^{Glu} neurons recorded from mice

of two groups (n = 54-78 neurons from 8 mice per group).

(G and H) Heatmaps (**G**) and the mean data (**H**) showing $Glu^{GCaMP6m}$ signals recorded from ipsilateral VPL^{Glu} neurons in mice of two groups after subthreshold stimuli. Color scale at the right in (**G**) indicates ΔF/F (%).

(I and **J)** Typical images (**I**) and quantitative data (**J**, n = 9 slices from 5 mice per group; $t_{(16)}$ = 0.2964, P = 0.7707) showing the expression of c-Fos in the contralateral VPL in Inci + saline and Inci + Sufen mice. Scale bars, 200 μm and 100 μm (enlargement).

(K) Images showing co-localization of c-Fos-positive neurons (green) with glutamate immunofluorescence (red) in the contralateral VPL of mice with plantar incision infused with Sufen or saline. Scale bars, 20 μm.

(L) Summary data showing the percentage of $c\text{-}Fos^{+}$ neurons expressing glutamate (left, n = 5 slices from 5 mice per group; $t_{(8)}$ = 0.7499, P = 0.4748) and glutamate-positive neurons expressing c-Fos (right, n = 9 slices from 5 mice per group; $t_{(16)}$ = 0.2588, P = 0.7991) in the contralateral VPL of incisional mice treat with saline or Sufen.

(M) Example traces of the spike firing recorded from contralateral VPL^{Glu} neurons in mice from two groups.

(N) Quantitative data of total spike firing rate (left, $F_{(1,623.5)}$ = 3.349, P = 0.732), and burst number/min (right, $F_{(1,623.5)}$ = 4.155, P = 0.604) of contralateral VPL^{Glu} neurons recorded from mice of two groups (n = 56-70 neurons from 8 mice per group).

Data: mean ± SEM. Unpaired Student's t-test in (**B**), (**D**), (**J**) and (**L**); linear mixed models with post hoc Bonferroni's test in (**F**) and (**N**).

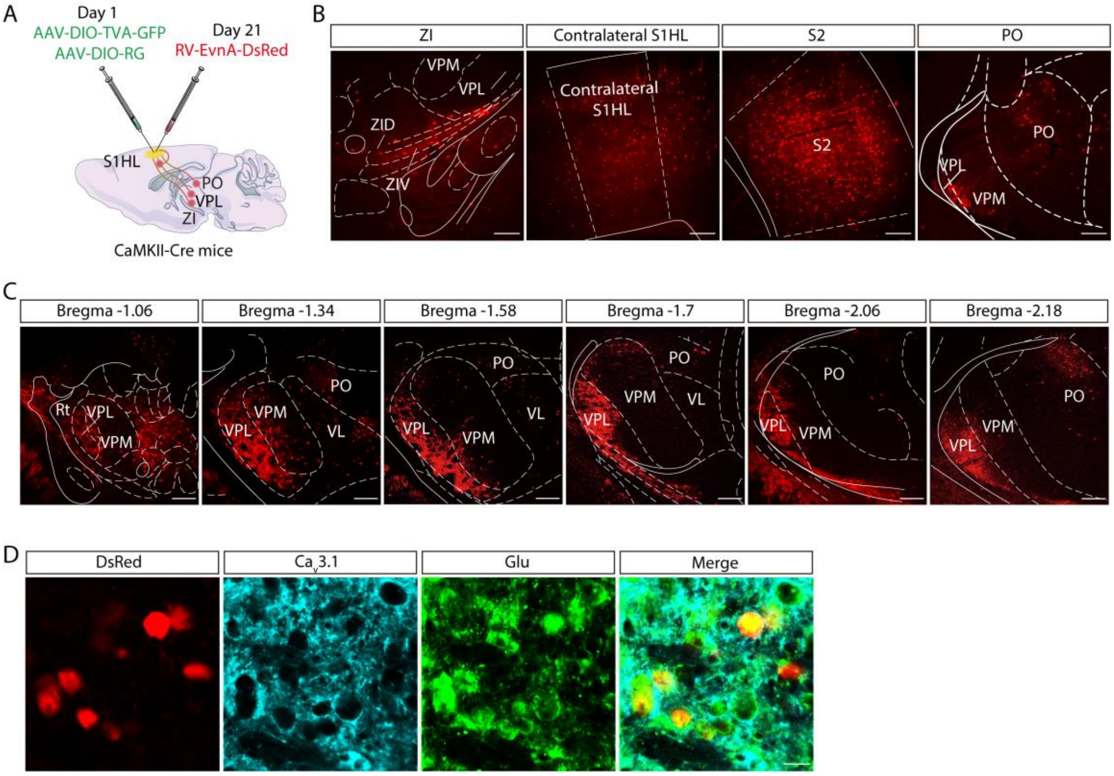

Supplemental Figure 12 | The VPL^{Glu} neurons project onto S1HL^{Glu} neurons.

(A) Schematic of the Cre-dependent retrograde trans-monosynaptic rabies virus tracing strategy in S1HL^{Glu} neurons of *CaMKII-Cre* mice.

(B) Typical images showing DsRed-labeled neurons within the ZI, contralateral S1HL, S2 and PO traced from the ipsilateral S1HL. Scale bars, 200 μm.

(C) DsRed-labeled neurons expressions at different bregma sites (from −1.06 to −2.18) in the VPL of *CaMKII-Cre* mice. Scale bars, 200 μm.

(D) Co-labeling of $Ca_v3.1$ channels (blue) with DsRed-labeled (red) glutamatergic neurons (green) in the VPL. Scale bar, 20 μm.

ZI, insular cortex; S2, secondary somatosensory cortex; VPM, ventral posteromedial thalamic nucleus; PO, posterior thalamic nucleus; VL, ventrolateral thalamic nucleus.

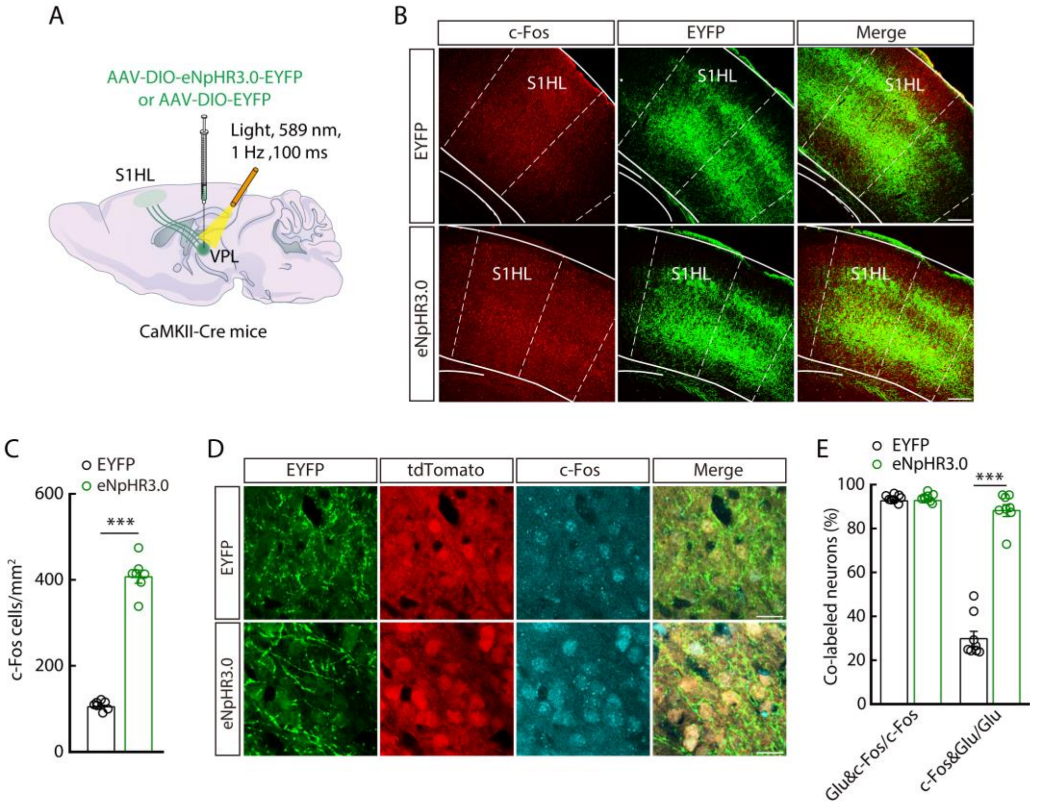

Supplemental Figure 13 | eNpHR3.0 induced burst firing in the VPL^{Glu} neurons increases the expression of c-Fos in S1HL^{Glu} neurons.

(A) Schematic of yellow light stimuli in the VPL injected with AAV-DIO-eNpHR3.0-EYFP or AAV-DIO-EYFP in *CaMKII-Cre* mice.

(B and C) Representative images (**B**) and quantitative data (**C**, n = 7 slices from 5 mice per group; $t_{(12)}$ = 19.16, P < 0.0001) showing the expression of c-Fos (red) in the ipsilateral S1HL after yellow light stimuli in the VPL^{Glu} neurons expressing eNpHR3.0-EYFP or EYFP. Scale bars, 200 μm.

(D) Images showing co-localization of c-Fos-positive neurons (blue) with tdTomato^{+} glutamatergic neurons (red) in the S1HL after yellow light stimuli in the VPL^{Glu} neurons expressing eNpHR3.0-EYFP or EYFP in *CaMKII-Ai14* mice. Scale bars, 20 μm.

(E) Summary data showing the percentage of c-Fos^{+} neurons expressing glutamate (left, $t_{(14)}$ = 0.0604, P = 0.9526) and glutamate-positive neurons expressing c-Fos (right, $t_{(14)}$ = 13.12, P < 0.0001) after yellow light stimuli in the VPL^{Glu} neurons expressing eNpHR3.0-EYFP or EYFP in *CaMKII-Ai14* mice (n = 8 slices from 5 mice per group).

Data: mean ± SEM. ***P < 0.001. Unpaired Student's t-test in (**C**) and (**E**).

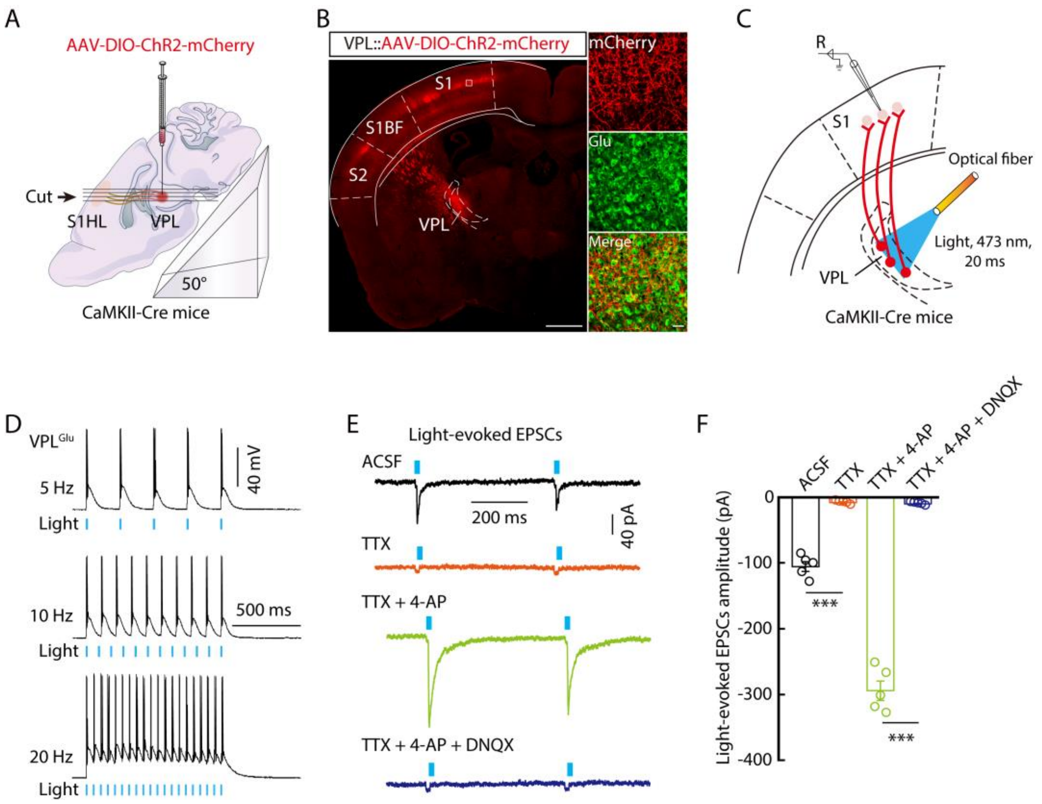

Supplemental Figure 14 | Excitatory monosynaptic projections from VPL^{Glu} neurons to S1HL^{Glu} neurons.

(A) Schematic representation of thalamocortical somatosensory slices of *CaMKII-Cre* mice with infusion of AAV-DIO-ChR2-mCherry into the VPL.

(B) Image representative of mCherry^{+} fibers (red) in thalamocortical somatosensory slices (left); these mCherry^{+} fibers wrap neurons in the S1HL co-localized with the glutamate antibody signals (green, right). Scale bars, 1 mm (left) and 20 μm (right).

(C) Schematic diagram showing pulsed blue light stimulation (473 nm, 2 Hz, 20 ms) of ChR2-mCherry^{+} neurons within the VPL of *CaMKII-Cre* mice and recording configuration in the S1HL in acute thalamocortical brain slices.

(D) Representative traces of light-evoked action potentials recorded from ChR2-expressed VPL^{Glu} neurons in the thalamocortical slices.

(E and F) Representative traces (**E**) and summarized data (**F**, n = 5 neurons from 5 mice; $F_{(3,16)} = 262.1$, $P < 0.0001$, one-way ANOVA with post hoc Bonferroni's test) showing light-evoked EPSCs recorded from ipsilateral S1HL^{Glu} neurons held at −70 mV in the thalamocortical slices under the recording configuration in (**C**). These EPSCs were blocked by bath application of TTX and could be rescued by bath application of the potassium channel blocker 4-AP, which were eliminated by the AMPA receptor antagonist DNQX.

TTX, tetrodotoxin; 4-AP, 4-aminopyridine; DNQX, 6,7-Dinitroquinoxaline-2,3(1H,4H)-dione.
Data: mean ± SEM. ***$P < 0.001$.

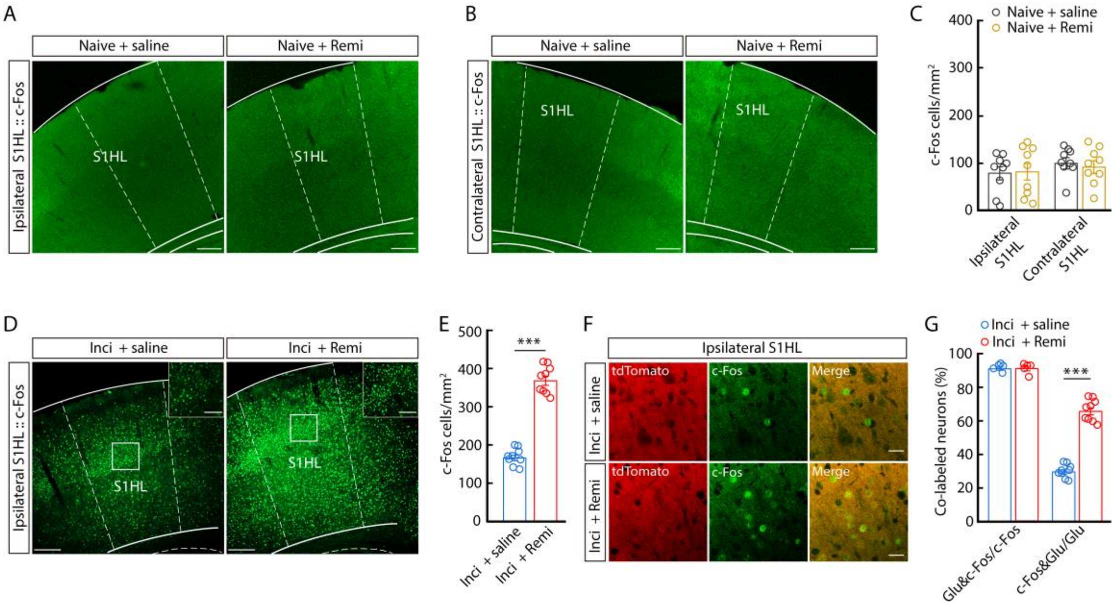

Supplemental Figure 15 | Increased c-Fos expression in the S1HL of RIH mice.

(A and B) Images showing the expression of c-Fos in the ipsilateral (**A**) and contralateral (**B**) S1HL from naïve mice treated with Remi or saline. Scale bars, 200 μm.

(C) Quantitative data showing the expression of c-Fos-positive neurons in ipsilateral (left, $t_{(16)} = 0.1356$, $P = 0.8939$) and contralateral ($t_{(16)} = 0.4431$, $P = 0.6636$) S1HL in naïve mice treated with Remi or saline ($n = 9$ slices from 5 mice per group).

(D and E) Images (**D**) and summary data (**E**, $n = 9$ slices from 5 mice per group; $t_{(16)} = 14.34$, $P < 0.0001$) showing the expression of c-Fos in the ipsilateral S1HL of *CaMKII-Ai14* mice with plantar incision infused with Remi or saline. Scale bars, 200 μm and 100 μm (enlargement).

(F) Images showing co-localization of c-Fos-positive neurons (green) with $tdTomato^{+}$ glutamatergic neurons (red). Scale bars, 20 μm.

(G) Summary data showing the percentage of $c\text{-}Fos^{+}$ neurons expressing glutamate (left, $n = 5$ slices from 5 mice per group; $t_{(8)} = 0.3101$, $P = 0.7644$) and glutamate-labeled neurons expressing c-Fos ($n = 9$ slices from 5 mice per group; $t_{(16)} = 14.32$, $P < 0.0001$) in the ipsilateral S1HL of *CaMKII-Ai14* mice with plantar incision infused with Remi or saline.

Data: mean ± SEM. ***$P < 0.001$, n.s., not significant. Unpaired Student's *t*-test in (**C**), (**E**) and (**G**).

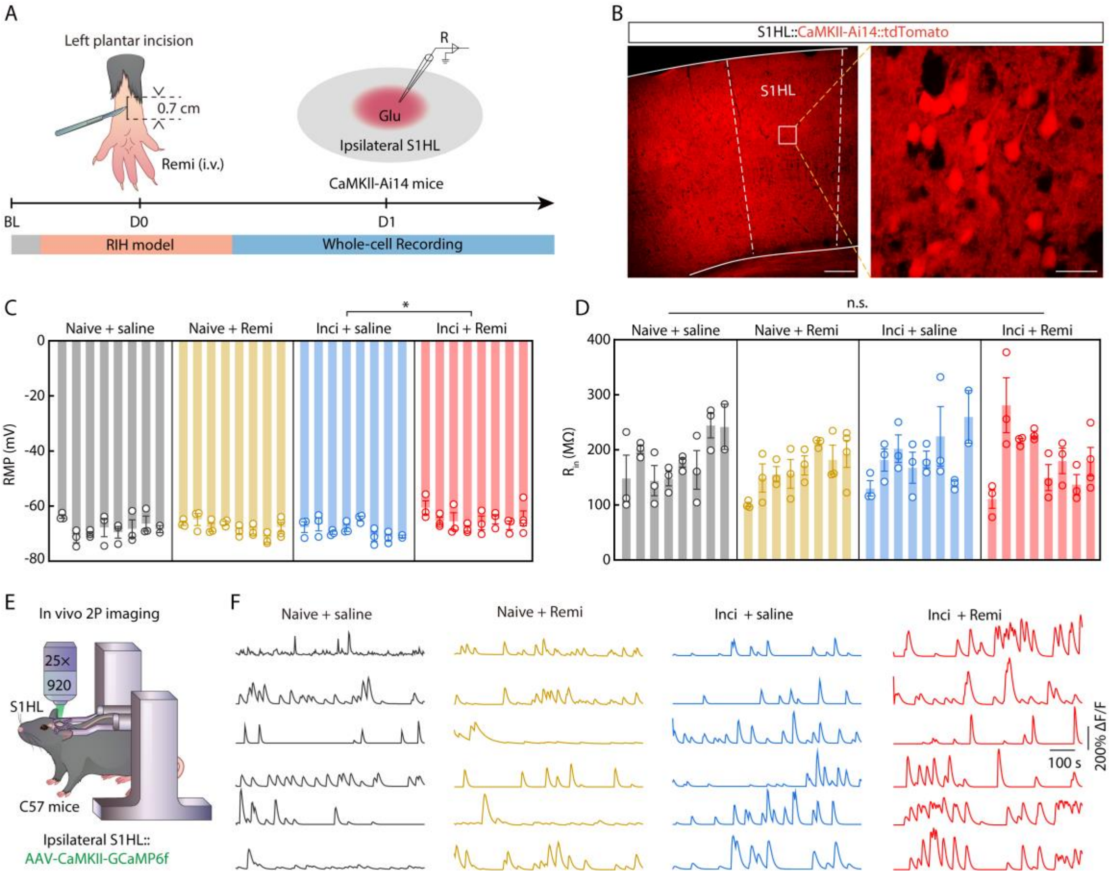

Supplemental Figure 16 | Enhanced activity in S1HL^{Glu} neurons of RIH mice.

(A) Schematic of recording configuration in ipsilateral tdTomato^{+} S1HL^{Glu} neurons in brain slices from *CaMKII-Ai14* mice.

(B) Images showing the tdTomato^{+} signals in the S1HL^{Glu} neurons *CaMKII-Ai14* mice. Scale bars, 200 μm and 20 μm (enlargement).

(C and D) Quantitative data of the RMP (**C**, $F_{(3,28)}$ = 3.609, P = 0.0254) and R_{in} (**D**, $F_{(3,28)}$ = 0.3751, P = 0.7716) recorded from ipsilateral S1HL^{Glu} neurons in mice of four groups (n = 23-25 neurons from 8 mice per group; nested one-way ANOVA with post hoc Bonferroni's test).

(E) Schematic illustration of ipsilateral S1HL^{Glu} neurons injected of AAV-CaMKII-GCaMP6f-GFP in C57 mice for in vivo 2P imaging.

(F) Spontaneous ΔF/F time series traces in GCaMP6^{+} ipsilateral S1HL^{Glu} neurons of mice from four groups.

Data: mean ± SEM. *P < 0.05, n.s., not significant.

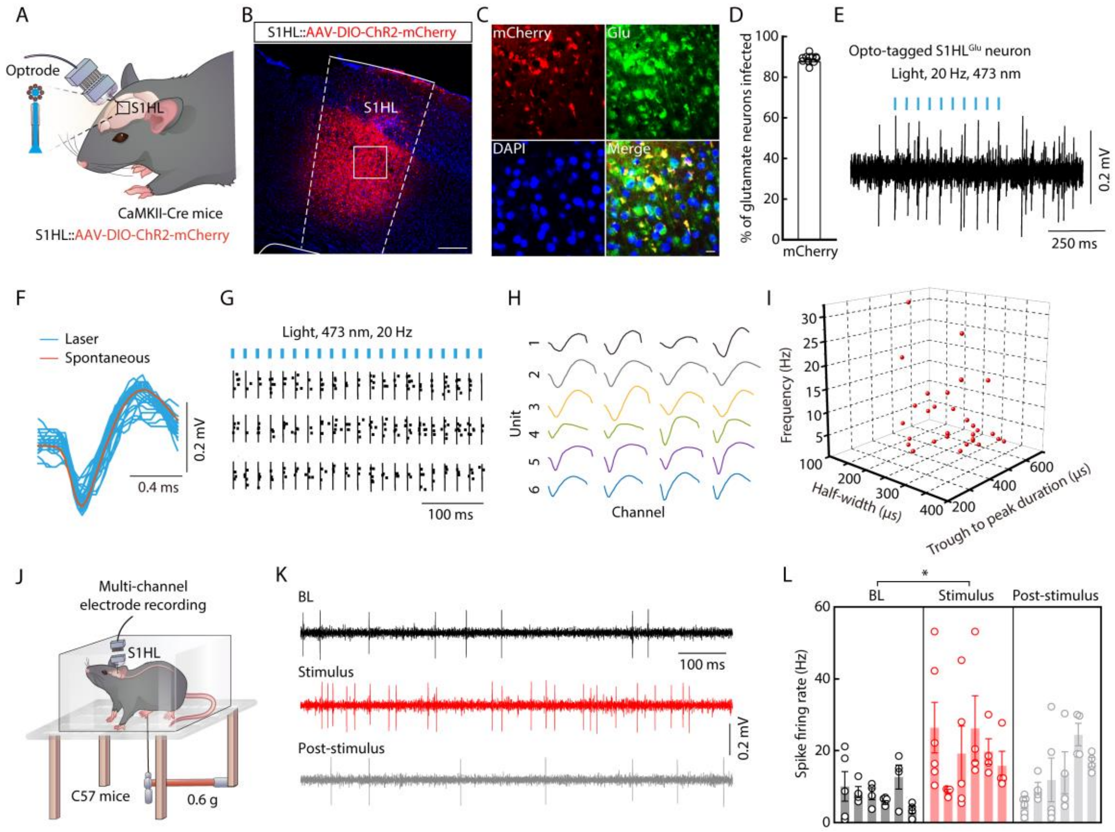

Supplemental Figure 17 | Optogenetic tagging of S1HL^{Glu} neurons involved in the processing of noxious stimuli.

(A) Schematic diagram of optogenetic tagging and electrophysiological recording in the S1HL of freely moving *CaMKII-Cre* mice with the S1HL infusion of AAV-DIO-ChR2-mCherry. Enlargement showing optrodes.

(B) Representative images of AAV-DIO-ChR2-mCherry injected site of S1HL. Scale bar, 200 μm

(C and D) Images **(C)** and summary data **(D**, n = 10 slices from 5 mice**)** showing that Cherry^{+} neurons co-localized with glutamate immunofluorescence. Scale bar, 20 μm.

(E) Example recording of spontaneous and light-evoked (473 nm, 20 Hz) spikes from a S1HL^{Glu} neuron.

(F) Overlay of averaged spontaneous (blue) and light-evoked (red) spike waveforms from the example unit.

(G) Raster plot exhibiting spike responses to light stimuli at 20 Hz.

(H) Average spike waveform sorting results of wide-spiking putative glutamatergic pyramidal neurons recorded through a single tetrode in the S1HL.

(I) Recorded light-sensitive neurons were classified as wide-spiking putative glutamatergic pyramidal neurons according to firing rate, half width and trough to peak duration of the spike.

(J) Schematic illustration of multi-channel electrophysiological recording in the left S1HL of freely moving C57 mice while noxious mechanical stimuli (von Frey filament, 0.6 g) were

delivered to the right hindpaws.

(K) Representative traces of spontaneous spikes of left S1HL^{Glu} neurons in C57 mice before, during, and after the noxious stimuli on the right hindpaws.

(L) Comparison of spike firing rate recorded from right S1HL^{Glu} neurons (n = 6 mice; $F_{(2,21)}$ = 6.388, P = 0.0068, nested one-way ANOVA with post hoc Bonferroni's test) .

Data: mean ± SEM. *P < 0.05.

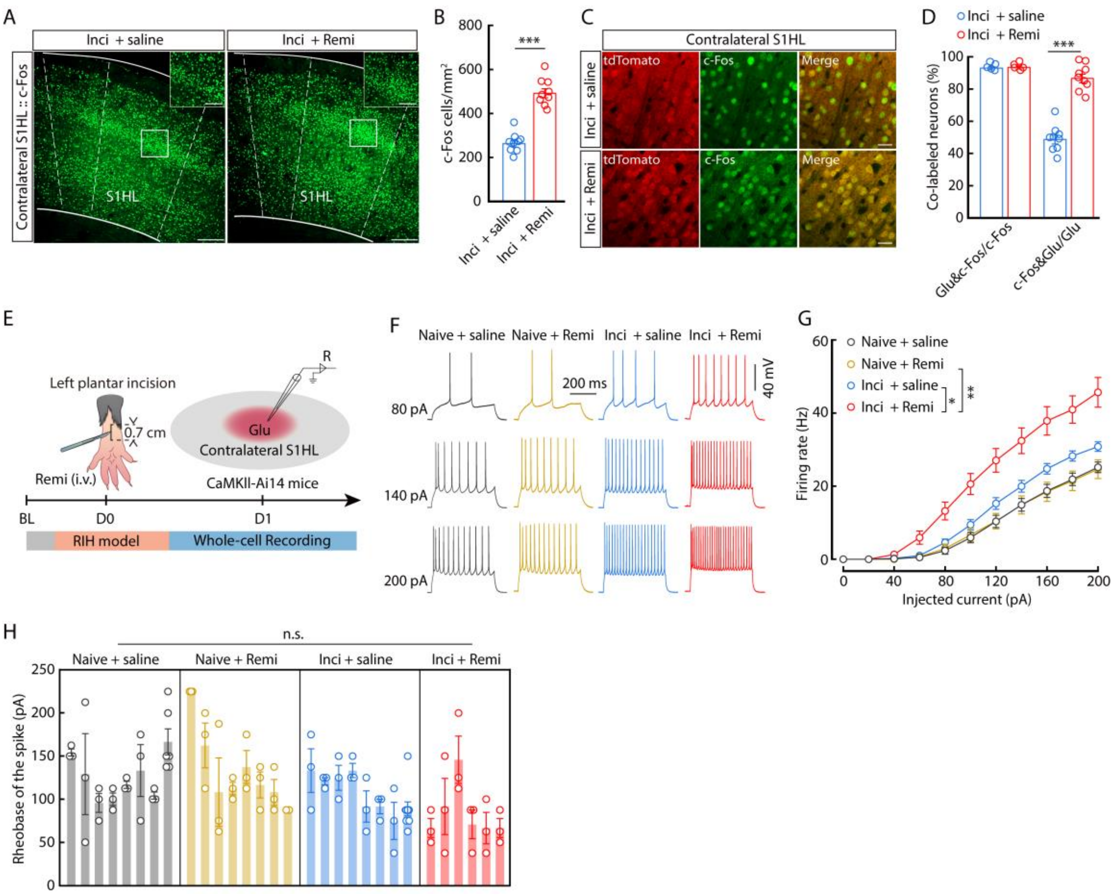

Supplemental Figure 18 | Enhanced activity of contralateral $S1HL^{Glu}$ neurons in RIH mice.

(A and B) Images (**A**) and quantitative data (**B**, n = 9 slices from 5 mice per group; $t_{(16)}$ = 8.899, P < 0.0001, unpaired Student's t-test) showing the expression of c-Fos in the contralateral S1HL in *CaMKII-Ai14* mice with plantar incision infused with Remi or saline. Scale bars, 200 μm and 100 μm (enlargement).

(C) Images showing co-localization of c-Fos-positive neurons (green) with tdTomato^{+} glutamatergic neurons (red). Scale bars, 20 μm.

(D) Summary data showing the percentage of c-Fos^{+} neurons expressing glutamate (left, n = 5 slices from 5 mice per group; $t_{(8)}$ = 0.1081, P = 0.9166, unpaired Student's t-test) and glutamate-labeled neurons expressing c-Fos (right, n = 9 slices from 5 mice per group; $t_{(16)}$ = 10.2, P < 0.0001, unpaired Student's t-test) in the contralateral S1HL in incisional mice treat with Remi or saline.

(E) Schematic of recording configuration in contralateral tdTomato^{+} $S1HL^{Glu}$ neurons of brain slices from *CaMKII-Ai14* mice.

(F and G) Representative traces (**F**) and quantitative data (**G**, n = 18-30 neurons from 6-8 mice per group; $F_{(3,976)}$ = 77.103, P < 0.0001; linear mixed models with post hoc Bonferroni's test) recorded from contralateral $S1HL^{Glu}$ neurons of depolarized current evoked action potentials.

(H) Statistics of the rheobase of action potentials recorded from contralateral $S1HL^{Glu}$ neurons

of mice from four groups (n = 18-30 neurons from 6-8 mice per group; $F_{(3,26)} = 3.127$, P = 0.0429, nested one-way ANOVA with post hoc Bonferroni's test).

Data: mean ± SEM. *$P < 0.05$, ***$P < 0.001$, n.s., not significant.

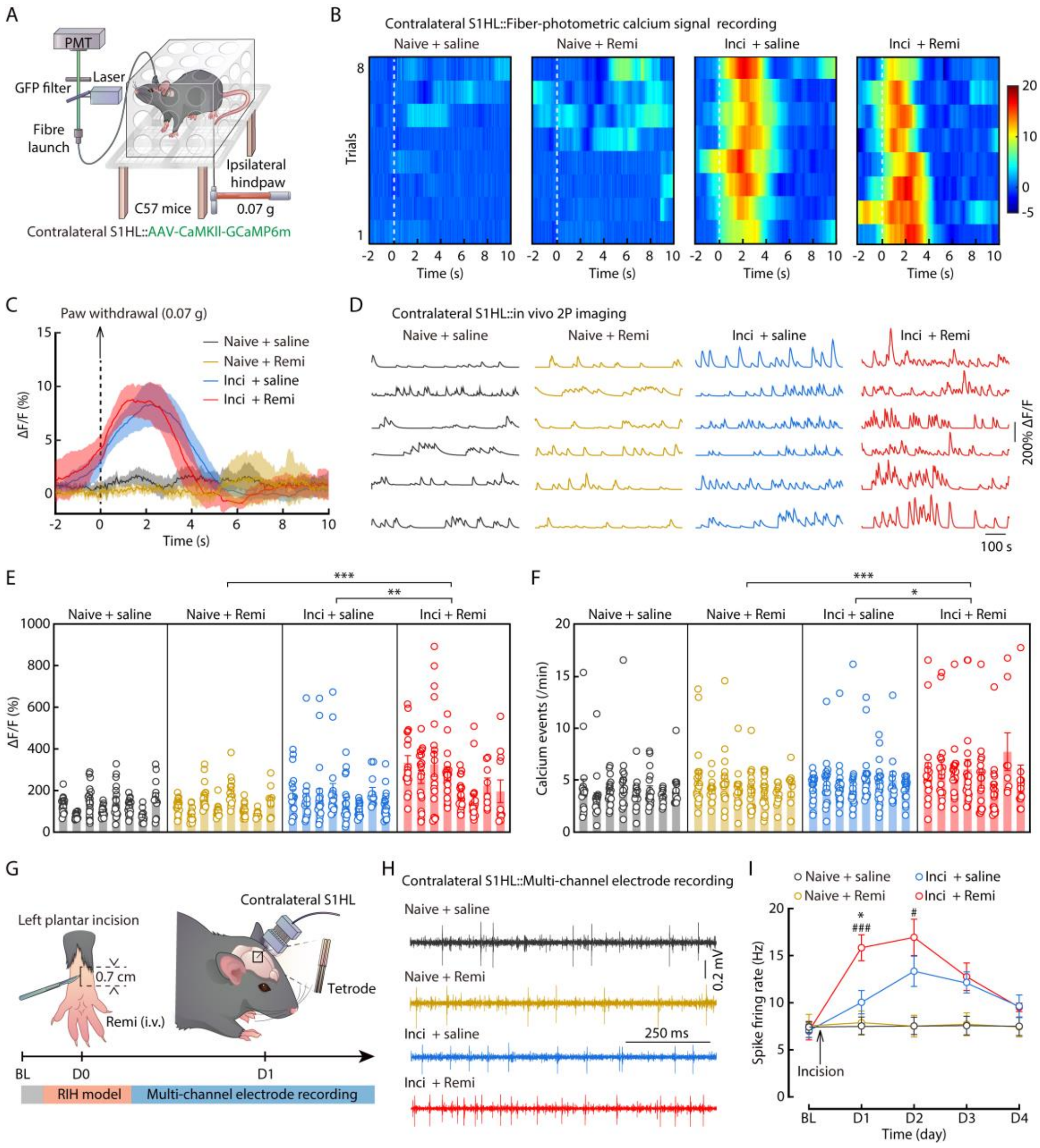

Supplemental Figure 19 | Enhanced activity in contralateral S1HL^{Glu} neurons detected by in vivo recordings in RIH mice.

(A) Schematic for fiber photometry experiments.

(B and C) The heatmaps (**B**) and the mean data (**C**) showing the change of Glu^{GCaMP6m} signals recorded from contralateral S1HL^{Glu} neurons in mice of four groups after subthreshold stimuli. Color scale at the right in (**B**) indicates ΔF/F (%).

(D) Spontaneous ΔF/F time series traces in GCaMP6f^{+} contralateral S1HL^{Glu} neurons of mice from four groups.

(E and F) Population average of spontaneous calcium responses (**E**, $F_{(3,28)}$ = 12.94, P < 0.0001) and quantifying difference in spontaneous calcium event rates (**F**, $F_{(3,549)}$ = 9.787, P < 0.0001) in GCaMP6^{+} contralateral S1HL^{Glu} neurons of mice from four groups (n = 133-139 neurons from 6-8 mice per group; nested one-way ANOVA with post hoc Bonferroni's test).

(G) Schematic illustration of the multi-channel electrophysiological recording in the contralateral S1HL of C57 mice. Enlargement showing the multichannel tetrode.

(H) Example traces of the spike firing recorded from contralateral $S1HL^{Glu}$ neurons in mice from four groups on postoperative day 1.

(I) Quantitative data of spike firing rate recorded from contralateral $S1HL^{Glu}$ neurons in mice of four groups (n = 6 mice per time point per group; $F_{(3,500.102)} = 10.572$, $P < 0.0001$; linear mixed models with post hoc Bonferroni's test).

Data: mean ± SEM. * indicating Inci + Remi *vs*. Inci + saline, # indicating Inci + Remi *vs*. Naive + Remi. **P* < 0.05, ***P* < 0.01, ****P* < 0.001; #*P* < 0.05, ###*P* <0.001; n.s., not significant.

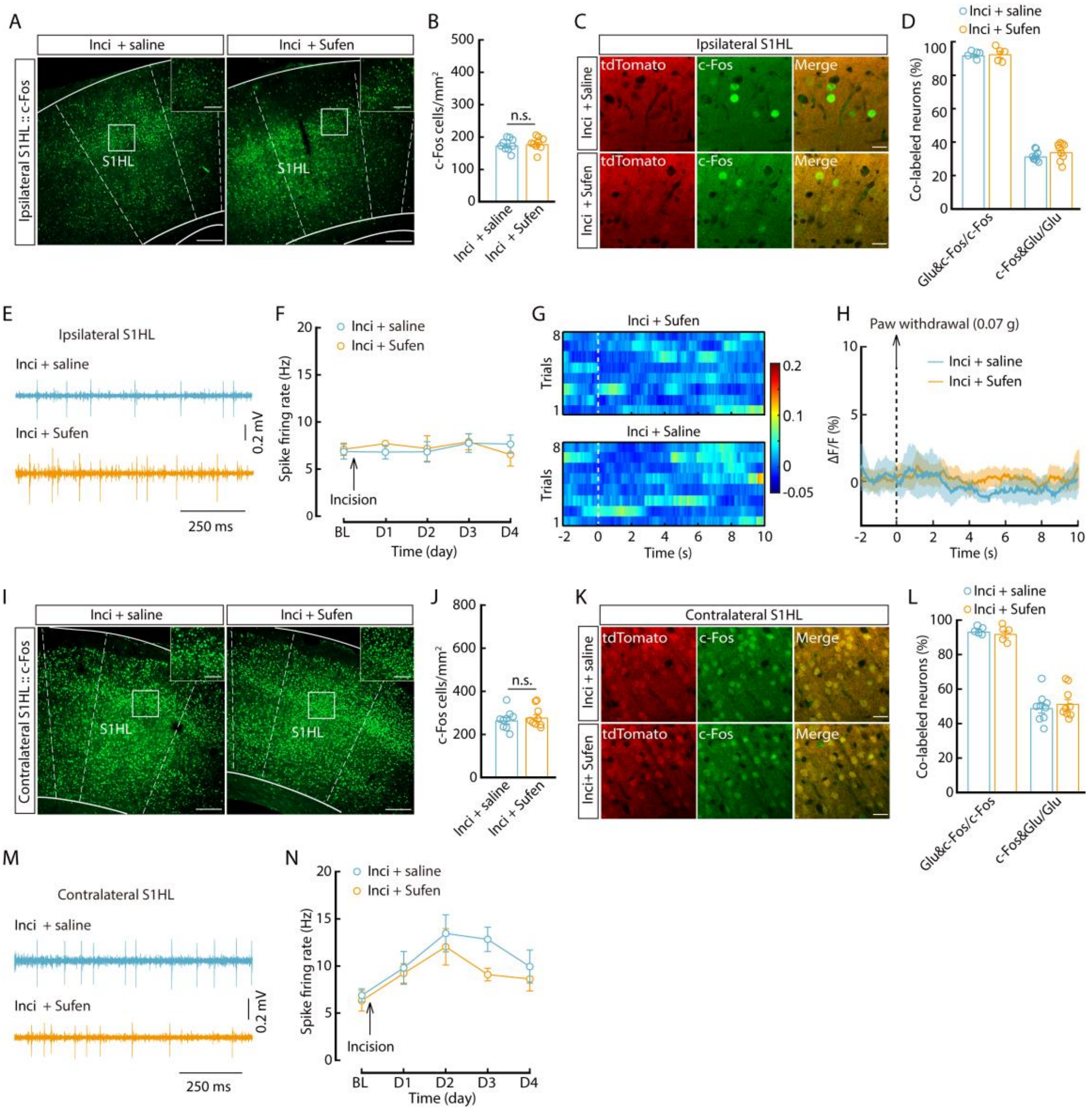

Supplemental Figure 20 | Bilateral S1HL^{Glu} neuronal activity does not change with sufentanil infusion in incisonal mice.

(A and B) Typical images (**A**) and quantitative data (**B**, n = 9 slices from 5 mice per group; $t_{(16)}$ = 0.4215, P = 0.679) showing the expression of c-Fos in the ipsilateral S1HL in Inci + saline and Inci + Sufen mice. Scale bars, 200 μm and 100 μm (enlargement).

(C) Images showing co-localization of c-Fos-positive neurons (green) with tdTomato^{+} glutamatergic neurons (red) in the ipsilateral S1HL. Scale bars, 20 μm.

(D) The percentage of c-Fos^{+} neurons expressing glutamate (left, n = 5 slices from 5 mice per group; $t_{(8)}$ = 0.1414, P = 0.8911) and glutamate-positive neurons expressing c-Fos (right, n = 9 slices from 5 mice per group; $t_{(16)}$ = 1.371, P = 0.1892) in the ipsilateral S1HL of Inci + saline and Inci + Sufen mice.

(E) Example traces of the spike firing recorded from ipsilateral S1HL^{Glu} neurons in Inci + saline and Inci + Sufen mice on postoperative day 1.

(F) Quantitative data of spike firing rate of ipsilateral S1HL^{Glu} neurons recorded from mice of two groups (*n* = 16-25 neurons from 6 mice per group; $F_{(1,208.999)} = 0.023$, $P = 0.88$).

(G and H) Heatmaps (**G**) and the mean data (**H**) showing Glu^{GCaMP6m} signals recorded from ipsilateral S1HL^{Glu} neurons in mice of two groups after subthreshold stimuli. Color scale at the right in (**G**) indicates ΔF/F (%).

(I and J) Typical images (**I**) and summary data (**J**, *n* = 9 slices from 5 mice per group; $t_{(16)} = 0.6305$, $P = 0.5373$) showing the expression of c-Fos in the ipsilateral S1HL of Inci + saline and Inci + Sufen mice. Scale bars, 200 μm and 100 μm (enlargement).

(**K**) Images showing co-localization of c-Fos-positive neurons (green) with tdTomato^{+} glutamatergic neurons (red) in the contralateral S1HL of mice with plantar incision infused with Sufen or saline. Scale bars, 20 μm.

(L) Percentage of c-Fos^{+} neurons expressing glutamate (left, *n* = 5 slices from 5 mice per group; $t_{(8)} = 0.6145$, $P = 0.556$) and glutamate^{+} neurons expressing c-Fos (right, *n* = 9 slices from 5 mice per group; $t_{(16)} = 0.6024$, $P = 0.5554$) in the contralateral S1HL of mice with plantar incision infused with Sufen or saline.

(M) Example traces of the spike firing recorded from contralateral S1HL^{Glu} neurons in Inci + saline and Inci + Sufen mice.

(N) Summary data of spike firing rate of contralateral S1HL^{Glu} neurons recorded from mice of two groups (*n* = 15-23 neurons from 6 mice per time point per group; $F_{(1, 300.01)} = 0.036$, P = 0.794)**.**

Data: mean ± SEM. Unpaired Student's *t*-test in (**B-D**), (**J**) and (**L**); linear mixed models with post hoc Bonferroni's test in (**F**) and (**N**).

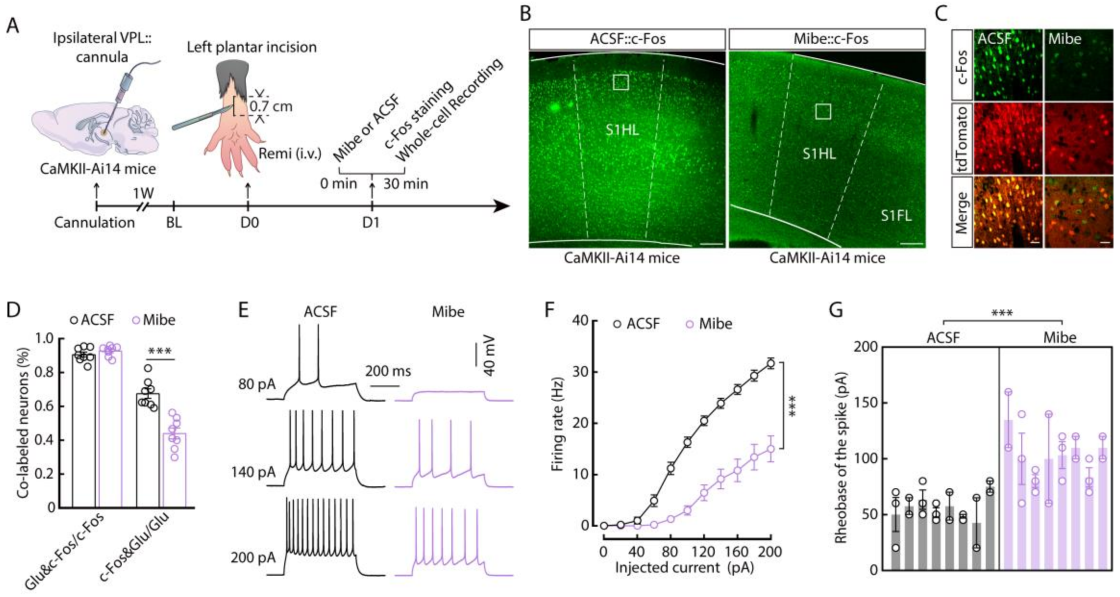

Supplemental Figure 21 | Postoperative antagonization of T-type calcium channels reduces the activity of S1HL^{Glu} neurons.

(A) Schematic of the experimental procedure for RIH model mice injected with mibefradil (Mibe) or ACSF.

(B) Immunohistochemistry staining to detect c-Fos-positive neurons in the ipsilateral S1HL of *CaMKII-Ai14* RIH mice treated with ACSF (left) or Mibe (right). Scale bars, 200 μm.

(C) Images showing co-localization of c-Fos-positive neurons (green) with tdTtomato$^+$ neurons (red). Scale bars, 20 μm.

(D) Summary data of the percentage of c-Fos$^+$ neurons expressing glutamate (left, $t_{(14)} = 1.182$, $P = 0.257$) and glutamate-positive neurons expressing c-Fos (right, $t_{(14)} = 5.448$, $P < 0.0001$) in the ipsilateral S1HL of RIH mice treated with ACSF or Mibe (n = 8 slices from 5 mice per group; unpaired Student's *t*-test) .

(E and F) Representative traces (**E**) and quantitative data (**F**, n = 20 neurons from 8 mice per group; $F_{(1,468.97)} = 19.741$, $P < 0.0001$, linear mixed models with post hoc Bonferroni's test) of depolarizing current evoked action potentials recorded in ipsilateral S1HL^{Glu} neurons of RIH mice treated with ACSF or Mibe.

(G) Statistical analysis of the rheobase of action potentials recorded in ipsilateral S1HL^{Glu} neurons of RIH mice post-operatively treated with ACSF or Mibe (n = 20 neurons from 8 mice per group; $t_{(38)} = 6.306$, $P < 0.0001$, nested *t*-test).

Data: mean ± SEM. ***$P < 0.001$. n.s., not significant.

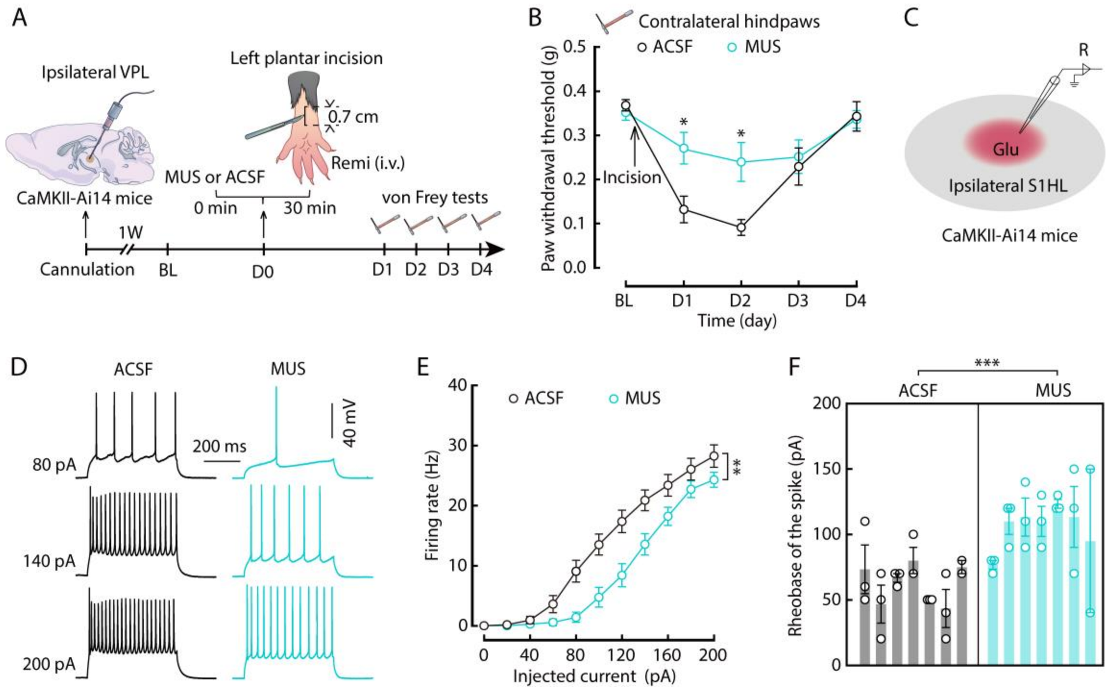

Supplemental Figure 22 | Preoperative silencing of neuronal activity in the VPL reverses pain sensitization and hyperactivity in S1HL^{Glu} neruons of RIH mice.

(A) Schematic diagram of the experimental procedure for injection with MUS or ACSF.

(B) Quantitative data showing the significant relief on postoperative allodynia in RIH mice by preoperative local ipsilateral infusion of MUS into the VPL (n = 10 mice per group; $F_{(1,18)}$ = 6.273, P = 0.0221; two-way RM ANOVA with post hoc Bonferroni's test).

(C) Schematic of whole-cell recording configuration in ipsilateral tdTomato$^+$ S1HL^{Glu} neurons in brain slices from *CaMKII-Ai14* RIH mice.

(D and E) Representative traces (**D**) and summary data (**E**, n = 20 neurons from 7 mice per group; $F_{(1,399.858)}$ = 10.338, P = 0.001; linear mixed models with post hoc Bonferroni's test**)** of depolarized current evoked action potentials recorded from ipsilateral S1HL^{Glu} neurons of RIH mice preoperatively treated with MUS or ACSF on postoperative day 1.

(F) Quantitation of the rheobase of action potentials recorded from ipsilateral S1HL^{Glu} neurons of RIH mice preoperatively treated with MUS or ACSF (n = 20 neurons from 7 mice per group; $t_{(12)}$ = 5.417, P = 0.0002; nested t-test).

Data: mean ± SEM. *P < 0.05, **P < 0.01, ***P < 0.001. n.s., not significant.

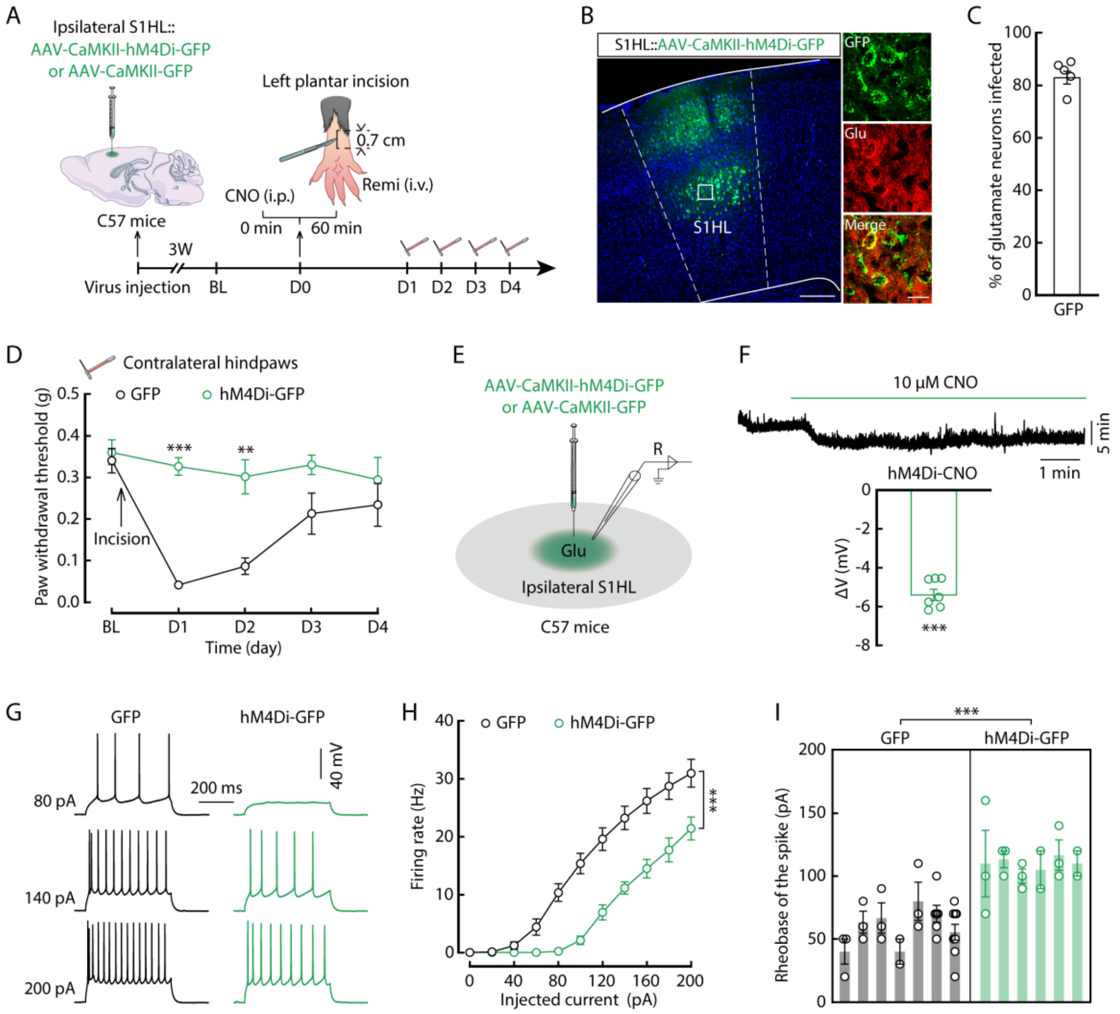

Supplemental Figure 23 | Pre-operative chemogenetic inhibition of S1HL^{Glu} neurons reduces pain sensitization and activity in S1HL^{Glu} neurons of RIH mice.

(A) Schematic diagram of the experimental procedure for virus injection into the S1HL and preoperative intraperitoneal injection of CNO and behavioral tests in C57 mice.

(B) Representative images exhibiting the injection site of AAV-CaMKII-hM4Di-GFP within the S1HL (left) and GFP^{+} positive neurons (green) co-localize with glutamate immunofluorescence (red, right). Scale bars, 200 μm (left) and 20 μm (right).

(C) Summarized data showing the percentage of GFP^{+} positive neurons co-expressed with glutamate immunofluorescence (n = 5 slices from 5 mice).

(D) Quantitative data showing the significant relief on postoperative allodynia in RIH mice by preoperative chemogenetic inhibition of ipsilateral S1HL^{Glu} neurons (n = 8 mice per time point per group; $F_{(1,14)} = 50.55$, $P < 0.0001$; two-way RM ANOVA with post hoc Bonferroni's test).

(E) Schematic of recording configuration in ipsilateral S1HL^{Glu} neurons.

(F) Whole-cell recording showing the effect of CNO on AAV-DIO-hM4Di-mCherry expressing S1HL^{Glu} neurons (n = 7 neurons from 6 mice; $t_{(5)} = 18.74$, $P < 0.0001$; one sample t-test).

(G and H) Representative traces **(G)** and summary data (**H**, n = 29 neurons from 7 GFP mice;

n = 16 neurons from 6 hM4Di-GFP mice; $F_{(1,448)} = 75.877$, $P < 0.0001$; linear mixed models with post hoc Bonferroni's test) of depolarizing current evoked action potentials recorded from ipsilateral hM4Di- or GFP-expressing $S1HL^{Glu}$ neurons of RIH mice preoperatively injected with CNO (i.p.) on postoperative day 1.

(I) Statistics of the rheobase recorded from ipsilateral hM4Di- or GFP-expressing $S1HL^{Glu}$ neurons of RIH mice preoperatively injected with CNO (i.p.) on postoperative day 1 (n = 29 neurons from 7 GFP mice; n = 16 neurons from 6 hM4Di-GFP mice; $t_{(11)} = 7.556$, $P < 0.0001$, nested t-test).

Data: mean ± SEM. **$P < 0.01$, ***$P < 0.001$.

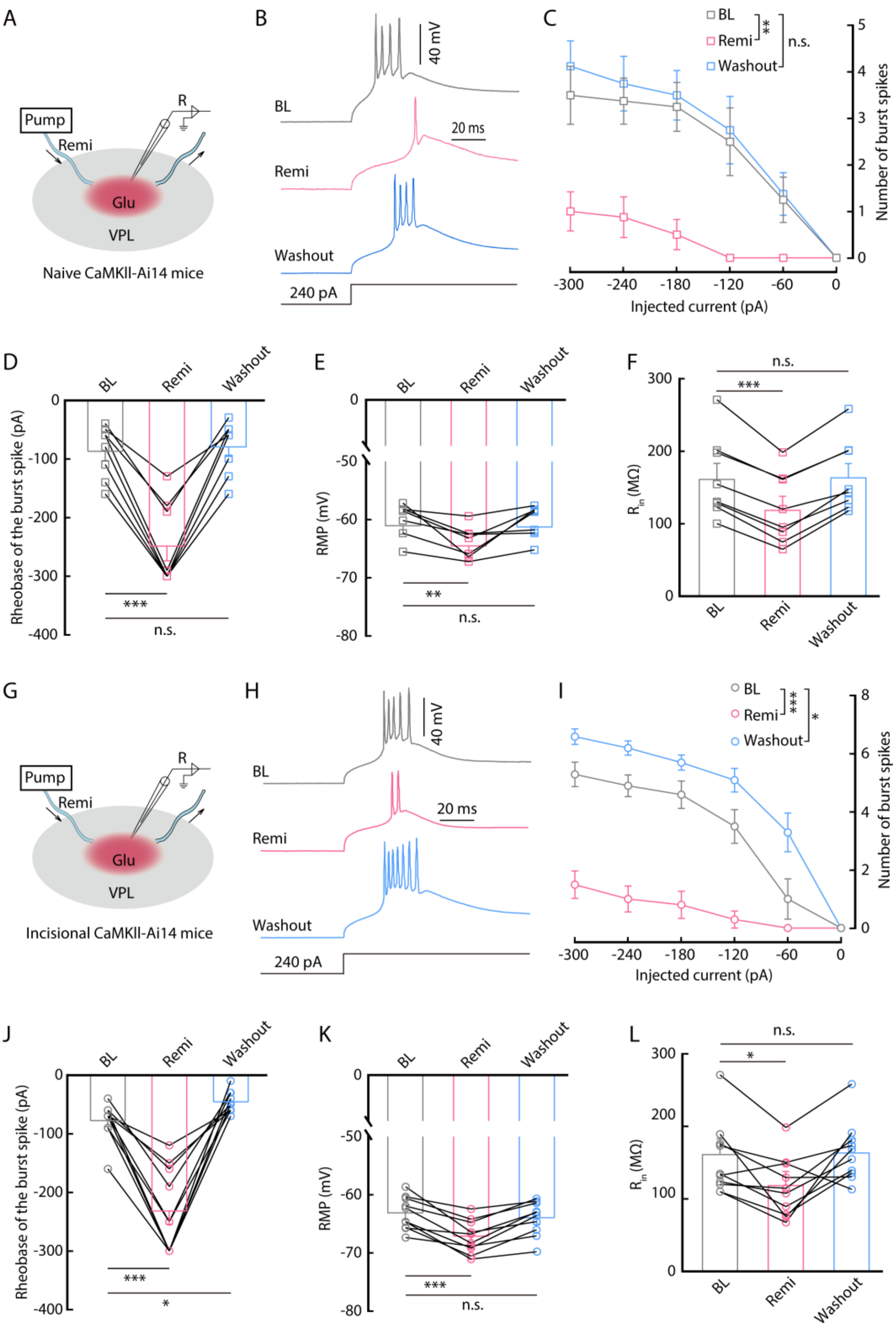

Supplemental Figure 24 | Effects of remifentanil perfusion on VPL^{Glu} neuronal activity in brain slices.

(**A**) Schematic of electrophysiological recording procedure.

(**B** and **C**) Representative traces (**B**) and quantification (**C**) of hyperpolarized current-induced burst firing recorded in VPL^{Glu} neurons before (Baseline, BL), during, and after (washout) perfusion of remifentanil (n = 8 neurons from 8 mice; $F_{(2,21)}$ = 9.162, P = 0.0014).

(**D-F**) Quantification of the rheobase of the burst firing (D, $F_{(2,14)}$ = 73.19, P < 0.0001), RMP (E, $F_{(2,14)}$ = 9.425, P = 0.0026), and Rin (F, $F_{(2,14)}$ = 66.93, P < 0.0001) recorded in VPL^{Glu} neurons (n = 8 neurons from 8 mice).

(**G**) Schematic of electrophysiological recording procedure.

(**H** and **I**) Representative traces (**H**) and quantitative data (**I**) of hyperpolarizing current-induced burst firing recorded in VPL^{Glu} neurons before (Baseline, BL), during and after (washout) perfusion of remifentanil (n = 10 neurons from 10 mice; $F_{(2,27)}$ = 42.01, P < 0.0001).

(**J-L**) Quantification of the rheobase of the burst firing (**J**, $F_{(2,18)}$ = 53.87, P < 0.0001), RMP (**K**, $F_{(2,18)}$ = 18.17, P < 0.0001) and R_{in} (**L**, $F_{(2,18)}$ = 8.016, P = 0.0032) recorded in VPL^{Glu} neurons (n = 10 neurons from 10 mice).

Data: mean ± SEM. *P < 0.05, **P < 0.01, ***P < 0.001. n.s., not significant. Tow-way RM ANOVA with post hoc Bonferroni's test in (**C**) and (**I**); one-way RM ANOVA with post hoc Bonferroni's test in (**D-F**) and (**J-L**).

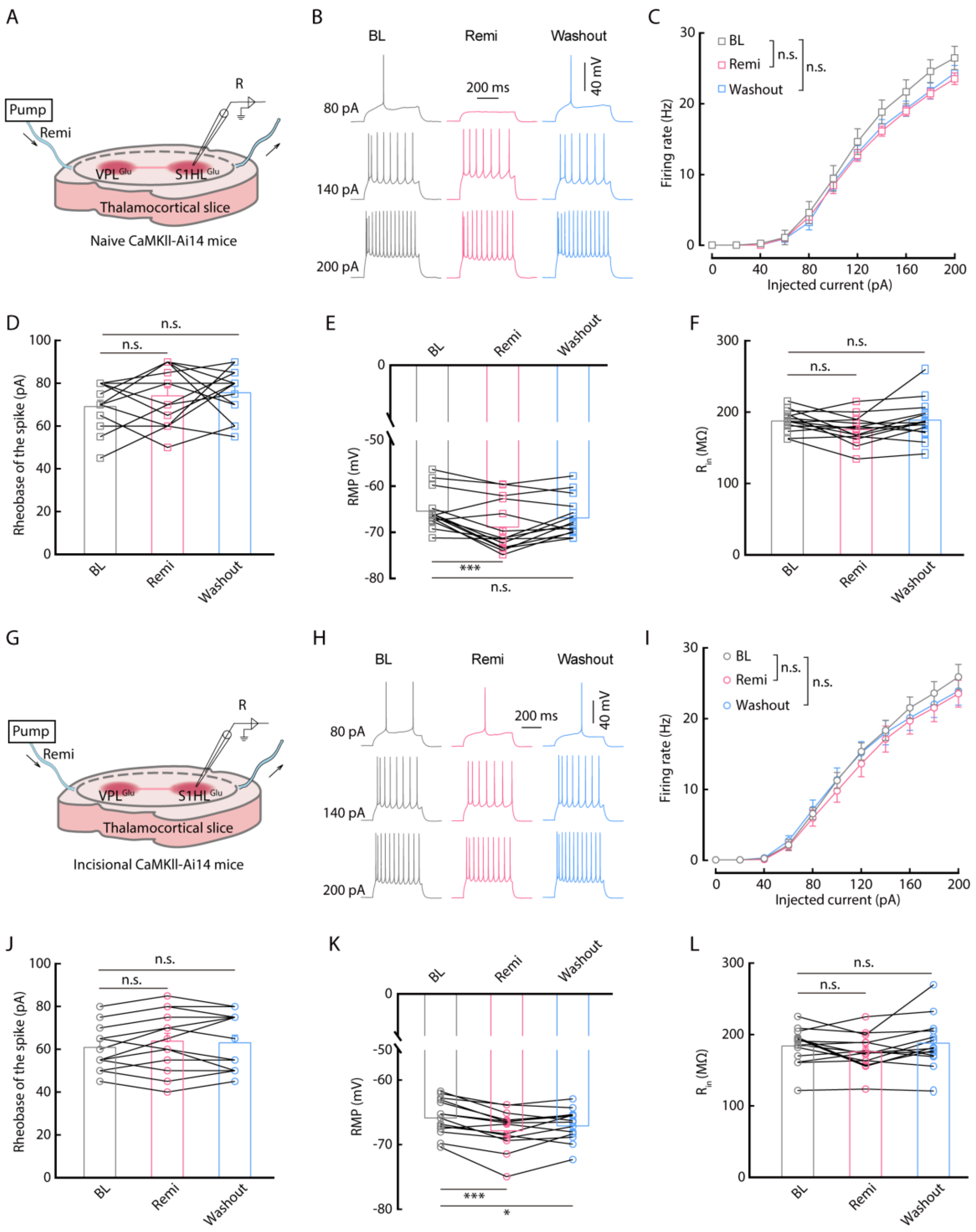

Supplemental Figure 25 | Effects of remifentanil perfusion on the activity of S1HL^{Glu} neurons in thalamocortical brain slices from naïve and incisional mice.

(**A**) Schematic of electrophysiological recordings in S1HL^{Glu} neurons.

(**B** and **C**) Representative traces (**B**) and quantitative data (**C**) of depolarizing current-induced action potentials recorded in S1HL^{Glu} neurons before (Baseline), during, and after (washout) perfusion of remifentanil (*n* = 14 neurons from 14 mice; $F_{(2,39)}$ = 0.379, *P* = 0.3924).

(**D-F**) Quantification of the rheobase of the firing (**D**, $F_{(2,26)}$ = 1.754, *P* = 0.1929), RMP (**E**, $F_{(2,26)}$ = 8.795, *P* = 0.0012) and Rin (**F**, $F_{(2,26)}$ = 3.74, *P* = 0.0374) recorded in S1HL^{Glu} neurons (*n* = 14 neurons from 14 mice).

(**G**) Schematic for electrophysiological recordings in $S1HL^{Glu}$ neurons upon remifentanil perfusion in brain slices from incisional *CaMKII-Ai14* mice.

(**H** and **I**) Representative traces (**H**) and quantitative data (**I**) of depolarizing current-induced action potentials recorded in $S1HL^{Glu}$ neurons before (Baseline), during and after (washout) perfusion of remifentanil (n = 14 neurons from 14 mice; $F_{(2,39)} = 0.2848$, $P = 0.7537$).

(**J-L**) Quantification of the firing rheobase (**J**, $F_{(2,26)} = 1.649$, $P = 0.2118$), RMP (**K**, $F_{(2,26)} = 11.62$, $P = 0.0002$) and R_{in} (**L**, $F_{(2,26)} = 2.832$, $P = 0.0771$) recorded in $S1HL^{Glu}$ neurons (n = 14 neurons from 14 mice).

Data: mean ± SEM. *$P < 0.05$, ***$P < 0.001$. n.s., not significant. Tow-way RM ANOVA with post hoc Bonferroni's test in (**C**) and (**I**); one-way RM ANOVA with post hoc Bonferroni's test in (**D-F**) and (**J-L**).

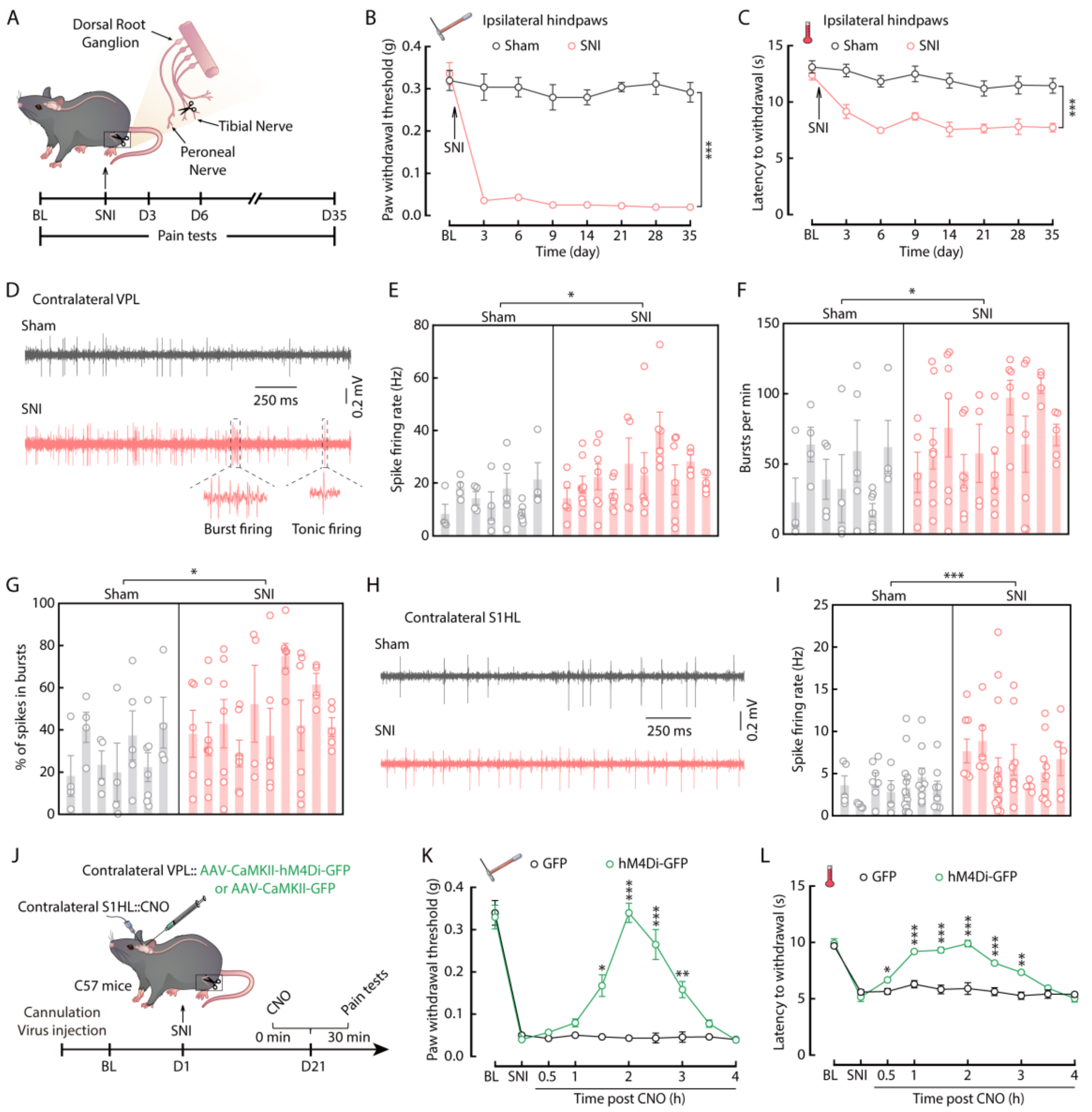

Supplemental Figure 26 | The VPL^{Glu}→S1HL^{Glu} pathway regulates chronic pain in SNI mice.

(**A**) Schematic of the experimental procedure for SNI model induction and behavioral tests.

(**B**) Time course of changes in the response threshold to mechanical force assessed using a von Frey test (n = 10 mice per group; $F_{(1,18)} = 1050$, $P < 0.0001$).

(**C**) Time course of changes in the response to thermal pain assessed using a Hargreaves test (n = 10 mice per group; $F_{(1,18)} = 169.3$, $P < 0.0001$).

(**D**) Example traces of spike firing recorded in contralateral VPL^{Glu} neurons of sham and SNI mice. Tonic and burst firing are highlighted by dashed frames.

(**E-G**) Quantitative data of total spike firing rate (**E**, $t_{(15)} = 2.615$, $P = 0.0195$), burst number/min (**F**, $t_{(15)} = 2.393$, $P = 0.0302$), and percentage of spikes in bursts (**G**, $t_{(15)} = 5.77$, $P = 0.0297$) recorded in contralateral VPL^{Glu} neurons of sham and SNI mice (n = 32 neurons from 7 Sham mice; n = 59 neurons from 10 SNI mice).

(**H** and **I**) Example traces (**H**) and quantitative data (**I**) of spike firing recorded in contralateral $S1HL^{Glu}$ neurons of sham and SNI mice (n = 52 neurons from 7 Sham mice; n = 56 neurons from 7 SNI mice, $t_{(106)} = 3.51$, $P = 0.0007$).

(**J**) Schematic of the experimental procedure for contralateral VPL injection with AAV-CaMKII-hM4Di-GFP or AAV-CaMKII-GFP and contralateral S1HL injection with CNO in SNI mice.

(**K** and **L**) Quantitative data of mechanical (**K**, $F_{(1,14)} = 100.3$, $P < 0.0001$) and thermal (**L**, $F_{(1,14)} = 209.8$, $P < 0.0001$) pain in SNI mice with chemogenetic inhibition of the contralateral VPL^{Glu}→$S1HL^{Glu}$ pathway (n = 8 mice per time point per group).

Data: mean ± SEM. *$P < 0.05$, **$P < 0.01$, ***$P < 0.001$. Two-way RM ANOVA with post hoc Bonferroni's test in (**B**), (**C**), (**K**) and (**L**); nested *t*-test in (**E-G**) and (**I**).

Supplemental Video 1.

Optical-fiber-based calcium signals recording of ipsilateral VPL^{Glu} neurons evoked by 0.07 g von Frey filament stimuli on the contralateral hindpaws of RIH mice.

Supplemental Video 2.

Optical-fiber-based calcium signals recording of ipsilateral $S1HL^{Glu}$ neurons evoked by 0.07 g von Frey filament stimuli on the contralateral hindpaws of RIH mice.

Supplemental Table 1. Statistical analyses related to Figure 1-7 and Supplemental Figure 1-26.

Figure	Conditions (sample size)		Analysis	*P* value	*t* or *F* value
Figure 1B	Inci + saline (11 mice)	Inci + Remi (12 mice)	Two-way RM ANOVA with post hoc Bonferroni's test	$P = 0.0006$	$F_{(1,21)} = 16.14$
	BL			$P > 0.9999$	
	D1			$P = 0.5090$	
	D2			$P = 0.0738$	
	D3			$P = 0.0146$	
	D4			$P > 0.9999$	
Figure 1C	Inci + saline (11 mice)	Inci + Remi (12 mice)	Two-way RM ANOVA with post hoc Bonferroni's test	$P < 0.0001$	$F_{(1,21)} = 52.68$
	BL			$P > 0.9999$	
	D1			$P = 0.0123$	
	D2			$P = 0.0001$	
	D3			$P = 0.0053$	
	D4			$P > 0.9999$	
Figure 1D	Inci + saline (10 mice)	Inci + Remi (9 mice)	Two-way RM ANOVA with post hoc Bonferroni's test	$P = 0.3353$	$F_{(1,17)} = 0.9832$
	BL			$P > 0.9999$	
	D1			$P > 0.9999$	
	D2			$P > 0.9999$	
	D3			$P > 0.9999$	
	D4			$P > 0.9999$	
Figure 1E	Inci + saline (10 mice)	Inci + Remi (9 mice)	Two-way RM ANOVA with post hoc Bonferroni's test	$P = 0.0339$	$F_{(1,17)} = 5.323$
	BL			$P > 0.9999$	
	D1			$P =0.1652$	
	D2			$P =0.5174$	
	D3			$P > 0.9999$	
	D4			$P > 0.9999$	
Figure 1F	Inci + saline (10 mice)	Inci + Remi (10 mice)	Two-way RM ANOVA with post hoc Bonferroni's test	$P = 0.0856$	$F_{(1,18)} = 3.308$
	BL			$P > 0.9999$	
	D1			$P > 0.9999$	
	D2			$P =0.5302$	
	D3			$P > 0.9999$	
	D4			$P > 0.9999$	
Figure 1G	Inci + saline (10 mice)	Inci + Remi (10 mice)	Two-way RM ANOVA with post hoc Bonferroni's test	$P = 0.0234$	$F_{(1,18)} = 6.135$
	BL			$P > 0.9999$	
	D1			$P =0.1652$	
	D2			$P =0.5174$	
	D3			$P > 0.9999$	
	D4			$P > 0.9999$	
Figure 1K			One-way ANOVA with post hoc Bonferroni's test	$P = 0.0047$	$F_{(3,36)} = 5.113$
	Naive + saline (10 mice)	Naive+Remi (10 mice)		$P > 0.9999$	

	Naive + saline (10 mice)	Inci + saline (10 mice)		$P > 0.9999$	
	Naive + saline (10 mice)	Inci + Remi (10 mice)		$P = 0.0139$	
	Inci + saline (10 mice)	Inci + Remi (10 mice)		$P = 0.0083$	
Figure 2L (left)			Linear mixed models with post hoc Bonferroni's test	$P = 0.002$	$F_{(3,445.018)} = 5.063$
	Naive + Saline (8 mice)	Naive + Remi (8 mice)		$P > 0.999$	
	Naive + Saline (8 mice)	Inci + saline (8 mice)		$P > 0.999$	
	Naive + Remi (8 mice)	Inci + Remi (8 mice)		$P < 0.0001$	
	Inci + saline (8 mice)	Inci + Remi (8 mice)		$P < 0.0001$	
	BL		Nested one-way ANOVA with post hoc Bonferroni's test	$P = 0.9648$	$F_{(3,28)} = 0.09032$
	D1		Nested one-way ANOVA with post hoc Bonferroni's test	$P < 0.0001$	$F_{(3,28)} = 12.72$
	Naïve + saline (43 neurons)	Naïve + Remi (47 neurons)		$P > 0.9999$	
	Inci + saline (91 neurons)	Inci + Remi (83 neurons)		$P < 0.0001$	
	D2		Nested one-way ANOVA with post hoc Bonferroni's test	$P = 0.0011$	$F_{(3,28)} = 7.124$
	Naïve + saline (38 neurons)	Naïve + Remi (40 neurons)		$P > 0.9999$	
	Inci + saline (65 neurons)	Inci + Remi (67 neurons)		$P = 0.0038$	
	D3		Nested one-way ANOVA with post hoc Bonferroni's test	$P = 0.0013$	$F_{(3,28)} = 6.875$
	Naïve + saline (38 neurons)	Naïve + Remi (37 neurons from 8 mice)		$P > 0.9999$	
	Inci + saline (58 neurons)	Inci + Remi (60 neurons from 8 mice)		$P = 0.0037$	
	D4		Nested one-way ANOVA with post hoc Bonferroni's test	$P = 0.5566$	$F_{(3,28)} = 7.7058$
Figure 2L (middle)			Linear mixed models with post hoc Bonferroni's test	$P < 0.0001$	$F_{(3,459.614)} = 6.161$
	Naive + Saline (37-43 neurons from 8 mice)	Naive + Remi (37-47 neurons from 8 mice)		$P > 0.999$	
	Naive + Saline (37-43 neurons from 8 mice)	Inci + saline (52-91 neurons from 8 mice)		$P > 0.999$	
	Naive + Remi	Inci + Remi		$P = 0.006$	

	(37-47 neurons from 8 mice)	(60-83 neurons from 8 mice)			
	Inci + saline (52-91 neurons from 8 mice)	Inci + Remi (60-83 neurons from 8 mice)		$P < 0.0001$	
	BL		Nested one-way ANOVA with post hoc Bonferroni's test	$P = 0.5311$	$F_{(3,28)} = 0.7508$
	D1		Nested one-way ANOVA with post hoc Bonferroni's test	$P = 0.0011$	$F_{(3,28)} = 7.063$
	Naïve + saline (43 neurons)	Naïve + Remi (47 neurons)		$P > 0.9999$	
	Inci + saline (91 neurons)	Inci + Remi (83 neurons)		$P < 0.0001$	
	D2		Nested one-way ANOVA with post hoc Bonferroni's test	$P = 0.0011$	$F_{(3,28)} = 7.083$
	Naïve + saline (38 neurons)	Naïve + Remi (40 neurons)		$P > 0.9999$	
	Inci + saline (65 neurons)	Inci + Remi (67 neurons)		$P = 0.0051$	
	D3		Nested one-way ANOVA with post hoc Bonferroni's test	$P = 0.0127$	$F_{(3,28)} = 4.315$
	Naïve + saline (38 neurons)	Naïve + Remi (37 neurons)		$P > 0.9999$	
	Inci + saline (58 neurons)	Inci + Remi (60 neurons)		$P = 0.0489$	
	D4		Nested one-way ANOVA with post hoc Bonferroni's test	$P = 0.1683$	$F_{(3,28)} = 0.1683$
Figure 2L (right)			Linear mixed models with post hoc Bonferroni's test	$P = 0.005$	$F_{(3,418.152)} = 15.572$
	Naive + Saline (37-43 neurons from 8 mice)	Naive + Remi (37-47 neurons from 8 mice)		$P > 0.999$	
	Naive + Saline (37-43 neurons from 8 mice)	Inci + saline (52-91 neurons from 8 mice)		$P > 0.999$	
	Naive + Remi (37-47 neurons from 8 mice)	Inci + Remi (60-83 neurons from 8 mice)		$P = 0.006$	
	Inci + saline (52-91 neurons from 8 mice)	Inci + Remi (60-83 neurons from 8 mice)		$P = 0.04$	
	BL		Nested one-way ANOVA with post hoc Bonferroni's test	$P = 0.561$	$F_{(3,28)} = 0.6983$
	D1		Nested one-way ANOVA with post hoc Bonferroni's test	$P = 0.0006$	$F_{(3,28)} = 7.775$

	Naïve + saline (43 neurons)	Naïve + Remi (47 neurons from 8 mice)		$P > 0.9999$	
	Inci + saline (91 neurons)	Inci + Remi (83 neurons)		$P < 0.0001$	
	D2		Nested one-way ANOVA with post hoc Bonferroni's test	$P = 0.0675$	$F_{(3,28)} = 2.661$
	Naïve + saline (38 neurons)	Naïve + Remi (40 neurons)		$P > 0.9999$	
	Inci + saline (65 neurons)	Inci + Remi (67 neurons)		$P = 0.0127$	
	D3		Nested one-way ANOVA with post hoc Bonferroni's test	$P = 0.0493$	$F_{(3,28)} = 2.96$
	Naïve + saline (38 neurons)	Naïve + Remi (37 neurons from 8 mice)		$P > 0.9999$	
	Inci + saline (58 neurons)	Inci + Remi (60 neurons)		$P = 0.0489$	
	D4		Nested one-way ANOVA with post hoc Bonferroni's test	$P = 0.7057$	$F_{(3,28)} = 0.4698$
Figure 2M			Linear mixed models with post hoc Bonferroni's test	$P = 0.354$	$F_{(3,527.508)} = 1.086$
	Naive + Saline (37-43 neurons from 8 mice)	Naive + Remi (37-47 neurons from 8 mice)		$P > 0.999$	
	Naive + Saline (37-43 neurons from 8 mice)	Inci + saline (52-91 neurons from 8 mice)		$P > 0.999$	
	Naive + Remi (37-47 neurons from 8 mice)	Inci + Remi (60-83 neurons from 8 mice)		$P = 0.253$	
	Inci + saline (52-91 neurons from 8 mice)	Inci + Remi (60-83 neurons from 8 mice)		$P = 0.224$	
Figure 3C			Chi-square test	$P = 0.0011$	
	Naive + Saline (60 neurons from 6 mice)	Naive + Remi (65 neurons from 6 mice)		$P = 0.5337$	
	Naive + Saline (60 neurons from 6 mice)	Inci + saline (64 neurons from 6 mice)		$P = 0.2439$	
	Naive + Remi (65 neurons from 6 mice)	Inci + Remi (61 neurons from 6 mice)		$P = 0.0047$	
	Inci + saline (64 neurons from 6 mice)	Inci + Remi (61 neurons from 6 mice)		$P = 0.022$	
Figure 3E			Linear mixed models with post hoc Bonferroni's test	$P < 0.0001$	$F_{(3,529.867)} = 7.332$
	Naive + Saline	Naive + Remi		$P = 0.769$	

	(25 neurons from 10 mice)	(25 neurons from 10 mice)			
	Naive + Saline (25 neurons from 10 mice)	Inci + saline (28 neurons from 10 mice)		$P > 0.999$	
	Naive + Remi (25 neurons from 10 mice)	Inci + Remi (30 neurons from 10 mice)		$P = 0.001$	
	Inci + saline (28 neurons from 10 mice)	Inci + Remi (30 neurons from 10 mice)		$P = 0.024$	
Figure 3F			Nested one-way ANOVA with post hoc Bonferroni's test	$P = 0.005$	$F_{(3,104)} = 4.722$
	Naive + Saline (25 neurons from 10 mice)	Naive + Remi (25 neurons from 10 mice)		$P > 0.9999$	
	Naive + Saline (25 neurons from 10 mice)	Inci + saline (28 neurons from 10 mice)		$P > 0.9999$	
	Naive + Remi (25 neurons from 10 mice)	Inci + Remi (30 neurons from 10 mice)		$P = 0.0371$	
	Inci + saline (28 neurons from 10 mice)	Inci + Remi (30 neurons from 10 mice)		$P = 0.0103$	
Figure 3P（left）	EYFP (50 neurons from 8 mice)	eNpHR3.0 (50 neurons from 8 mice)	Linear mixed models with post hoc Bonferroni's test	$P = 0.048$	$F_{(1, 280.105)} = 14.96$
	Light on		Nested *t*-test analysis	$P = 0.0136$	$F_{(1, 14)} = 9.299$
Figure 3P (right)	EYFP (50 neurons from 8 mice)	eNpHR3.0 (50 neurons from 8 mice)	Linear mixed models with post hoc Bonferroni's test	$P = 0.022$	$F_{(1,286.984)} = 5.312$
	Light on		Nested *t*-test analysis	$P = 0.0382$	$F_{(1,14)} = 12.058$
Figure 3Q	EYFP (8 mice)	eNpHR3.0 (8 mice)	Two-way RM ANOVA with post hoc Bonferroni's test	$P = 0.0005$	$F_{(2,32)} = 9.602$
	BL			$P > 0.9999$	
	Light on			$P = 0.0002$	
	Light off			$P > 0.9999$	
Figure 4B			One-way ANOVA with post hoc Bonferroni's test	$P = 0.0011$	$F_{(3,22)} = 7.633$
	Naive + Saline (6 mice)	Naive + Remi (6 mice)		$P > 0.9999$	
	Naive + Saline (6 mice)	Inci + saline (7 mice)		$P = 0.0493$	
	Naive + Remi (6 mice)	Inci + Remi (7 mice)		$P = 0.0063$	
	Inci + saline (7 mice)	Inci + Remi (7 mice)		$P > 0.9999$	
Figure 4D			Linear mixed models with post hoc Bonferroni's test	$P < 0.0001$	$F_{(3,569.088)} = 46.526$
	Naive + Saline (14 neurons from 6 mice)	Naive + Remi (14 neurons from 6 mice)		$P > 0.999$	
	Naive + Saline (14 neurons	Inci + saline (14 neurons		$P > 0.999$	

	from 6 mice)	from 6 mice)			
	Naive + Remi (14 neurons from 6 mice)	Inci + Remi (14 neurons from 6 mice)		$P = 0.01$	
	Inci + saline (14 neurons from 6 mice)	Inci + Remi (14 neurons from 6 mice)		$P = 0.03$	
Figure 4E			Nested one-way ANOVA with post hoc Bonferroni's test	$P = 0.0007$	$F_{(3,52)} = 6.694$
	Naive + Saline (14 neurons from 6 mice)	Naive + Remi (14 neurons from 6 mice)		$P > 0.9999$	
	Naive + Saline (14 neurons from 6 mice)	Inci + saline (14 neurons from 6 mice)		$P > 0.9999$	
	Naive + Remi (14 neurons from 6 mice)	Inci + Remi (14 neurons from 6 mice)		$P = 0.002$	
	Inci + saline (14 neurons from 6 mice)	Inci + Remi (14 neurons from 6 mice)		$P = 0.0467$	
Figure 4G	ACSF (8 mice)	Mibe (8 mice)	Two-way RM ANOVA with post hoc Bonferroni's test	$P = 0.002$	$F_{(1,14)} = 14.34$
	BL			$P > 0.9999$	
	D1			$P = 0.0102$	
	D2			$P = 0.1875$	
	D3			$P > 0.9999$	
	D4			$P > 0.9999$	
Figure 4K	AAV-control (4 mice)	AAV-RNAi (5 mice)	Unpaired Student's *t*-test	$P = 0.0178$	$t_{(7)} = 3.08$
Figure 4N	AAV-control (24 neurons from 8 mice)	AAV-RNAi (24 neurons from 8 mice)	Linear mixed models with post hoc Bonferroni's test	$P < 0.0001$	$F_{(1,622.864)}= 267.89$
Figure 4O	AAV-control (24 neurons from 8 mice)	AAV-RNAi (24 neurons from 8 mice)	Nested *t*-test analysis	$P < 0.0001$	$t_{(46)} = 6.265$
Figure 4Q	AAV-control (33 neurons from 11 mice)	AAV-RNAi (33 neurons from 11 mice)	Linear mixed models with post hoc Bonferroni's test	$P < 0.0001$	$F_{(1,333)} = 53.601$
Figure 4R	AAV-control (33 neurons from 11 mice)	AAV-RNAi (33 neurons from 11 mice)	Nested *t*-test analysis	$P = 0.0004$	$t_{(20)} = 4.215$
Figure 4S	AAV-control (10 mice)	AAV-RNAi (8 mice)	Two-way RM ANOVA with post hoc Bonferroni's test	$P < 0.0001$	$F_{(1,16)} = 148.2$
	BL			$P > 0.9999$	
	D1			$P < 0.0001$	
	D2			$P < 0.0001$	
	D3			$P = 0.0002$	
	D4			$P = 0.0085$	
Figure 5F	Glu&GFP/ GFP（5 slices from 5 mice）	GABA&GFP/ GFP (5 slices from 5 mice)	Unpaired Student's *t*-test	$P < 0.0001$	$t_{(8)} = 32.36$
Figure 5M	EYFP (13 neurons from 6 mice)	eNpHR3.0 (13 neurons from 6 mice)	Linear mixed models with Bonferroni post hoc analysis	$P < 0.0001$	$F_{(1,74)} = 22.81$
	Light on		Nested *t*-test analysis	$P = 0.001$	$t_{(10)} = 4.944$

Figure 5P	eNpHR3.0+ AAV-control (25 neurons from 8 mice)	eNpHR3.0+ AAV-RNAi (32 neurons from 8 mice)	Linear mixed models with Bonferroni post hoc analysis	$P < 0.0001$	$F_{(1,190.74)} = 26.171$
	Light on		Nested *t*-test analysis	$P < 0.0001$	$t_{(14)} = 10.43$
Figure 5U	BL (16 neurons from 8 mice)	Light on (16 neurons from 8 mice)	Paired Student's *t*-test	$P = 0.0002$	$t_{(15)} = 4.775$
Figure 5V	BL (16 neurons from 8 mice)	Light on (16 neurons from 8 mice)	Paired Student's *t*-test	$P = 0.8212$	$t_{(15)} = 0.2537$
Figure 6B			Linear mixed models with post hoc Bonferroni's test	$P < 0.0001$	$F_{(3,956.954)} = 18.748$
	Naive + Saline (23 neurons from 8 mice)	Naive + Remi (25 neurons from 8 mice)		$P > 0.999$	
	Naive + Saline (23 neurons from 8 mice)	Inci + saline (23 neurons from 8 mice)		$P > 0.999$	
	Naive + Remi (25 neurons from 8 mice)	Inci + Remi (25 neurons from 8 mice)		$P < 0.0001$	
	Inci + saline (23 neurons from 8 mice)	Inci + Remi (25 neurons from 8 mice)		$P = 0.002$	
Figure 6C			Nested one-way ANOVA with post hoc Bonferroni's test	$P = 0.0003$	$F_{(3,28)} = 8.543$
	Naive + Saline (23 neurons from 8 mice)	Naive + Remi (25 neurons from 8 mice)		$P > 0.9999$	
	Naive + Saline (23 neurons from 8 mice)	Inci + saline (23 neurons from 8 mice)		$P > 0.9999$	
	Naive + Remi (25 neurons from 8 mice)	Inci + Remi (25 neurons from 8 mice)		$P = 0.0008$	
	Inci + saline (23 neurons from 8 mice)	Inci + Remi (25 neurons from 8 mice)		$P = 0.0022$	
Figure 6G			Nested one-way ANOVA with post hoc Bonferroni's test	$P = 0.0117$	$F_{(3,28)} = 4.4$
	Naive + Saline (159 neurons from 8mice)	Naive + Remi (159 neurons from 8mice)		$P > 0.9999$	
	Naive + Saline (159 neurons from 8mice)	Inci + saline (154 neurons from 8mice)		$P > 0.9999$	
	Naive + Remi (159 neurons from 8mice)	Inci + Remi (154 neurons from 8mice)		$P = 0.0489$	
	Inci + saline (154 neurons from 8mice)	Inci + Remi (154 neurons from 8mice)		$P = 0.0464$	
Figure 6H			Nested one-way ANOVA with post hoc Bonferroni's test	$P = 0.0106$	$F_{(3,28)} = 4.505$

	Naive + Saline (159 neurons from 8mice)	Naive + Remi (159 neurons from 8mice)		$P > 0.9999$	
	Naive + Saline (159 neurons from 8mice)	Inci + saline (154 neurons from 8mice)		$P > 0.9999$	
	Naive + Remi (159 neurons from 8mice)	Inci + Remi (154 neurons from 8mice)		$P = 0.033$	
	Inci + saline (154 neurons from 8mice)	Inci + Remi (154 neurons from 8mice)		$P = 0.017$	
Figure 6N			Linear mixed models with post hoc Bonferroni's test	$P < 0.0001$	$F_{(3,575.662)} = 9.436$
	Naive + saline (26-27 neurons from 8 mice)	Naive + Remi (23-28 neurons from 8 mice)		$P > 0.9999$	
	Naive + saline (26-27 neurons from 8 mice)	Inci + saline (30-48 neurons from 8 mice)		$P > 0.9999$	
	Naive + Remi (23-28 neurons from 8 mice)	Inci + Remi (22-28 neurons from 8 mice)		$P < 0.0001$	
	Inci + saline (30-48 neurons from 8 mice)	Inci + Remi (22-28 neurons from 8 mice)		$P < 0.0001$	
	BL		Nested one-way ANOVA with post hoc Bonferroni's test	$P = 0.8199$	$F_{(3,28)} = 0.3075$
	D1		Nested one-way ANOVA with post hoc Bonferroni's test	$P < 0.0001$	$F_{(3,28)} = 30.58$
	Inci + saline (30 neurons from 8 mice)	Inci + Remi (28 neurons from 8 mice)		$P < 0.0001$	
	D2		Nested one-way ANOVA with post hoc Bonferroni's test	$P < 0.0001$	$F_{(3,28)} = 25.17$
	Inci + saline (25 neurons from 8 mice)	Inci + Remi (24 neurons from 8 mice)		$P < 0.0001$	
	D3		Nested one-way ANOVA with post hoc Bonferroni's test	$P = 0.5081$	$F_{(3,28)} = 10.19$
	D4		Nested one-way ANOVA with post hoc Bonferroni's test	$P = 0.6344$	$F_{(3,28)} = 30.58$
Figure 7D (left)	ACSF (8 slices from 5 mice)	Mibe (8 slices from 5 mice)	Unpaired Student's *t*-test	$P = 0.6518$	$t_{(14)} = 0.4611$
Figure 7D (right)	ACSF (8 slices from 5 mice)	Mibe (8 slices from 5 mice)	Unpaired Student's *t*-test	$P < 0.0001$	$t_{(14)} = 17.61$
Figure 7F	ACSF	Mibe	Linear mixed models	$P < 0.0001$	$F_{(1,348.99)} = 67.193$

	(20 neurons from 8 mice)	(15 neurons from 7 mice)	with post hoc Bonferroni's test		
Figure 7G	ACSF (20 neurons from 8 mice)	Mibe (15 neurons from 7 mice)	Nested *t*-test analysis	$P = 0.0002$	$t_{(33)} = 4.207$
Figure 7J	AAV-control (25-33 neurons from 8 mice)	AAV-RNAi (43-49 neurons from 8 mice)	Linear mixed models with post hoc Bonferroni's test	$P = 0.002$	$F_{(1,270.918)} = 18.013$
	BL		Nested *t*-test analysis	$P = 0.6212$	$t_{(14)} = 0.8593$
	D1		Nested *t*-test analysis	$P < 0.0001$	$t_{(14)} = 6.040$
	D2		Nested *t*- test analysis	$P < 0.0001$	$t_{(14)} = 5.823$
	D3		Nested *t*-test analysis	$P = 0.0009$	$t_{(14)} = 4.471$
	D4		Nested *t*-test analysis	$P = 0.6344$	$t_{(14)} = 1.138$
Figure 7O	GFP (5 neurons from 5 mice)	hM4Di-GFP (5 neurons from 5 mice)	Linear mixed models with post hoc Bonferroni's test	$P < 0.0001$	$F_{(1,188)} = 118.596$
Figure 7Q	GFP (23 neurons from 7 mice)	hM4Di-GFP (23 neurons from 8 mice)	Linear mixed models with post hoc Bonferroni's test	$P < 0.0001$	$F_{(1,460.808)} = 39.677$
Figure 7R	GFP (23 neurons from 7 mice)	hM4Di-GFP (23 neurons from 8 mice)	Nested *t*-test analysis	$P = 0.0003$	$t_{(13)} = 4.954$
Figure 7S	GFP (9 mice)	hM4Di-GFP (9 mice)	Two-way RM ANOVA with post hoc Bonferroni's test	$P < 0.0001$	$F_{(1,16)} = 64.69$
	BL			$P > 0.9999$	
	D1			$P = 0.0006$	
	D2			$P = 0.0039$	
	D3			$P = 0.0123$	
	D4			$P = 0.0267$	
Figure 7U (CPA)	GFP (10 mice)	hM4Di-GFP (9 mice)	Unpaired Student's *t*-test	$P = 0.231$	$t_{(17)} = 1.242$
Supplemental Figure 1B	Naïve + saline (8 mice)	Naïve + Remi (8 mice)	Two-way RM ANOVA with post hoc Bonferroni's test	$P = 0.6182$	$F_{(1,14)} = 0.2597$
	BL			$P > 0.9999$	
	D1			$P > 0.9999$	
	D2			$P > 0.9999$	
	D3			$P > 0.9999$	
	D4			$P > 0.9999$	
Supplemental Figure 1C	Naïve + saline (8 mice)	Naïve + Remi (8 mice)	Two-way RM ANOVA with post hoc Bonferroni's test	$P = 0.9676$	$F_{(1,14)} = 0.0017$
	BL			$P > 0.9999$	
	D1			$P > 0.9999$	
	D2			$P > 0.9999$	
	D3			$P > 0.9999$	
	D4			$P > 0.9999$	
Supplemental Figure 1D	Naïve + saline (10 mice)	Naïve + Remi (10 mice)	Two-way RM ANOVA with post hoc Bonferroni's test	$P = 0.1972$	$F_{(1,18)} = 1.793$
	BL			$P > 0.9999$	
	D1			$P > 0.9999$	
	D2			$P > 0.9999$	
	D3			$P > 0.9999$	
	D4			$P > 0.9999$	
Supplemental Figure 1E	Naïve + saline (10 mice)	Naïve + Remi (10 mice)	Two-way RM ANOVA with post hoc	$P = 0.7982$	$F_{(1,18)} = 0.0673$

			Bonferroni's test		
	BL			$P > 0.9999$	
	D1			$P > 0.9999$	
	D2			$P > 0.9999$	
	D3			$P > 0.9999$	
	D4			$P > 0.9999$	
Supplemental Figure 1F	Naïve + saline (10 mice)	Naïve + Remi (10 mice)	Two-way RM ANOVA with post hoc Bonferroni's test	$P = 0.899$	$F_{(1,18)} = 0.0165$
	BL			$P > 0.9999$	
	D1			$P > 0.9999$	
	D2			$P > 0.9999$	
	D3			$P > 0.9999$	
	D4			$P > 0.9999$	
Supplemental Figure 1G	Naïve + saline (10 mice)	Naïve + Remi (10 mice)	Two-way RM ANOVA with post hoc Bonferroni's test	$P = 0.7074$	$F_{(1,18)} = 0.1455$
	BL			$P > 0.9999$	
	D1			$P > 0.9999$	
	D2			$P > 0.9999$	
	D3			$P > 0.9999$	
	D4			$P > 0.9999$	
Supplemental Figure 2B	Saline (10 mice)	CFA (10 mice)	Two-way RM ANOVA with post hoc Bonferroni's test	$P < 0.0001$	$F_{(1,18)} = 134.3$
	BL			$P > 0.9999$	
	D3			$P = 0.0001$	
	D6			$P < 0.0001$	
	D9			$P = 0.0045$	
Supplemental Figure 2C	Saline (10 mice)	CFA (10 mice)	Two-way RM ANOVA with post hoc Bonferroni's test	$P = 0.3962$	$F_{(1,18)} = 0.7554$
	BL			$P > 0.9999$	
	D3			$P > 0.9999$	
	D6			$P = 0.2309$	
	D9			$P = 0.7076$	
Supplemental Figure 2D	Saline (10 mice)	CFA (10 mice)	Two-way RM ANOVA with post hoc Bonferroni's test	$P = 0.0005$	$F_{(1,18)} = 18.14$
	BL			$P > 0.9999$	
	D3			$P = 0.0009$	
	D6			$P = 0.5054$	
	D9			$P > 0.9999$	
Supplemental Figure 2E	Saline (10 mice)	CFA (10 mice)	Two-way RM ANOVA with post hoc Bonferroni's test	$P = 0.5618$	$F_{(1,18)} = 0.3494$
	BL			$P > 0.9999$	
	D3			$P > 0.9999$	
	D6			$P > 0.9999$	
	D9			$P > 0.9999$	
Supplemental Figure 2G	CFA + saline (10 mice)	CFA + Remi (10 mice)	Two-way RM ANOVA with post hoc Bonferroni's test	$P = 0.5155$	$F_{(1,18)} = 0.4400$
	BL			$P > 0.9999$	
	D3			$P = 0.6462$	
	D4			$P > 0.9999$	
	D5			$P > 0.9999$	
	D8			$P > 0.9999$	

Supplemental Figure 2H	CFA + saline (10 mice)	CFA + Remi (10 mice)		Two-way RM ANOVA with post hoc Bonferroni's test	$P < 0.0001$	$F_{(1,18)} = 39.59$
	BL				$P > 0.9999$	
	D1				$P = 0.0002$	
	D2				$P < 0.0001$	
	D3				$P = 0.0155$	
	D4				$P > 0.9999$	
	D5				$P > 0.9999$	
Supplemental Figure 2I	CFA + saline (10 mice)	CFA + Remi (10 mice)		Two-way RM ANOVA with post hoc Bonferroni's test	$P = 0.5325$	$F_{(1,18)} = 0.405$
	BL				$P > 0.9999$	
	D3				$P > 0.9999$	
	D4				$P > 0.9999$	
	D5				$P = 0.5056$	
	D8				$P > 0.9999$	
Supplemental Figure 2J	CFA + saline (10 mice)	CFA + Remi (10 mice)		Two-way RM ANOVA with post hoc Bonferroni's test	$P = 0.8284$	$F_{(1,18)} = 0.0484$
	BL				$P > 0.9999$	
	D1				$P > 0.9999$	
	D2				$P = 0.5305$	
	D3				$P > 0.9999$	
	D4				$P > 0.9999$	
	D5				$P > 0.9999$	
Supplemental Figure 3C (left)	Naïve + saline (9 slices from 5 mice)	Naïve +Remi (9 slices from 5 mice)		Unpaired Student's *t*-test	$P = 0.3561$	$t_{(16)} = 0.9502$
Supplemental Figure 3C (right)	Naïve + saline (9 slices from 5 mice)	Naïve +Remi (9 slices from 5 mice)		Unpaired Student's *t*-test	$P = 0.5839$	$t_{(16)} = 0.5589$
Supplemental Figure 3E	Inci + saline (9 slices from 5 mice)	Inci +Remi (9 slices from 5 mice)		Unpaired Student's *t*-test	$P < 0.0001$	$t_{(16)} = 6.98$
Supplemental Figure 3G (left)	Inci + saline (5 slices from 5 mice)	Inci +Remi (5 slices from 5 mice)		Unpaired Student's *t*-test	$P = 0.4951$	$t_{(8)} = 0.7147$
Supplemental Figure 3G (right)	Inci + saline (9 slices from 5 mice)	Inci +Remi (9 slices from 5 mice)		Unpaired Student's *t*-test	$P < 0.0001$	$t_{(16)} = 15.22$
Supplemental Figure 5D	BL (39 neurons from 8 mice)	Stimulus (40 neurons from 8 mice)	Post-stimulus (42 neurons from 8 mice)	Nested one-way ANOVA with post hoc Bonferroni's test	$P = 0.0068$	$F_{(2,21)} = 6.388$
	BL	Stimulus			$P = 0.0062$	
	BL	Post-stimulus			$P = 0.7097$	
	Stimulus	Post-stimulus			$P = 0.09$	
Supplemental	BL (39	Stimulus	Post-	Nested one-way	$P = 0.0115$	$F_{(2,21)} = 5.559$

Figure 5E	neurons from 8 mice)	(40 neurons from 8 mice)	stimulus (42 neurons from 8 mice)	ANOVA with post hoc Bonferroni's test		
	BL	Stimulus			$P = 0.01$	
	BL	Post-stimulus			$P = 0.5838$	
	Stimulus	Post-stimulus			$P = 0.1715$	
Supplemental Figure 5F	BL (39 neurons from 8 mice)	Stimulus (40 neurons from 8 mice)	Post-stimulus (42 neurons from 8 mice)	Nested one-way ANOVA with post hoc Bonferroni's test	$P = 0.0062$	$F_{(2,21)} = 6.55$
	BL	Stimulus			$P = 0.005$	
	BL	Post-stimulus			$P = 0.4134$	
	Stimulus	Post-stimulus			$P = 0.1393$	
Supplemental Figure 5G	BL (39 neurons from 8 mice)	Stimulus (40 neurons from 8 mice)	Post-stimulus (42 neurons from 8 mice)	Nested one-way ANOVA with post hoc Bonferroni's test	$P = 0.1620$	$F_{(2,21)} = 1.988$
	BL	Stimulus			$P = 0.5808$	
	BL	Post-stimulus			$P = 0.1934$	
	Stimulus	Post-stimulus			$P > 0.9999$	
Supplemental Figure 6B				Nested one-way ANOVA with post hoc Bonferroni's test	$P < 0.0001$	$F_{(3,260)} = 12.72$
	Naive + Saline (43 neurons from 8 mice)	Naive + Remi (47 neurons from 8 mice)			$P > 0.9999$	
	Naive + Saline (43 neurons from 8 mice)	Inci + saline (91 neurons from 8 mice)			$P > 0.9999$	
	Naive + Remi (47 neurons from 8 mice)	Inci + Remi (91 neurons from 8 mice)			$P < 0.0001$	
	Inci + saline (91 neurons from 8 mice)	Inci + Remi (83 neurons from 8 mice)			$P < 0.0001$	
Supplemental Figure 6C				Nested one-way ANOVA with post hoc Bonferroni's test	$P = 0.0011$	$F_{(3,28)} = 7.063$
	Naive + Saline (43 neurons from 8 mice)	Naive + Remi (47 neurons from 8 mice)			$P > 0.9999$	
	Naive + Saline (43 neurons from 8 mice)	Inci + saline (91 neurons from 8 mice)			$P > 0.9999$	
	Naive + Remi (47 neurons from 8 mice)	Inci + Remi (91 neurons from 8 mice)			$P = 0.03$	
	Inci + saline (91 neurons from 8 mice)	Inci + Remi (83 neurons from 8 mice)			$P = 0.0032$	
Supplemental Figure 6D				Nested one-way ANOVA with post hoc Bonferroni's test	$P = 0.0006$	$F_{(3,28)} = 7.775$

	Naive + Saline (43 neurons from 8 mice)	Naive + Remi (47 neurons from 8 mice)		$P > 0.9999$	
	Naive + Saline (43 neurons from 8 mice)	Inci + saline (91 neurons from 8 mice)		$P > 0.9999$	
	Naive + Remi (47 neurons from 8 mice)	Inci + Remi (91 neurons from 8 mice)		$P = 0.0013$	
	Inci + saline (91 neurons from 8 mice)	Inci + Remi (83 neurons from 8 mice)		$P = 0.0035$	
Supplemental Figure 6E			Nested one-way ANOVA with post hoc Bonferroni's test	$P = 0.0926$	$F_{(3,28)} = 2.363$
	Naive + Saline (43 neurons from 8 mice)	Naive + Remi (47 neurons from 8 mice)		$P > 0.9999$	
	Naive + Saline (43 neurons from 8 mice)	Inci + saline (91 neurons from 8 mice)		$P > 0.9999$	
	Naive + Remi (47 neurons from 8 mice)	Inci + Remi (91 neurons from 8 mice)		$P = 0.3778$	
	Inci + saline (91 neurons from 8 mice)	Inci + Remi (83 neurons from 8 mice)		$P = 0.4137$	
Supplemental Figure 6G			Nested one-way ANOVA with post hoc Bonferroni's test	$P = 0.0043$	$F_{(3,36)} = 5.223$
	Naive + Saline (25 neurons from 10 mice)	Naive + Remi (25 neurons from 10 mice)		$P > 0.9999$	
	Naive + Saline (25 neurons from 10 mice)	Inci + saline (28 neurons from 10 mice)		$P > 0.9999$	
	Naive + Remi (25 neurons from 10 mice)	Inci + Remi (30 neurons from 10 mice)		$P = 0.0278$	
	Inci + saline (28 neurons from 10 mice)	Inci + Remi (30 neurons from 10 mice)		$P = 0.0489$	
Supplemental Figure 6H			Nested one-way ANOVA with post hoc Bonferroni's test	$P = 0.0057$	$F_{(3,104)} = 4.423$
	Naive + Saline (25 neurons from 10 mice)	Naive + Remi (25 neurons from 10 mice)		$P > 0.9999$	
	Naive + Saline (25 neurons from 10 mice)	Inci + saline (28 neurons from 10 mice)		$P > 0.9999$	
	Naive + Remi (25 neurons from 10 mice)	Inci + Remi (30 neurons from 10 mice)		$P = 0.0225$	
	Inci + saline (28 neurons from 10 mice)	Inci + Remi (30 neurons from 10 mice)		$P = 0.0107$	
Supplemental Figure 7E			One-way ANOVA with post hoc Bonferroni's	$P < 0.0001$	$F_{(3,32)} = 18.74$

			test		
	Naive + saline (8 mice)	Naive + Remi (8 mice)		$P > 0.9999$	
	Naive + saline (8 mice)	Inci + saline (10 mice)		$P = 0.0014$	
	Naive + Remi (8 mice)	Inci + Remi (10 mice)		$P < 0.0001$	
	Inci + saline (10 mice)	Inci + Remi (10 mice)		$P = 0.6003$	
Supplemental Figure 8B	ACSF (13 neurons from 6 mice)	Mibe (13 neurons from 6 mice)	Linear mixed models with post hoc Bonferroni's test	$P < 0.0001$	$F_{(1,143.65)} = 33.348$
Supplemental Figure 8E	ACSF (15 neurons from 7 mice)	Mibe (13 neurons from 6 mice)	Linear mixed models with post hoc Bonferroni's test	$P = 0.015$	$F_{(1,138)} = 17.244$
Supplemental Figure 8F	ACSF (15 neurons from 7 mice)	Mibe (13 neurons from 6 mice)	Nested *t*-test analysis	$P = 0.0094$	$t_{(26)} = 2.807$
Supplemental Figure 8H	ACSF (9 mice)	Mibe (9 mice)	Two-way RM ANOVA with post hoc Bonferroni's test	$P < 0.0001$	$F_{(1,16)} = 48.92$
	BL			$P > 0.9999$	
	D1			$P < 0.0001$	
	D2			$P < 0.0001$	
	D3			$P < 0.0001$	
	D4			$P = 0.0024$	
Supplemental Figure 8J	eNpHR3.0+ ACSF (8 mice)	eNpHR3.0+ Mibe (8 mice)	Two-way RM ANOVA with post hoc Bonferroni's test	$P = 0.0001$	$F_{(1,14)} = 26.49$
	BL			$P = 0.4741$	
	Light on			$P = 0.0004$	
	Light off			$P > 0.9999$	
Supplemental Figure 8K	EYFP + ACSF (7 mice)	EYFP + Mibe (7 mice)	Two-way RM ANOVA with post hoc Bonferroni's test	$P = 0.0884$	$F_{(1,12)} = 3.439$
	BL			$P > 0.9999$	
	Light on			$P = 0.8422$	
	Light off			$P > 0.9999$	
Supplemental Figure 9F	eNpHR3.0 + AAV-control (10 mice)	eNpHR3.0 + AAV-RNAi (8 mice)	Two-way RM ANOVA with post hoc Bonferroni's test	$P < 0.0001$	$F_{(1,16)} = 28.16$
	BL			$P > 0.9999$	
	Light on			$P < 0.0001$	
	Light off			$P = 0.0004$	
Supplemental Figure 10B	Inci + saline (6 mice)	Inci + Sufen (6 mice)	Two-way RM ANOVA with post hoc Bonferroni's test	$P = 0.3149$	$F_{(1,10)} = 1.119$
	BL			$P > 0.9999$	
	D1			$P > 0.9999$	
	D2			$P > 0.9999$	
	D3			$P > 0.9999$	
	D4			$P > 0.9999$	
Supplemental Figure 10C	Inci + saline (6 mice)	Inci + Sufen (6 mice)	Two-way RM ANOVA with post hoc Bonferroni's test	$P = 0.6408$	$F_{(1,10)} = 0.2315$
	BL			$P > 0.9999$	
	D1			$P > 0.9999$	
	D2			$P > 0.9999$	
	D3			$P > 0.9999$	

	D4			$P > 0.9999$	
Supplemental Figure 10D	Inci + saline (10 mice)	Inci + Sufen (10 mice)	Two-way RM ANOVA with post hoc Bonferroni's test	$P = 0.5929$	$F_{(1,18)} = 0.2962$
	BL			$P > 0.9999$	
	D1			$P > 0.9999$	
	D2			$P > 0.9999$	
	D3			$P > 0.9999$	
	D4			$P > 0.9999$	
Supplemental Figure 10E	Inci + saline (10 mice)	Inci + Sufen (10 mice)	Two-way RM ANOVA with post hoc Bonferroni's test	$P = 0.8085$	$F_{(1,18)} = 0.0604$
	BL			$P > 0.9999$	
	D1			$P > 0.9999$	
	D2			$P > 0.9999$	
	D3			$P > 0.9999$	
	D4			$P > 0.9999$	
Supplemental Figure 10G	Inci + saline (10 mice)	Inci + Sufen (10 mice)	Two-way RM ANOVA with post hoc Bonferroni's test	$P = 0.8808$	$F_{(1,18)} = 0.0231$
	BL			$P > 0.9999$	
	D3			$P > 0.9999$	
	D4			$P > 0.9999$	
	D5			$P > 0.9999$	
	D8			$P > 0.9999$	
Supplemental Figure 10H	Inci + saline (10 mice)	Inci + Sufen (10 mice)	Two-way RM ANOVA with post hoc Bonferroni's test	$P = 0.4192$	$F_{(1,18)} = 0.6835$
	BL			$P > 0.9999$	
	D1			$P > 0.9999$	
	D2			$P > 0.9999$	
	D3			$P > 0.9999$	
	D4			$P > 0.9999$	
	D5			$P > 0.9999$	
Supplemental Figure 10I	Inci + saline (10 mice)	Inci + Sufen (10 mice)	Two-way RM ANOVA with post hoc Bonferroni's test	$P = 0.4062$	$F_{(1,18)} = 0.7234$
	BL			$P > 0.7009$	
	D3			$P > 0.9999$	
	D4			$P > 0.9999$	
	D5			$P > 0.9999$	
	D8			$P > 0.9999$	
Supplemental Figure 10J	Inci + saline (10 mice)	Inci + Sufen (10 mice)	Two-way RM ANOVA with post hoc Bonferroni's test	$P = 0.6469$	$F_{(1,18)} = 0.2171$
	BL			$P > 0.9999$	
	D1			$P > 0.9999$	
	D2			$P > 0.9999$	
	D3			$P > 0.6992$	
	D4			$P > 0.9999$	
	D5			$P > 0.9999$	
Supplemental Figure 11B	Inci + saline (9 slices from 5 mice)	Inci + Sufen (9 slices from 5 mice)	Unpaired Student's *t*-test	$P = 0.1331$	$t_{(16)} = 1.583$
Supplemental Figure 11D (left)	Inci + saline (5 slices from 5 mice)	Inci + Sufen (5 slices from 5 mice)	Unpaired Student's *t*-test	$P = 0.5122$	$t_{(8)} = 0.6858$
Supplemental	Inci + saline	Inci + Sufen	Unpaired Student's *t*-	$P = 0.2241$	$t_{(16)} = 1.265$

Figure 11D (right)	(9 slices from 5 mice)	(9 slices from 5 mice)	test		
Supplemental Figure 11F (left)	Inci + saline (54-78 neurons from 8 mice)	Inci + Sufen (58-72 neurons from 8 mice)	Linear mixed models with post hoc Bonferroni's test	$P = 0.974$	$F_{(1,727)} = 0.026$
Supplemental Figure 11F (right)	Inci + saline (54-78 neurons from 8 mice)	Inci + Sufen (58-72 neurons from 8 mice)	Linear mixed models with post hoc Bonferroni's test	$P = 0.917$	$F_{(1,727)} = 0.034$
Supplemental Figure 11J	Inci + saline (9 slices from 5 mice)	Inci + Sufen (9 slices from 5 mice)	Unpaired Student's *t*-test	$P = 0.7707$	$t_{(16)} = 0.2964$
Supplemental Figure 11L (left)	Inci + saline (5 slices from 5 mice)	Inci + Sufen (5 slices from 5 mice)	Unpaired Student's *t*-test	$P = 0.4748$	$t_{(8)} = 0.7499$
Supplemental Figure 11L (right)	Inci + saline (9 slices from 5 mice)	Inci + Sufen (9 slices from 5 mice)	Unpaired Student's *t*-test	$P = 0.7991$	$t_{(16)} = 0.2588$
Supplemental Figure 11N (left)	Inci + saline (60-70 neurons from 8 mice)	Inci + Sufen (56-67 neurons from 8 mice)	Linear mixed models with post hoc Bonferroni's test	$P = 0.732$	$F_{(1,623.5)} = 3.349$
Supplemental Figure 11N (right)	Inci + saline (65-70 neurons from 8 mice)	Inci + Sufen (56-68 neurons from 8 mice)	Linear mixed models with post hoc Bonferroni's test	$P = 0.604$	$F_{(1,623.5)} = 4.155$
Supplemental Figure 13C	EYFP (7 slices from 5 mice)	eNpHR3.0 (7 slices from 5 mice)	Unpaired Student's *t*-test	$P < 0.0001$	$t_{(12)} = 19.16$
Supplemental Figure 13E (left)	EYFP (8 slices from 5 mice)	eNpHR3.0 (8 slices from 5 mice)	Unpaired Student's *t*-test	$P = 0.9526$	$t_{(14)} = 0.0604$
Supplemental Figure 13E (right)	EYFP (8 slices from 5 mice)	eNpHR3.0 (8 slices from 5 mice)	Unpaired Student's *t*-test	$P < 0.0001$	$t_{(14)} = 13.12$
Supplemental Figure 14F			One-way ANOVA with post hoc Bonferroni's test	$P < 0.0001$	$F_{(3,16)} = 262.1$
	ACSF (5 neurons from 5 mice)	TTX (5 neurons from 5 mice)		$P < 0.0001$	
	4-AP + TTX (5 neurons from 5 mice)	4-AP + TTX + DNQX (5 neurons from 5 mice)		$P < 0.0001$	
Supplemental Figure 15C (left)	Naïve + saline (9 slices from 5 mice)	Naïve + Remi (9 slices from 5 mice)	Unpaired Student's *t*-test	$P = 0.8939$	$t_{(16)} = 0.1356$
Supplemental Figure 15C (right)	Naïve + saline (9 slices from 5 mice)	Naïve + Remi (9 slices from 5 mice)	Unpaired Student's *t*-test	$P = 0.6636$	$t_{(16)} = 0.4431$
Supplemental Figure 15E	Inci + saline (9 slices from 5 mice)	Inci + Remi (9 slices from 5 mice)	Unpaired Student's *t*-test	$P < 0.0001$	$t_{(16)} = 14.34$
Supplemental Figure 15G (left)	Inci + saline (5 slices from 5 mice)	Inci + Remi (5 slices from 5 mice)	Unpaired Student's *t*-test	$P = 0.7644$	$t_{(8)} = 0.3101$
Supplemental	Inci + saline	Inci + Remi	Unpaired Student's *t*-	$P < 0.0001$	$t_{(16)} = 14.32$

Figure 15G (right)	(9 slices from 5 mice)	(9 slices from 5 mice)	test		
Supplemental Figure 16C			Nested one-way ANOVA with post hoc Bonferroni's test	$P = 0.0254$	$F_{(3,28)} = 3.609$
	Naive + Saline (23 neurons from 8 mice)	Naive + Remi (25 neurons from 8 mice)		$P > 0.9999$	
	Naive + Saline (23 neurons from 8 mice)	Inci + saline (23 neurons from 8 mice)		$P > 0.9999$	
	Naive + Remi (25 neurons from 8 mice)	Inci + Remi (25 neurons from 8 mice)		$P = 0.1913$	
	Inci + saline (23 neurons from 8 mice)	Inci + Remi (25 neurons from 8 mice)		$P = 0.0352$	
Supplemental Figure 16D			Nested one-way ANOVA with post hoc Bonferroni's test	$P = 0.7716$	$F_{(3,28)} = 0.3751$
	Naive + Saline (23 neurons from 8 mice)	Naive + Remi (25 neurons from 8 mice)		$P > 0.9999$	
	Naive + Saline (23 neurons from 8 mice)	Inci + saline (23 neurons from 8 mice)		$P > 0.9999$	
	Naive + Remi (25 neurons from 8 mice)	Inci + Remi (25 neurons from 8 mice)		$P > 0.9999$	
	Inci + saline (23 neurons from 8 mice)	Inci + Remi (25 neurons from 8 mice)		$P > 0.9999$	
Supplemental Figure 17L	BL (25 neurons from 6 mice)	Stimulus (27 neurons from 6 mice)<br>Post-stimulus (27 neurons from 6 mice)	Nested one-way ANOVA with post hoc Bonferroni's test	$P = 0.0068$	$F_{(2,21)} = 6.388$
	BL	Stimulus		$P = 0.0123$	
	BL	Post-stimulus		$P = 0.4912$	
	Stimulus	Post-stimulus		$P = 0.2138$	
Supplemental Figure 18B	Inci + saline (9 slices from 5 mice)	Inci + Remi (9 slices from 5 mice)	Unpaired Student's *t*-test	$P < 0.0001$	$t_{(16)} = 8.899$
Supplemental Figure 18D (left)	Inci + saline (5 slices from 5 mice)	Inci + Remi (5 slices from 5 mice)	Unpaired Student's *t*-test	$P = 0.9166$	$t_{(8)} = 0.1081$
Supplemental Figure 18D (right)	Inci + saline (9 slices from 5 mice)	Inci + Remi (9 slices from 5 mice)	Unpaired Student’s *t*-test	$P < 0.0001$	$t_{(16)} = 10.20$
Supplemental Figure 18G			Linear mixed models with post hoc Bonferroni's test	$P < 0.0001$	$F_{(3,976)} = 77.103$
	Naïve + saline (27 neurons from 8 mice)	Naive + Remi (23 neurons from 8 mice)		$P > 0.999$	
	Naïve + saline (27 neurons from 8 mice)	Inci + saline (30 neurons from 8 mice)		$P = 0.101$	

	Naïve + Remi (23 neurons from 8 mice)	Inci + Remi (18 neurons from 6 mice)		$P = 0.001$	
	Inci + saline (30 neurons from 8 mice)	Inci + Remi (18 from 6 mice)		$P = 0.013$	
Supplemental Figure 18H			Nested one-way ANOVA with post hoc Bonferroni's test	$P = 0.0429$	$F_{(3,26)} = 3.127$
	Naïve + saline (27 neurons from 8 mice)	Naive + Remi (23 neurons from 8 mice)		$P > 0.9999$	
	Naïve + saline (27 neurons from 8 mice)	Inci + saline (30 neurons from 8 mice)		$P > 0.9999$	
	Naïve + Remi (23 neurons from 8 mice)	Inci + Remi (18 neurons from 6 mice)		$P = 0.0595$	
	Inci + saline (30 neurons from 8 mice)	Inci + Remi (18 from 6 mice)		$P > 0.9999$	
Supplemental Figure 19E			Nested one-way ANOVA with post hoc Bonferroni's test	$P < 0.0001$	$F_{(3,28)} = 12.94$
	Naïve + saline (139 neurons from 8 mice)	Naive + Remi (136 neurons from 8 mice)		$P > 0.9999$	
	Naïve + saline (139 neurons from 8 mice)	Inci + saline (145 neurons from 8 mice)		$P = 0.6537$	
	Naïve + Remi (136 neurons from 8 mice)	Inci + Remi (133 neurons from 8 mice)		$P < 0.0001$	
	Inci + saline (145 neurons from 8 mice)	Inci + Remi (133 from 8 mice)		$P = 0.0039$	
Supplemental Figure 19F			Nested one-way ANOVA with post hoc Bonferroni's test	$P < 0.0001$	$F_{(3,549)} = 9.787$
	Naïve + saline (139 neurons from 8 mice)	Naive + Remi (136 neurons from 8 mice)		$P > 0.9999$	
	Naïve + saline (139 neurons from 8 mice)	Inci + saline (145 neurons from 8 mice)		$P = 0.2473$	
	Naïve + Remi (136 neurons from 8 mice)	Inci + Remi (133 neurons from 8 mice)		$P = 0.0002$	
	Inci + saline (145 neurons from 8 mice)	Inci + Remi (133 from 8 mice)		$P = 0.0129$	
Supplemental Figure 19I			Linear mixed models with post hoc Bonferroni's test	$P < 0.0001$	$F_{(3,500.102)} = 10.572$
	Naive + saline (26-30 neurons from 6 mice)	Naive + Remi (25-28 neurons from 6 mice)		$P > 0.999$	
	Naive + saline	Inci + Saline		$P = 0.646$	

	(26-30 neurons from 6 mice)	(28-31 neurons from 6 mice)			
	Naive + Remi (25-28 neurons from 6 mice)	Inci + Remi (28-32 neurons from 6 mice)		$P = 0.039$	
	Inci + Saline (28-31 neurons from 6 mice)	Inci + Remi (28-32 neurons from 6 mice)		$P = 0.047$	
	BL		Nested one-way ANOVA with post hoc Bonferroni's test	$P = 0.9648$	$F_{(3,20)} = 0.093$
	D1		Nested one-way ANOVA with post hoc Bonferroni's test	$P < 0.0001$	$F_{(3,20)} = 12.722$
	Inci + saline (30 neurons from 6 mice)	Inci + Remi (28 neurons from 6 mice)		$P = 0.011$	
	Naïve+ Remi (27 neurons from 6 mice)	Inci + Remi (28 neurons from 6 mice)		$P < 0.0001$	
	D2		Nested one-way ANOVA with post hoc Bonferroni's test	$P = 0.0139$	$F_{(3,20)} = 5.281$
	Inci + saline (28 neurons from 6 mice)	Inci + Remi (29 neurons from 6 mice)		$P = 0.1713$	
	Naïve+ Remi (27 neurons from 6 mice)	Inci + Remi (29 neurons from 6 mice)		$P = 0.013$	
	D3		Nested one-way ANOVA with post hoc Bonferroni's test	$P = 0.691$	$F_{(3,20)} = 2.132$
	D4		Nested one-way ANOVA with post hoc Bonferroni's test	$P = 0. 8753$	$F_{(3,20)} = 0.157$
Supplemental Figure 20B	Inci + saline (9 slices from 5 mice)	Inci + Sufen (9 slices from 5 mice)	Unpaired Student's *t*-test	$P = 0.679$	$t_{(16)} = 0.4215$
Supplemental Figure 20D (left)	Inci + saline (5 slices from 5 mice)	Inci + Sufen (5 slices from 5 mice)	Unpaired Student's *t*-test	$P = 0.8911$	$t_{(8)} = 0.1414$
Supplemental Figure 20D (right)	Inci + saline (9 slices from 5 mice)	Inci + Sufen (9 slices from 5 mice)	Unpaired Student's *t*-test	$P = 0.1892$	$t_{(16)} = 1.371$
Supplemental Figure 20F	Inci + saline (16-23 neurons from 6 mice)	Inci + Sufen (20-25 neurons from 6 mice)	Linear mixed models with post hoc Bonferroni's test	$P = 0.88$	$F_{(1,208.999)} = 0.023$
Supplemental Figure 20J	Inci + saline (9 slices from 5 mice)	Inci + Sufen (9 slices from 5 mice)	Unpaired Student's *t*-test	$P = 0.5373$	$t_{(16)} = 0.6305$
Supplemental Figure 20L (left)	Inci + saline (5 slices from 5 mice)	Inci + Sufen (5 slices from 5 mice)	Unpaired Student's *t*-test	$P = 0.556$	$t_{(8)} = 0.6145$
Supplemental Figure 20L	Inci + saline (9 slices from	Inci + Sufen (9 slices from	Unpaired Student's *t*-test	$P = 0.5554$	$t_{(16)} = 0.6024$

(right)	5 mice)	5 mice)			
Supplemental Figure 20N	Inci + saline (20-23 neurons from 6 mice)	Inci + Sufen (15-22 neurons from 6 mice)	Linear mixed models with post hoc Bonferroni's test	$P = 0.794$	$F_{(1, 300.01)} = 0.036$
Supplemental Figure 21D (left)	ACSF (8 slices from 5 mice)	Mibe (8 slices from 5 mice)	Unpaired Student's *t*-test	$P = 0.257$	$t_{(14)} = 1.182$
Supplemental Figure 21D (right)	ACSF (8 slices from 5 mice)	Mibe (8 slices from 5 mice)	Unpaired Student's *t*-test	$P < 0.0001$	$t_{(14)} = 5.448$
Supplemental Figure 21F	ACSF (20 neurons from 8 mice)	Mibe (20 neurons from 8 mice)	Linear mixed models with post hoc Bonferroni's test	$P < 0.0001$	$F_{(1, 468.97)} = 19.741$
Supplemental Figure 21G	ACSF (20 neurons from 8 mice)	Mibe (20 neurons from 8 mice)	Nested *t*-test analysis	$P < 0.0001$	$t_{(38)} = 6.306$
Supplemental Figure 22B	ACSF (10 mice)	MUS (10 mice)	Two-way RM ANOVA with post hoc Bonferroni's test	$P = 0.0221$	$F_{(1,18)} = 6.273$
	BL			$P > 0.9999$	
	D1			$P = 0.0419$	
	D2			$P = 0.0480$	
	D3			$P > 0.9999$	
	D4			$P > 0.9999$	
Supplemental Figure 22E	ACSF (20 neurons from 7 mice)	MUS (20 neurons from 7 mice)	Linear mixed models with post hoc Bonferroni's test	$P = 0.001$	$F_{(1,399.858)} = 10.338$
Supplemental Figure 22F	ACSF (20 neurons from 7 mice)	MUS (20 neurons from 7 mcie)	Nested *t*-test analysis	$P = 0.0002$	$t_{(12)} = 5.417$
Supplemental Figure 23D	GFP (8 mice)	hM4Di (8 mice)	Two-way RM ANOVA with post hoc Bonferroni's test	$P < 0.0001$	$F_{(1,14)} = 50.55$
	BL			$P > 0.9999$	
	D1			$P < 0.0001$	
	D2			$P = 0.0041$	
	D3			$P =0.2836$	
	D4			$P > 0.9999$	
Supplemental Figure 23F	hM4Di-CNO (6 neurons from 6 mice)		One sample *t*-test	$P < 0.0001$	$t_{(5)} = 18.74$
Supplemental Figure 23H	GFP (29 neurons from 7 mice)	hM4Di (16 neurons from 6 mice)	Linear mixed models with post hoc Bonferroni's test	$P < 0.0001$	$F_{(1,448)} = 75.877$
Supplemental Figure 23I	GFP (29 neurons from 7 mice)	hM4Di (16 neurons from 6 mice)	Nested *t*-test analysis	$P < 0.0001$	$t_{(11)} = 7.556$
Supplemental Figure 24C			Two-way RM ANOVA with post hoc Bonferroni's test	$P = 0.0014$	$F_{(2,21)} = 9.162$
	BL (8 neurons from 8 mice)	Remi (8 neurons from 8 mice)		$P = 0.0019$	

	BL (8 neurons from 8 mice)	Washout (8 neurons from 8 mice)		$P = 0.6858$	
Supplemental Figure 24D			One-way RM ANOVA with post hoc Bonferroni's test	$P < 0.0001$	$F_{(2,14)} = 73.19$
	BL (8 neurons from 8 mice)	Remi (8 neurons from 8 mice)		$P < 0.0001$	
	BL (8 neurons from 8 mice)	Washout (8 neurons from 8 mice)		$P > 0.9999$	
Supplemental Figure 24E			One-way RM ANOVA with post hoc Bonferroni's test	$P = 0.0026$	$F_{(2,14)} = 9.425$
	BL (8 neurons from 8 mice)	Remi (8 neurons from 8 mice)		$P = 0.0034$	
	BL (8 neurons from 8 mice)	Washout (8 neurons from 8 mice)		$P > 0.9999$	
Supplemental Figure 24F			One-way RM ANOVA with post hoc Bonferroni's test	$P < 0.0001$	$F_{(2,14)} = 66.93$
	BL (8 neurons from 8 mice)	Remi (8 neurons from 8 mice)		$P < 0.0001$	
	BL (8 neurons from 8 mice)	Washout (8 neurons from 8 mice)		$P > 0.9999$	
Supplemental Figure 24I			Two-way RM ANOVA with post hoc Bonferroni's test	$P < 0.0001$	$F_{(2,27)} = 42.01$
	BL (10 neurons from 10 mice)	Remi (10 neurons from 10 mice)		$P = 0.001$	
	BL (10 neurons from 10 mice)	Washout (10 neurons from 10 mice)		$P = 0.024$	
Supplemental Figure 24J			One-way RM ANOVA with post hoc Bonferroni's test	$P < 0.0001$	$F_{(2,18)} = 53.87$
	BL (10 neurons from 10 mice)	Remi (10 neurons from 10 mice)		$P < 0.0001$	
	BL (10 neurons from 10 mice)	Washout (10 neurons from 10 mice)		$P = 0.0294$	
Supplemental Figure 24K			One-way RM ANOVA with post hoc Bonferroni's test	$P < 0.0001$	$F_{(2,18)} = 18.17$
	BL (10 neurons from 10 mice)	Remi (10 neurons from 10 mice		$P < 0.0001$	
	BL (10 neurons from 10 mice)	Washout (10 neurons from 10 mice)		$P = 0.5059$	
Supplemental Figure 24L			One-way RM ANOVA with post hoc	$P = 0.0032$	$F_{(2,18)} = 8.016$

			Bonferroni's test		
	BL (10 neurons from 10 mice)	Remi (10 neurons from 10 mice)		$P = 0.016$	
	BL (10 neurons from 10 mice)	Washout (10 neurons from 10 mice)		$P = 0.8411$	
Supplemental Figure 25C			Two-way RM ANOVA with post hoc Bonferroni's test	$P = 0.3924$	$F_{(2,39)} = 0.9582$
	BL (14 neurons from 14 mice)	Remi (14 neurons from 14 mice)		$P = 0.2477$	
	BL (14 neurons from 14 mice)	Washout (14 neurons from 14 mice)		$P = 0.3444$	
Supplemental Figure 25D			One-way RM ANOVA with post hoc Bonferroni's test	$P = 0.1929$	$F_{(2,26)} = 1.754$
	BL (14 neurons from 14 mice)	Remi (14 neurons from 14 mice)		$P = 0.3543$	
	BL (14 neurons from 14 mice)	Washout (14 neurons from 14 mice)		$P = 0.1723$	
Supplemental Figure 25E			One-way RM ANOVA with post hoc Bonferroni's test	$P = 0.0012$	$F_{(2,26)} = 8.795$
	BL (14 neurons from 14 mice)	Remi (14 neurons from 14 mice)		$P = 0.0006$	
	BL (14 neurons from 14 mice)	Washout (14 neurons from 14 mice)		$P = 0.1831$	
Supplemental Figure 25F			One-way RM ANOVA with post hoc Bonferroni's test	$P = 0.0374$	$F_{(2,26)} = 3.74$
	BL (14 neurons from 14 mice)	Remi (14 neurons from 14 mice)		$P = 0.0701$	
	BL (14 neurons from 14 mice)	Washout (14 neurons from 14 mice)		$P > 0.9999$	
Supplemental Figure 25I			Two-way RM ANOVA with post hoc Bonferroni's test	$P = 0.7537$	$F_{(2,39)} = 0.2848$
	BL (14 neurons from 14 mice)	Remi (14 neurons from 14 mice)		$P = 0.4457$	
	BL (14 neurons from 14 mice)	Washout (14 neurons from 14 mice)		$P = 0.7833$	
Supplemental Figure 25J			One-way RM ANOVA with post hoc Bonferroni's test	$P = 0.2118$	$F_{(2,26)} = 1.649$
	BL (14 neurons from 14 mice)	Remi (14 neurons from 14 mice)		$P = 0.1857$	

	BL (14 neurons from 14 mice)	Washout (14 neurons from 14 mice)		$P = 0.4043$	
Supplemental Figure 25K			One-way RM ANOVA with post hoc Bonferroni's test	$P = 0.0002$	$F_{(2,26)} = 11.62$
	BL (14 neurons from 14 mice)	Remi (14 neurons from 14 mice)		$P = 0.0001$	
	BL (14 neurons from 14 mice)	Washout (14 neurons from 14 mice)		$P = 0.0133$	
Supplemental Figure 25L			One-way RM ANOVA with post hoc Bonferroni's test	$P = 0.0771$	$F_{(2, 26)} = 2.832$
	BL (14 neurons from 14 mice)	Remi (14 neurons from 14 mice)		$P = 0.2644$	
	BL (14 neurons from 14 mice)	Washout (14 neurons from 14 mice)		$P = 0.8805$	
Supplemental Figure 26B	Sham (10 mice)	SNI (10 mice)	Two-way RM ANOVA with post hoc Bonferroni's test	$P < 0.0001$	$F_{(1,18)} = 1050$
	BL			$P > 0.9999$	
	D3			$P < 0.0001$	
	D6			$P < 0.0001$	
	D9			$P = 0.0001$	
	D14			$P < 0.0001$	
	D21			$P < 0.0001$	
	D28			$P < 0.0001$	
	D35			$P < 0.0001$	
Supplemental Figure 26C	Sham (10 mice)	SNI (10 mice)	Two-way RM ANOVA with post hoc Bonferroni's test	$P < 0.0001$	$F_{(1,18)} = 169.3$
	BL			$P > 0.9999$	
	D3			$P = 0.0028$	
	D6			$P < 0.0001$	
	D9			$P = 0.0025$	
	D14			$P = 0.0013$	
	D21			$P = 0.0033$	
	D28			$P = 0.0203$	
	D35			$P = 0.0017$	
Supplemental Figure 26E	Sham (32 neurons from 7 mice)	SNI (59 neurons from 10 mice)	Nested *t*-test analysis	$P = 0.0195$	$t_{(15)} = 2.615$
Supplemental Figure 26F	Sham (32 neurons from 7 mice)	SNI (59 neurons from 10 mice)	Nested *t*-test analysis	$P = 0.0302$	$t_{(15)} = 2.393$
Supplemental Figure 26G	Sham (32 neurons from 7 mice)	SNI (59 neurons from 10 mice)	Nested *t*-test analysis	$P = 0.0297$	$t_{(15)} = 5.77$
Supplemental Figure 26I	Sham (52 neurons from 7 mice)	SNI (56 neurons from 7 mice)	Nested *t*-test analysis	$P = 0.0007$	$t_{(106)} = 3.51$
Supplemental Figure 26K	Sham (8 mice)	SNI (8 mice)	Two-way RM ANOVA with post hoc Bonferroni's test	$P < 0.0001$	$F_{(1,14)} = 100.3$
	BL			$P > 0.9999$	

	SNI			$P > 0.9999$	
	0.5h			$P = 0.1797$	
	1h			$P = 0.17$	
	1.5h			$P = 0.0198$	
	2h			$P < 0.0001$	
	2.5h			$P = 0.0039$	
	3h			$P = 0.0045$	
	3.5h			$P = 0.0753$	
	4h			$P > 0.9999$	
Supplemental Figure 26L	Sham (8 mice)	SNI (8 mice)	Two-way RM ANOVA with post hoc Bonferroni's test	$P < 0.0001$	$F_{(1,14)} = 209.8$
	BL			$P > 0.9999$	
	SNI			$P > 0.9999$	
	0.5 h			$P = 0.0480$	
	1 h			$P = 0.0001$	
	1.5 h			$P < 0.0001$	
	2 h			$P = 0.0004$	
	2.5 h			$P = 0.0008$	
	3 h			$P = 0.0024$	
	3.5 h			$P > 0.9999$	
	4 h			$P > 0.9999$	

Supplemental Table 2 KEY RESOURCES TABLE

Resource or Reagent	Source	Identifier
**Racterial and Virus Strains**		
rAAV-CaMKIIa-GCaMp6m-WPRE-hGH pA	BrainVTA	Cat# PT-0111
rAAV-CaMKIIa-GCaMp6f-WPRE-hGH pA	BrainVTA	Cat#PT-0119
rAAV-EF1α-DIO-ΔRVG-WPRE-hGH pA	BrainVTA	Cat#PT-0023
rAAV-EF1α-DIO-H2B-EGFP-T2A-TVA-WPRE-hGH pA	BrainVTA	Cat#PT-0021
RV-ENVA-ΔG-dsRed	BrainVTA	Cat#R01002
rAAV-Ef1α-DIO-ChR2-mCherry-WPRE-pA	BrainVTA	Cat#PT-0002
rAAV-Ef1α-DIO-eNpHR3.0-EYFP-WPRE-pA	BrainVTA	Cat#PT-0006
rAAV-Ef1α-DIO-EYFP-WPRE-pA	BrainVTA	Cat#PT-0012
rAAV-CaMKIIa-hM4D(Gi)-EGFP-WPRE-hGH pA	BrainVTA	Cat# PT-0524
rAAV-CaMKIIa-EGFP-WPRE-hGH pA	BrainVTA	Cat# PT-0290
rAAV-EF1α-DIO-mCherry-hGH pA	BrainVTA	Cat# PT-0013
rAAV-EF1α-DIO-EGFP-hGH pA	BrainVTA	Cat# PT-0795
rAAV-hSyn-EGFP-P2A-CRE-WPRE-hGH pA	BrainVTA	Cat# PT-0156
rAAV-CaMKIIa-mCherry-5'miR-30a-shRNA (Ca_v3.1)-3'-miR30a-WPREs	BrainVTA	N/A
rAAV-CaMKIIa-mCherry-5'miR-30a-shRNA-3'-miR30a-WPREs	BrainVTA	N/A
rAAV-CaMKIIa-eNpHR3.0-EYFP-WPRE-hGH-pA	BrainVTA	Cat# PT-0008
**Antibodies**		
Rabbit anti-glutamate	Sigma	Cat# G6642
Rabbit anti-c-Fos	SYSY	Cat#226003
Rabbit anti-GABA	Sigma	Cat#A2052
Mouse anti-glutamate	Sigma	Cat#G9282
Mouse beta-actin	Absin	Cat#abs137975
Rabbit Na, K-ATPase	CST	Cat#3010S
Rabbit anti-Ca_v 3.1	Thermo	Cat#PA577311
Goat anti-mouse	Jackson	Cat#115-035-003
Goat anti-rabbit	Invitrogen	Cat#31466
ECL reagent	Thermo	Cat#32106
Donkey anti-rabbit IgG Alexa 488	Invitrogen	Cat#A21206
Donkey anti-mouse IgG Alexa 594	Invitrogen	Cat#A21203
Donkey anti-rabbit IgG Alexa 594	Invitrogen	Cat#A21207
Donkey anti-rabbit IgG Alexa 647	Invitrogen	Cat#A31573
**Chemicals, Peptides, and Recombinant Proteins**		
Mibefradil	Sigma	Cat#M5441
Muscimol	Sigma	Cat#2763-96-4
DAPI	Sigma	Cat#D9542
Clozapine-N-Oxide (CNO)	Sigma	Cat#C0832
Picrotoxin (PTX)	Sigma	Cat#R284556
Tetrodotoxin (TTX)	Tocris Bioscience	Cat#1069
6,7-dinitroquinoxaline-2,3-dione (DNQX)	Sigma	Cat#2379-57-9
CsCl	Sigma	Cat#7647-17-8
TEA-Cl	Sigma	Cat#56-34-8
4-AP	Sigma	Cat#20263-07-4
Carprofen	Sigma	Cat#PHR1452
Dexamethasone	MedChemExpress	Cat#HY-14686
Enrofloxacin	MedChemExpress	Cat#HY-B0502
Membrane and Cytoplasmic Protein Extraction kit	Sangon Biotech	Cat#C510005
Bicinchoninic acid	Thermo	Cat#23225
complete Freund's adjuvant (CFA)	Sigma	Cat#F5881
**Experimental models: Organisms/Strains**		
Mouse: C57BL/6J	Charles River	Stock#000064
Mouse: CaMKII-ires-Cre	The Jackson	Stock#005359

	Laboratory	
Mouse: Ai 14	The Jackson Laboratory	Stock#007914
**Software and Algorithms**		
Illustrator CS6	Adobe	https://www.adobe.com/products/illustrator.html
ZEN	Zeiss	https://www.zeiss.com/microscopy/us/products/microscope-software/zen-lite.html
Graphpad Prism 8.0	GraphPad software	https://www.graphpad.com/scientific-software/prism/
SPSS Statistics V26	SPSS Statistics software	https://www.ibm.com/analytics/spss-statistics-software
MatlabR2020b	MathWorks	https://www.mathworks.com/products/new_products/release2020b.html
Offline sorter Version 4	Plexon	https://plexon.com/software-downloads/
Neuroexplorer Version 5	Plexon	https://plexon.com/software-downloads/
Imagej	National Institutes of Health	https://imagej.net/imagej-wiki-static/fiji
Inper Studio	Inper Ltd.	https://www.inper.com/
EthoVision XT software	Noldus	https://www.noldus.com/ethovision-xt
**Others**		
Optogenetic fibers	Inper	N/A
Electrode wire for tetrode	California fine wire	N/A
